# Leveraging a reduced polyoxomolybdate-alkoxide cluster for the formation of a stable U(v) sandwich complex†

**DOI:** 10.1039/d4sc02644f

**Published:** 2024-06-17

**Authors:** Dominic Shiels, William W. Brennessel, Matthew R. Crawley, Ellen M. Matson

**Affiliations:** a Department of Chemistry, University of Rochester Rochester NY 14627 USA dshiels@ur.rochester.edu matson@chem.rochester.edu; b Department of Chemistry, University at Buffalo, The State University of New York Buffalo NY 14620 USA

## Abstract

The synthesis and characterization of a series of (TBA)_2_[M{Mo_5_O_13_(OMe)_4_NO}_2_] (M = Zr, Hf, Th, and U) sandwich complexes is reported. A preformed lacunary, Lindqvist-type, polyoxomolybdate-alkoxide cluster provides access to first examples of actinide-polyoxomolybdate sandwich complexes isolated under non-aqueous conditions. Incorporation of metal(iv) cations into this framework was found to “switch on” reversible redox chemistry at the {Mo_5_} ligands, with the Zr and Hf containing complexes accepting up to two electrons, while the Th and U derivates accommodate as many as four additional electrons. The enhancement of the redox properties of the cluster upon actinide incorporation is an exciting observation, presenting actinide “doping” as a novel approach for accessing functional redox-active materials. Oxidation of the uranium containing sandwich complex (TBA)_2_[U{Mo_5_O_13_(OMe)_4_NO}_2_], chemically or electrochemically, allows access to the U(v) centered species, which was characterized both spectroscopically and by single crystal X-ray diffraction. This represents the first example of a U(v)-polyoxometalate sandwich complex to be isolated and structurally characterized.

## Abstract 2

Actinide doping enhances redox chemistry of polyoxomolybdate-alkoxide clusters; the first isolable U(v) polyoxometalate cluster is reported.

## Graphical abstract

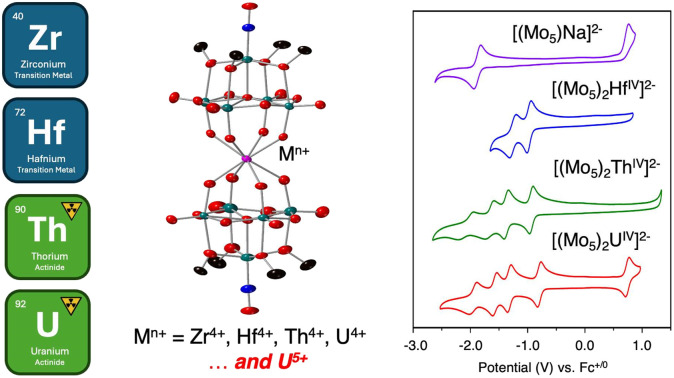

## Introduction

Complexation of actinides is a rich area of research due to the importance of selectively sequestering actinides from nuclear waste streams.^1–3^ More recently, this interest has expanded as researchers look to employ actinide centers, which possess a unique combination of large ionic radii and active use of 5f orbitals in bonding and catalysis, to access unique reactivity that is not open to transition metal containing materials.^4–6^ Polyoxometalates (POMs), which are anionic molecular metal oxide clusters typically based on tungsten(vi), molybdenum(vi), or vanadium(v), have become increasingly popular frameworks for studying the coordination preferences of both early and late actinides.^7–14^ The stability and ease by which these clusters crystallize has allowed detailed structural characterization of “An-POM” complexes, and for convenient comparison to other transition metal or lanthanide containing analogues.^15–18^ Furthermore, their large molecular weight (∼1000 to 20 000 g mol^−1^) allows stoichiometric reactions to be carried out with incredibly small quantities of the actinide starting material, a property which is vital when looking to study the transplutonic elements.^19,20^

One important limitation of almost all “An-POM” complexes studied to date is that they are almost exclusively synthesized and soluble in water.^21–23^ This limits the scope of solution-based studies, particularly in the context of electrochemistry where one is limited by the electrochemical window of water. This makes the likelihood of An-based redox events falling outside the range that can be reasonably studied significant.^24^ This is compounded by the fact that previous electrochemical investigations on [An(α-2-P_2_W_17_O_61_)_2_]^*n*−^ (An = Th, U, Np, and Pu, *n* = 16, An = Am, *n* = 17) complexes showed that the non-innocent nature of water was a contributing factor to the instability of U^5+^ in this framework.^25^ Attempted bulk electrolysis of the U^4+^ complex (U(v)/U(iv) couple at 0.55 V *vs.* AgCl) at 0.8 V (pH = 3.5 in an aqueous LiOAc/HClO_4_ buffer) led to the formation of uranyl(vi) acetate. A similar study by Termes and Pope showed that bulk electrolysis of [U(PW_11_O_39_)_2_]^10−^ (at 0.8 V *vs.* SCE, pH = 4.4) led to the transient formation of the oxidized U^5+^ species, as evidenced by electronic absorption spectroscopy.^26^ Historically, researchers have had hypothesized that the large polyoxometalate ligands would provide steric protection for a “nude” U^5+^ center. However, recent work by Colla *et al.* on a series of [M(PW_11_O_39_)_2_]^11−^ compounds suggests that complexes of this type are in equilibrium with [(H_2_O)_*x*_M(PW_11_O_39_)]^4−^ in water (evidenced by ^31^P NMR spectroscopy).^27^ Assuming a similar equilibrium is also active for [U(α-2-P_2_W_17_O_61_)_2_]^16−^ in aqueous solution, this could explain the instability of the oxidized products and, in the case of the Dawson derivative, the rapid formation of uranyl cations upon exposure to oxidizing potentials.

**Fig. 1:**
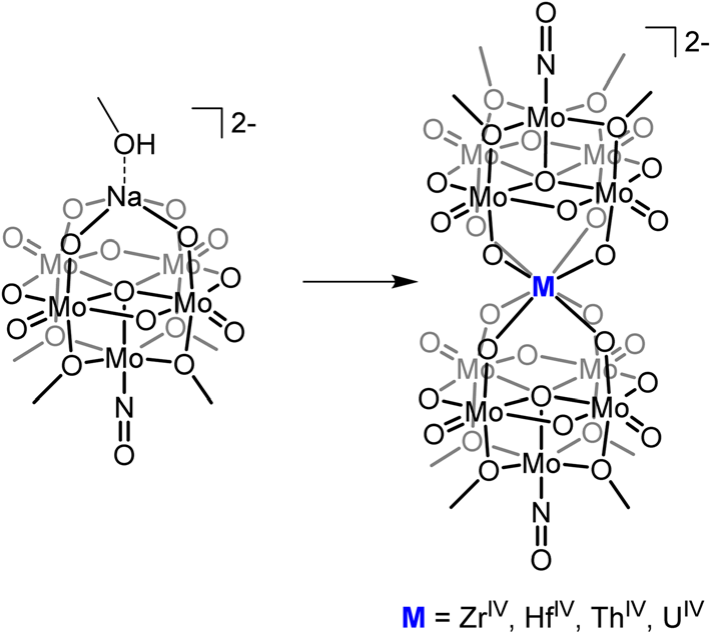
General schematic for the formation of sandwich complexes from 1-NaMo_5_ originally described by Villanneau^35^ and extended in this work.

The ability to isolate similar An(POM)_2_ complexes outside of water would allow exploitation of the advantages of using POM-based ligands (robust and high molecular weight) without the limitations of working in water. It is worth noting that the only examples of “An-POM” complexes prepared using non-aqueous methods come from Klemperer and co-workers, who bound [Cp_*x*_An]^4−*x*^ groups to Lindqvist-type POMs in organic solvents.^28,29^ An alternative, rational way to achieve formation of “An-POM” complexes soluble in organic solvent would be to take a preformed lacunary POM, or closely related polyoxomolybdate-alkoxide cluster, that is stable and soluble in non-aqueous media, and combine it with an appropriate source of the An center. With this in mind, we identified (TBA)_2_[Mo_5_O_13_(OMe)_4_NO][Na(MeOH)] (1-NaMo_5_, TBA = tetrabutylammonium) as a potential candidate for this role (Fig. 1). 1-NaMo_5_ was originally characterized by Proust and co-workers in 1993 and is a rare example of a lacunary Lindqvist structure.^30^ The reactivity of 1-NaMo_5_ towards a range of transition metals was investigated.^31–34^ In particular, Villanneau *et al.* explored the formation of a range of [M{Mo_5_O_13_(OMe)_4_NO}_2_]^*n*−^ sandwich complexes with large M^2+^ and M^3+^ cations (including Ca, Sr, Ba, Bi, Ce, and Eu), illustrating the potential for this lacunary Lindqvist cluster to support large metal cations.^35^

Herein we describe the synthesis and characterization of a series of M(iv) containing [M{Mo_5_O_13_(OMe)_4_NO}_2_]^2−^ sandwich complexes (M = Zr, Hf, Th, and U). The methodology, refined during the isolation of the Zr and Hf derivatives, was found to be high-yielding and versatile, allowing the use of both metal chlorides and metal alkoxides as sources of the heterometal. This approach was then used to extend the series to the actinide containing analogues. Characterization of the complexes by single crystal X-ray diffraction (SCXRD) revealed that the M(iv) centers adopt a square antiprismatic geometry, with the harder transition metal containing complexes forming shorter M–O bonds than the actinide congeners. Examination of the electrochemical properties of the series indicates that incorporation of a M(iv) center into the scaffold enhances the reducibility of the framework, with the effect most pronounced for the actinide containing sandwich complexes. The observation of a reversible of U(v)/U(iv) in the cyclic voltammogram (CV) of (TBA)_2_[U{Mo_5_O_13_(OMe)_4_NO}_2_] prompted oxidation experiments to be performed, with electrochemical bulk oxidation followed by electronic absorption spectroscopy giving preliminary evidence for the formation of the U(v) containing species. Chemical oxidation allowed for isolation and characterization of the first U(v) centered POM complex.

## Experimental

### General considerations

All air- and moisture-sensitive manipulations were carried out using a standard high vacuum line and Schlenk techniques, or in an MBraun inert atmosphere drybox containing an atmosphere of purified dinitrogen. All solids were dried under high vacuum in order to be brought into the glovebox. Solvents for air- and moisture-sensitive manipulations were dried and deoxygenated using a Glass Contour Solvent Purification System (Pure Process Technology, LLC) and stored over activated 4 Å molecular sieves (Fisher Scientific) prior to use. Deuterated solvents for NMR spectroscopy were purchased from Cambridge Isotope Laboratories and, after three freeze–pump–thaw cycles, were stored in the glovebox over activated 3 Å molecular sieves. (TBA)_4_[Mo_8_O_26_], (TBA)_2_[Mo_5_O_13_(OMe)_4_NO][Na(MeOH)], UCl_4_, and ThCl_4_·2DME were synthesized according to literature procedures.^30,36–38^ (TBA)_4_[Ba{Mo_5_O_13_(OMe)_4_NO}_2_] and (TBA)_3_[Bi{Mo_5_O_13_(OMe)_4_NO}_2_] were synthesized using the method of Villaneau and co-workers with Ba(OTf)_2_ and BiBr_3_ in place of BaCl_2_·2H_2_O and BiCl_3_ respectively.^35^ All the remaining chemicals were purchased from commercial sources (Fisher Scientific, VWR, and Sigma-Aldrich) and used without further purification.

### Safety considerations

**Caution!** Depleted uranium (primary isotope ^238^U) is a weak a-emitter (4.197 MeV) with a half-life of 4.47 × 10^9^ years, and ^232^Th is a weak a-emitter (4.082 MeV) with a half-life of 1.41 × 10^10^ years. Manipulations and reactions should be carried out in monitored fume hoods or in an inert atmosphere drybox in a radiation laboratory equipped with α and β counting equipment.

### Synthesis of (TBA)_2_[Zr(Mo_5_O_13_(OMe)_4_NO)_2_] (2-Zr(Mo_5_)_2_)

#### Method A

In a 20 mL scintillation vial, (TBA)_2_[Mo_5_O_13_(OMe)_4_NO][Na(MeOH)] (0.30 g, 0.22 mmol, 2 eq.) was dissolved in MeOH (7 mL) forming a dark purple solution. ZrCl_4_ (28 mg, 0.12 mmol, 1.1 eq.) was dissolved in MeOH (3 mL) and added slowly to the mixture. A blue/green precipitate formed immediately upon addition. The suspension was stirred for 15 minutes before filtering through a bed of celite (approx. 1 cm) and washing with MeOH (5 mL). The product was extracted with DCM until washings ran clear (approx. 10–15 mL). The solvent was removed under reduced pressure. The product (0.25 g, 88% yield) was found to be suitably pure at this point by ^1^H NMR spectroscopy and elemental analysis, however recrystallisation by slow evaporation of saturated solutions of the product dissolved in either MeCN or THF can be performed if desired. Blue/green single crystals were obtained by vapor diffusion of Et_2_O into a saturated solution of the product in MeCN. ^1^H NMR (500 MHz, CD_2_Cl_2_) *δ* 1.11 (t, *J* = 7.2 Hz, 24H), 1.56 (m, 16H), 1.81 (m, 16H), 3.32 (m, 16H), 4.73 (s, 24H). *λ*_max_ (MeCN) = 588 nm (*ε* = 99 mol^−1^ dm^3^ cm^−1^). Anal. calcd for C_40_H_96_N_4_Mo_10_O_36_Zr (mol. wt 2259.92 g mol^−1^): C, 21.26%; H, 4.28%; N, 2.48%. Found: C, 21.54%; H, 4.04%; N, 2.42%.

#### Method B

In a 20 mL scintillation vial, (TBA)_2_[Mo_5_O_13_(OMe)_4_NO][Na(MeOH)] (0.10 g, 0.070 mmol, 2 eq.) was suspended in THF (5 mL). The mixture was stirred while a solution of Zr(O^*t*^Bu)_4_ (14 mg, 0.036 mmol, 1 eq.) in THF (5 mL) was slowly added with no change. To this mixture trifluoroacetic acid (1.45 mL, 0.1 M solution in THF, 0.14 mmol, 4 eq.) was added dropwise. The suspended solid slowly dissolves and a homogenous green solution forms. The mixture was stirred for 30 minutes before the solvent was removed under vacuum. The blue/green residue was treated with MeOH (5 mL) forming a fine teal suspension. This mixture was passed through a bed of celite (approx. 1 cm thick) and washed with MeOH (3 mL). The solid was then extract with DCM until the until washings ran clear (approx. 10–15 mL). The solvent was removed under reduced pressure. The product (58 mg, 71% yield) was found to be suitably pure at this point by ^1^H NMR spectroscopy and elemental analysis but can be recrystallized as described above.

### Synthesis of (TBA)_2_[Hf(Mo_5_O_13_(OMe)_4_NO)_2_] (3-Hf(Mo_5_)_2_)

#### Method A

In a 20 mL scintillation vial, (TBA)_2_[Mo_5_O_13_(OMe)_4_NO][Na(MeOH)] (0.3 g, 0.22 mmol, 2 eq.) was dissolved in MeOH (7 mL) forming a dark purple solution. HfCl_4_ (38 mg, 0.12 mmol, 1.1 eq.) was dissolved in MeOH (3 mL) and added slowly to the mixture. A blue/green precipitate formed immediately upon addition. The suspension was stirred for a further 15 minutes before filtering through a bed of celite (approx. 1 cm) and washing with MeOH (5 mL). The product was extracted with DCM until washings ran clear (approx. 10–15 mL). The solvent was removed under reduced pressure. The product (0.22 g, 85% yield) was found to be suitably pure at this point by ^1^H NMR spectroscopy and elemental analysis, however recrystallisation by slow evaporation of saturated solutions of the product dissolved in either MeCN or THF can be performed if desired. Blue/green single crystals were obtained by vapor diffusion of Et_2_O into a saturated solution of the product in MeCN. ^1^H NMR (500 MHz, CD_2_Cl_2_) *δ* 1.11 (t, *J* = 7.3 Hz, 24H), 1.56 (m, 16H), 1.80 (m, 16H), 3.32 (m, 16H), 4.73 (s, 24H). *λ*_max_ (MeCN) = 592 nm (*ε* = 122 mol^−1^ dm^3^ cm^−1^). Anal. calcd for C_40_H_96_N_4_Mo_10_O_36_Hf (mol. wt 2347.19 g mol^−1^): C, 20.47%; H, 4.12%; N, 2.39%. Found: C, 20.52%; H, 4.23%; N, 2.27%.

#### Method B

In a 20 mL scintillation vial, (TBA)_2_[Mo_5_O_13_(OMe)_4_NO][Na(MeOH)] (0.1 g, 0.072 mmol, 2 eq.) was suspended in THF (5 mL). The mixture was stirred while a solution of Hf(O^*t*^Bu)_4_ (17 mg, 0.036 mmol, 1 eq.) in THF (5 mL) was slowly added with no change. To this mixture trifluoroacetic acid (1.45 mL, 0.1 M solution in THF, 0.14 mmol, 4 eq.) was added dropwise. The suspended solid slowly dissolves and a homogenous green solution forms. The mixture was stirred for 30 minutes before the solvent was removed under vacuum. The blue/green residue was treated with MeOH (5 mL) forming a fine teal suspension. This mixture was passed through a bed of celite (approx. 1 cm thick) and washed with MeOH (3 mL). The solid was then extract with DCM until the until washings ran clear (approx. 10–15 mL). The solvent was removed under reduced pressure. The product (59 mg, 69% yield) was found to be suitably pure at this point by ^1^H NMR spectroscopy and elemental analysis but can be recrystallized as described above.

### Synthesis of (TBA)_2_[Th(Mo_5_O_13_(OMe)_4_NO)_2_] (4-Th(Mo_5_)_2_)

In a 20 mL scintillation vial, (TBA)_2_[Mo_5_O_13_(OMe)_4_NO][Na(MeOH)] (0.3 g, 0.22 mmol, 2 eq.) was dissolved in MeOH (7 mL) forming a dark purple solution. ThCl_4_·2DME (66 mg, 0.12 mmol, 1.1 eq.) was dissolved in MeOH (3 mL) and added slowly to the mixture. A pale grey/green precipitate formed immediately upon addition. The suspension was stirred for a further 15 minutes before filtering through a bed of celite (approx. 1 cm) and washing with MeOH (5 mL). The product was extracted with DCM until washings ran clear (approx. 10–15 mL). The solvent was removed under reduced pressure. The product (0.23 g, 87% yield) was found to be suitably pure at this point by ^1^H NMR spectroscopy and elemental analysis, however recrystallisation by slow evaporation of saturated solutions of the product dissolved in either MeCN or THF can be performed if desired. Purple single crystals were obtained by vapor diffusion of Et_2_O into a saturated solution of the product in MeCN. ^1^H NMR (500 MHz, CD_2_Cl_2_) *δ* 1.11 (t, *J* = 7.3 Hz, 24H), 1.56 (m, 16H), 1.80 (m, 16H), 3.31 (m, 16H), 4.73 (s, 24H). *λ*_max_ (MeCN) = 572 nm (*ε* = 115 mol^−1^ dm^3^ cm^−1^). Anal. calcd for C_40_H_96_N_4_Mo_10_O_36_Th (mol. wt 2400.74 g mol^−1^): C, 20.01%; H, 4.03%; N, 2.33%. Found: C, 19.82%; H, 4.15%; N, 2.36%.

### Synthesis of (TBA)_2_[U(Mo_5_O_13_(OMe)_4_NO)_2_] (5-U(Mo_5_)_2_)

In a 20 mL scintillation vial, (TBA)_2_[Mo_5_O_13_(OMe)_4_NO][Na(MeOH)] (0.3 g, 0.22 mmol, 2 eq.) was dissolved in MeOH (7 mL) forming a dark purple solution. UCl_4_ (45 mg, 0.12 mmol, 1.1 eq.) was dissolved in MeOH (3 mL) and added slowly to the mixture. A brown precipitate formed immediately upon addition. The suspension was stirred for a further 15 minutes before filtering through a bed of celite (approx. 1 cm) and washing with MeOH (5 mL). The product was extracted with DCM until washings ran clear (approx. 10–15 mL). The solvent was removed under reduced pressure. The product (0.26 g, 92% yield) was found to be suitably pure at this point by ^1^H NMR spectroscopy and elemental analysis, however recrystallisation by slow evaporation of saturated solutions of the product dissolved in either MeCN or THF can be performed if desired. Brown single crystals were obtained by vapor diffusion of Et_2_O into a saturated solution of the product in MeCN. ^1^H NMR (500 MHz, CD_2_Cl_2_) *δ* −4.26 (s, 16H), −3.71 (s, 16H), −2.98 (s, 16H), −2.28 (m, 24H), 9.58 (s, 24H). *λ*_max_ (MeCN) = 682 nm (*ε* = 197 mol^−1^ dm^3^ cm^−1^), 1100 nm (*ε* = 66 mol^−1^ dm^3^ cm^−1^), 1156 nm (*ε* = 172 mol^−1^ dm^3^ cm^−1^) (broad and intense absorption at 400–650 nm also observed). Anal. calcd for C_40_H_96_N_4_Mo_10_O_36_U (mol. wt 2406.73 g mol^−1^): C, 19.96%; H, 4.02%; N, 2.33%. Found: C, 20.26%; H, 3.81%; N, 2.25%.

### Synthesis of (TBA)[U(Mo_5_O_13_(OMe)_4_NO)_2_] (6-U(Mo_5_)_2_)

In a 20 mL scintillation vial, (TBA)_2_[U(Mo_5_O_13_(OMe)_4_NO)_2_] (30 mg, 0.012 mmol, 1 eq.) was dissolved in MeCN (3 mL). This solution was then addition to a separate 20 mL scintillation vial containing [NO][PF_6_] (22 mg, 0.12 mmol, 10 eq.). The mixture was stirred vigorously, with a black/brown suspension quickly forming. The suspension was stirred for 3 minutes before it was passed through a bed of silica. The solid was washed with a small amount of MeCN (1 mL) before extracting with DCM (around 5 mL). The DCM was removed under reduced pressure to afford crude 6-U(Mo_5_)_2_ (21 mg, 78% yield). Brown single crystals were obtained by vapor diffusion of pentane into a saturated solution of the product in DCM. ^1^H NMR (500 MHz, CD_2_Cl_2_) *δ* 0.71 (s, 12H), 0.87 (s, 8H), 0.99 (s, 8H), 2.43 (s, 8H), 4.76 (s, 24H). *λ*_max_ (DCM) = 980 nm, 1160 nm, 1582 nm (broad and intense absorption at 400–700 nm also observed). Anal. calcd for C_24_H_60_N_3_Mo_10_O_36_U CH_3_CN (mol. wt. 2077.32 g mol^−1^): C, 15.03%; H, 3.06%; N, 2.70%. Found: C, 14.77%; H, 2.95%; N, 2.65%.

### Physical measurements

^1^H NMR spectra were recorded at room temperature on a 400 MHz Bruker AVANCE spectrometer or a 500 MHz Bruker AVANCE spectrometer locked on the signal of deuterated solvents. All chemical shifts are reported relative to the chosen deuterated solvent as a standard. ^1^H DOSY NMR spectra were recorded at room temperature (2 mM) on a 500 MHz JEOL JNM-ECZR spectrometer. The spectra were acquired using the Delta package using the bpp_ste_diffusion pulse sequence. The gradient strength *G* was varied in 16 steps from 0.3–28 G cm^−1^ to ensure at least ≈90% signal attenuation. All spectra were recorded without sample spinning with 32 K time-domain data points in the t2 dimension, with 16 transients for each gradient increment, and a delay time of 2 ms. The spectra were processed using the Jason software package. Cyclic voltammetry (CV) was performed using a three-electrode setup inside a nitrogen filled glove box (MBraun UniLab, USA) using a Bio-Logic SP 150 potentiostat/galvanostat and the EC-Lab software suite. The concentration of the cluster and the supporting electrolyte (TBAPF_6_) were kept at 1 mM and 100 mM respectively throughout all measurements. CVs were recorded using a 3 mm diameter glassy carbon working electrode (CH Instruments, USA), a Pt wire auxiliary electrode (CH Instruments, USA), and an Ag/Ag^+^ non-aqueous reference electrode with 0.01 M AgNO_3_ in 0.1 M TBAPF_6_ in acetonitrile (BASi, USA). CVs were *iR* compensated at 85% with impedance taken at 100 kHz using the ZIR tool included within the EC-Lab software. Bulk electrolysis experiments were performed in a H-cell with a glass frit separator (porosity = 10–16 μm, Pine Research, USA) using a Bio-Logic SP 150 potentiostat/galvanostat. In all experiments, an active species concentration of 1 mM was used. The working electrode compartment contained 10 mL of the active species with 0.1 M supporting electrolyte TBAPF_6_ in the desired solvent (MeCN or DCM), while the counter electrode compartment had 10 mL of 0.1 M supporting electrolyte in the same solvent (MeCN or DCM). A Pt mesh working electrode and a Pt wire counter electrode were used. Bulk electrolysis experiments were carried out using the chronoamperometry techniques available in EC-Lab software suite at constant potentials, selected from CV. Electronic absorption measurements were recorded at room temperature in anhydrous MeCN or DCM in sealed 1 cm quartz cuvettes using an Agilent Cary 6000i UV-vis-NIR spectrophotometer. Elemental analysis data were obtained from the Elemental Analysis Facility at the University of Rochester. Microanalysis samples were weighed with a PerkinElmer model AD6000 autobalance, and their compositions were determined with a PerkinElmer 2400 series II analyzer. Air-sensitive samples were handled in a VAC Atmospheres glovebox.

### X-ray crystallography

Crystals were placed onto a nylon loop and mounted on a Rigaku XtaLAB Synergy-S Dualflex diffractometer equipped with a HyPix-6000HE HPC area detector for data collection at 100.00(10) K. A preliminary set of cell constants and an orientation matrix were calculated from a small sampling of reflections.^39^ A short pre-experiment was run, from which an optimal data collection strategy was determined. The full data collection was carried out using a PhotonJet (Mo) X-ray source. After the intensity data were corrected for absorption, the final cell constants were calculated from the xyz centroids of the strong reflections from the actual data collection after integration.^39^ The structure was solved using SHELXT and refined using SHELXL.^40,41^ Most or all non-hydrogen atoms were assigned from the solution. Full-matrix least squares/difference Fourier cycles were performed which located any remaining non-hydrogen atoms. All non-hydrogen atoms were refined with anisotropic displacement parameters. All hydrogen atoms were placed in ideal positions and refined as riding atoms with relative isotropic displacement parameters.

## Results & discussion

**Fig. 2:**
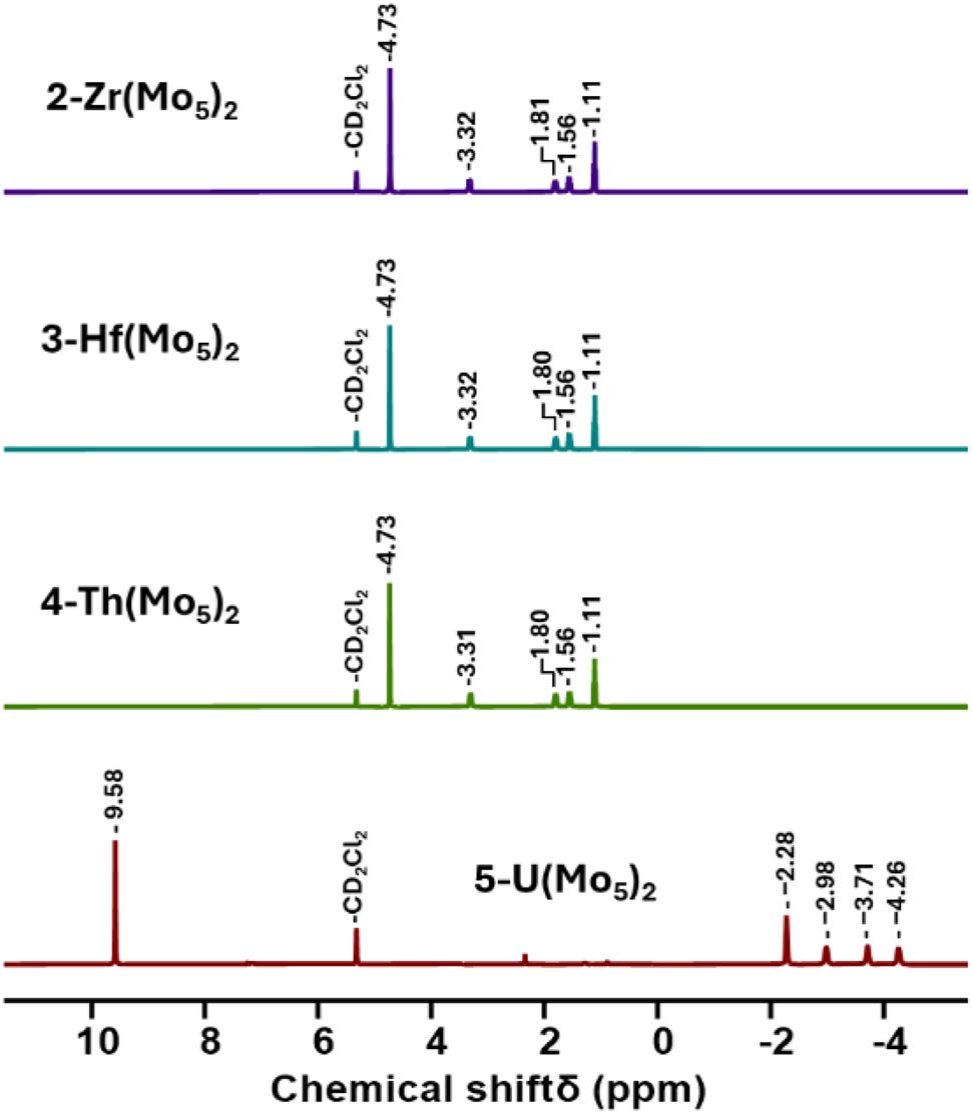
^1^H NMR spectra of the products obtained from reactions of 1-NaMo_5_ with approximately half an equivalent of a series of MCl_4_ salts (M = Zr, Hf, Th, and U).

Villanneau and co-workers previously described the treatment of (TBA)_2_[Mo_5_O_13_(OMe)_4_NO][Na(MeOH)] (1-NaMo_5_) with MX_2_ or MX_3_ salts in MeOH as a robust method for isolating [M{Mo_5_O_13_(OMe)_4_NO}_2_]^*n*−^ type sandwich complexes.^35^ Inspired by this approach, we sought to extend this methodology to MX_4_ salts, using Zr^4+^ and Hf^4+^ as model systems. To this end, 1-NaMo_5_ was dissolved in methanol (forming a purple solution) and was treated with approximately half an equivalent of MCl_4_, where M = Zr or Hf. The addition of the M^4+^ salt led to the immediate precipitation of a fine green powder. This contrasts the observations made by Villaneau, where a purple solution persists after treatment of 1-NaMo_5_ with MX_2_ or MX_3_ salts. The striking difference can be attributed to the reduced solubility of M^4+^ containing sandwich complexes in organic solvents, as they possess a lower negative charge than the M^2+^/M^3+^ containing analogues. The fine green solid can be isolated by filtration and extraction with DCM. After removal of the solvent, the solid was characterized by ^1^H NMR spectroscopy (Fig. 2). The spectra obtained from reactions with Zr^4+^ and Hf^4+^ (2-Zr(Mo_5_)_2_ and 3-Hf(Mo_5_)_2_) are almost identical, showing the expected presence of peaks associated with the TBA cations (1–3.5 ppm) and an additional peak 4.73 ppm assigned to the bridging alkoxide groups of [Mo_5_O_13_(OMe)_4_NO]^3−^ (Fig. 2).^35^ Integration of this peak *versus* the TBA peaks shows a 1 : 1 ratio of TBA : [Mo_5_O_13_(OMe)_4_NO]^3−^, consistent with the successful formation of (TBA)_2_[M{Mo_5_O_13_(OMe)_4_NO}_2_] (M = Zr, Hf). No evidence of the formation of a neutral [(L)_*x*_MMo_5_O_13_(OMe)_4_NO] species (*i.e.* the 1 : 1 complex) was observed by ^1^H NMR spectroscopy.

In order to gain a better understanding of the synthetic approach, the same products (2-Zr(Mo_5_)_2_ and 3-Hf(Mo_5_)_2_) were also targeted using an alternative method. Rather than exploiting MCl_4_ salts as the source of the heterometal, metal alkoxides were employed. Using this approach, 1-NaMo_5_ was suspended in THF and treated with half an equivalent of M(O^*t*^Bu)_4_ (M = Zr or Hf). Addition results in no obvious change, implying the alkoxide precursors are unreactive under these conditions. However, upon addition of two equivalents of acid (four with respect to the metal alkoxide) the solution began to turn green as the solid 1-NaMo_5_ reacted and the product dissolved. A homogenous green solution was obtained after around 30 minutes. The product can be isolated by removal of the solvent under vacuum and washing with MeOH to remove the sodium and tetrabutylammonium salt by-products. Characterization of the products *via*^1^H NMR spectroscopy shows an identical spectrum to that obtained from 1-NaMo_5_/MCl_4_ (see Fig. 2), suggesting that 2-Zr(Mo_5_)_2_/3-Hf(Mo_5_)_2_ can be obtained either using a salt metathesis approach or a protonolysis approach. This exemplifies the versatility of this reactivity, with the same products isolated regardless of the source of the heterometal.

**Fig. 3:**
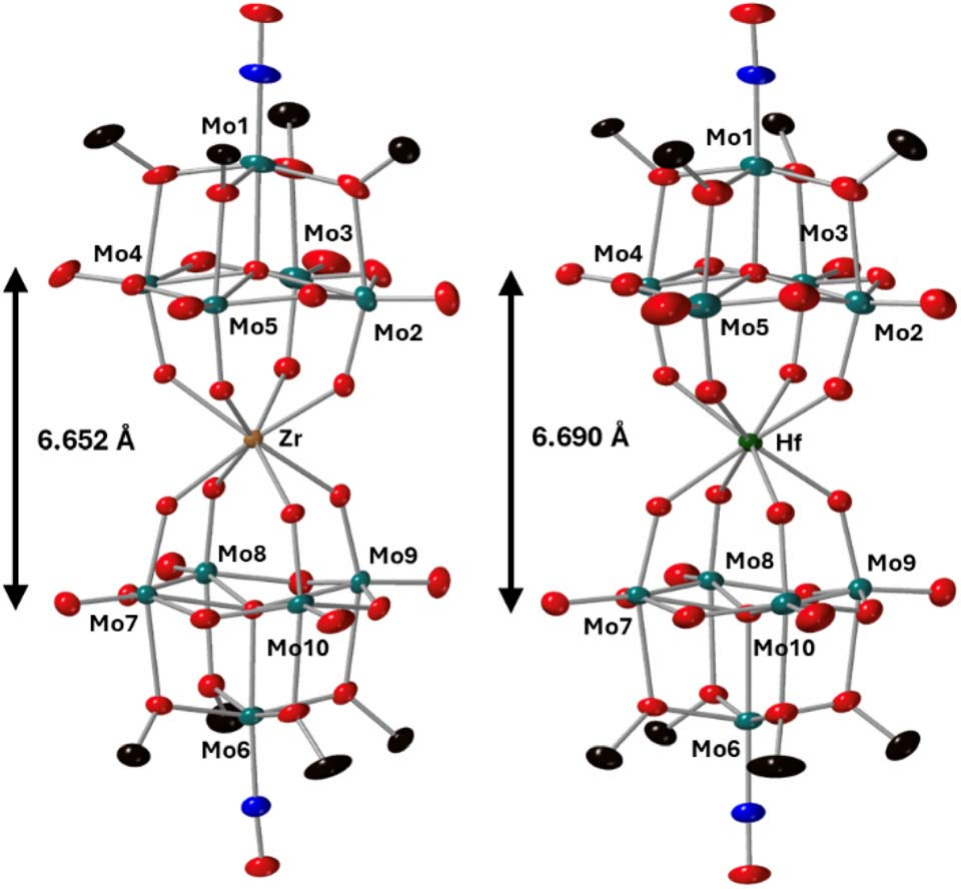
Single crystal X-ray diffraction structures of 2-Zr(Mo_5_)_2_ and 3-Hf(Mo_5_)_2_ with probability ellipsoids set at 50%. The tetrabutylammonium cations and some disorder has been masked for clarity. Detailed bond length data is given in the ESI.†

To further characterize the products of these reactions, single crystals of 2-Zr(Mo_5_)_2_ and 3-Hf(Mo_5_)_2_ were grown by vapor diffusion of diethyl ether into saturated solutions of the products in acetonitrile. X-ray diffraction experiments (Fig. 3) confirmed the successful isolation of (TBA)_2_[M{Mo_5_O_13_(OMe)_4_NO}_2_] sandwich complexes (where M = Zr and Hf, *i.e.*2-Zr(Mo_5_)_2_, 3-Hf(Mo_5_)_2_). The structures feature a central 8-coordinate metal ion adopting a square antiprismatic geometry between two [Mo_5_O_13_(OMe)_4_NO]^3−^ units. Irrespective of the central metal ion, the average bond lengths within these [Mo_5_O_13_(OMe)_4_NO]^3−^ units are relatively constant (see ESI Section 3†). The major differences arise in variations in the bond lengths between the [Mo_5_O_13_(OMe)_4_NO]^3−^ and the heterometal, and subsequently the distance between the respective anionic oxo-alkoxide units. For 2-Zr(Mo_5_)_2_ and 3-Hf(Mo_5_)_2_, the average M–O bond lengths are *ca.* 2.19 Å and 2.20 Å respectively. This is drastically, but expectedly, shorter than any of the M–O bond distances reported by Villaneau and co-workers in their series of [M(Mo_5_O_13_(OMe)_4_NO)_2_]^*X*−^ (M = Ba^2+^, Sr^2+^, Ca^2+^, Bi^3+^, Ce^3+^, and Eu^3+^) compounds (2.45–2.76 Å).^35^ The smaller Zr^4+^ and Hf^4+^ cations, with effective ionic radii of 0.84 Å and 0.83 Å respectively, are significantly more Lewis acidic than the M^2+^/M^3+^ cations previously discussed (which have effect ionic radii of 1.07–1.42 Å) and consequently form stronger bonding interactions with the [Mo_5_O_13_(OMe)_4_NO]^3−^ units.^42^

**Fig. 4:**
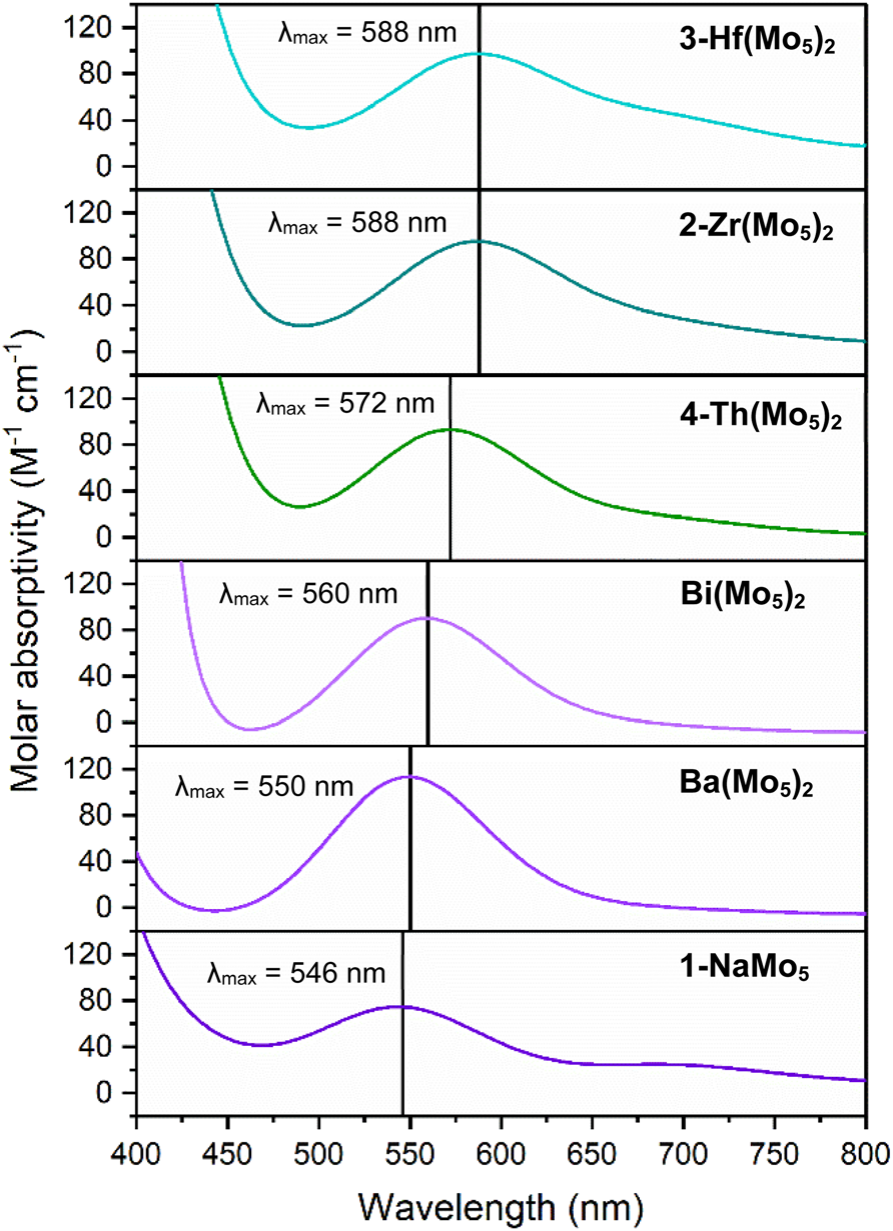
Electronic absorption spectra of 1-NaMo_5_, 2-Zr(Mo_5_)_2_, 3-Hf(Mo_5_)_2_, 4-Th(Mo_5_)_2_, and the Ba/Bi containing complexes previously reported by Villanneau (ref. 35). Data collected in MeCN at 21 °C.

Along with the observed changes in bond metrics upon incorporation of M^4+^ cations into the sandwich complex, there was also an observed change in color. Solutions of 1-NaMo_5_ and the M^2+^/M^3+^ containing sandwich complexes are all reported to be pale purple.^30,35^ This contrasts the M^4+^ derivatives, where solutions of 2-Zr(Mo_5_)_2_ and 3-Hf(Mo_5_)_2_ appear blue/green. This observation was investigated using electronic absorption spectroscopy (Fig. 4). The spectra show that the color of 1-NaMo_5_, the M^2+^/M^3+^ sandwich complexes, 2-Zr(Mo_5_)_2_, and 3-Hf(Mo_5_)_2_ can be attributed to a weak absorption at 500–600 nm. This band was previously assigned to the d_*xy*_ ← d_*xz*_,d_*yz*_ transition within the Mo^II^(NO) unit and appears to increase in wavelength as the Lewis acidity of the heterometal increases (with positive charge increasing and ionic radius decreasing across the series).^30^

After development of the synthetic methodology for transition metal derivatives, attention was turned to the incorporation of actinide(iv) centers into the framework. Extension to Th was accomplished by treating a purple solution 1-NaMo_5_ in MeOH with approximately half an equivalent of ThCl_4_·2DME. As was the case for the Zr and Hf reactions, a precipitate (grey/green in color) formed immediately upon mixing and was isolated by filtration and extraction with DCM. Analysis of the isolated product by ^1^H NMR spectroscopy (Fig. 2, 4-Th(Mo_5_)_2_) gave an almost identical spectrum to those obtained for 2-Zr(Mo_5_)_2_ and 3-Hf(Mo_5_)_2_, indicating that incorporation of the larger (diamagnetic) Th^4+^ cation leads to almost no change in the chemical environments of both the protons of the bridging –OMe groups and the TBA^+^ cations. The minor difference in color between MeCN solutions of 4-Th(Mo_5_)_2_ and 2-Zr(Mo_5_)_2_/3-Hf(Mo_5_)_2_ was investigated by UV-vis spectroscopy (Fig. 3). A slight shift in the position of the absorption maximum was observed, occurring at 572 nm for 4-Th(Mo_5_)_2_ (compared to 588 nm for 2-Zr(Mo_5_)_2_ and 3-Hf(Mo_5_)_2_). This places the *λ*_max_ of 4-Th(Mo_5_)_2_ in between the M^2+^/M^3+^ containing sandwich complexes (*λ*_max_ = 530–560 nm) and the other M^4+^ analogues.^35^ This could be attributed to the intermediate Lewis acidity of Th^4+^, which possesses a higher charge than the M^2+^/M^3+^ cations but has a larger ionic radius than Zr^4+^ and Hf^4+^ (reducing charge density). These studies show how manipulation of central metal cation can be used to “fine tune” the energy of d–d transitions within the molybdenum framework.

Encouraged by the results obtained from the reaction of 1-NaMo_5_ with ThCl_4_·2DME, the same reaction was attempted with UCl_4_. Approximately half an equivalent of the uranium precursor was first dissolved in MeOH, forming a pale green solution, and was then added slowly to one equivalent of a purple solution of 1-NaMo_5_. This led to the immediate formation of a brown precipitate. Though the observed color was very different to the reactions described above, formation of a precipitate was a promising sign. The solid was isolated and was subsequently analyzed by ^1^H NMR spectroscopy. The obtained spectrum (Fig. 2, 5-U(Mo_5_)_2_) was strikingly different, featuring four peaks between −2 and −5 ppm, assigned to TBA^+^, and an additional peak at 9.58 ppm, assigned to the bridging alkoxide groups of [Mo_5_O_13_(OMe)_4_NO]^3−^. It is postulated that incorporation of the paramagnetic U^4+^ cation into the sandwich complex (which already contains two Mo^2+^ centers)^30,35^ leads to significant paramagnetic shifting of the peaks associated with both the alkoxide groups and the counter ions.

**Fig. 5:**
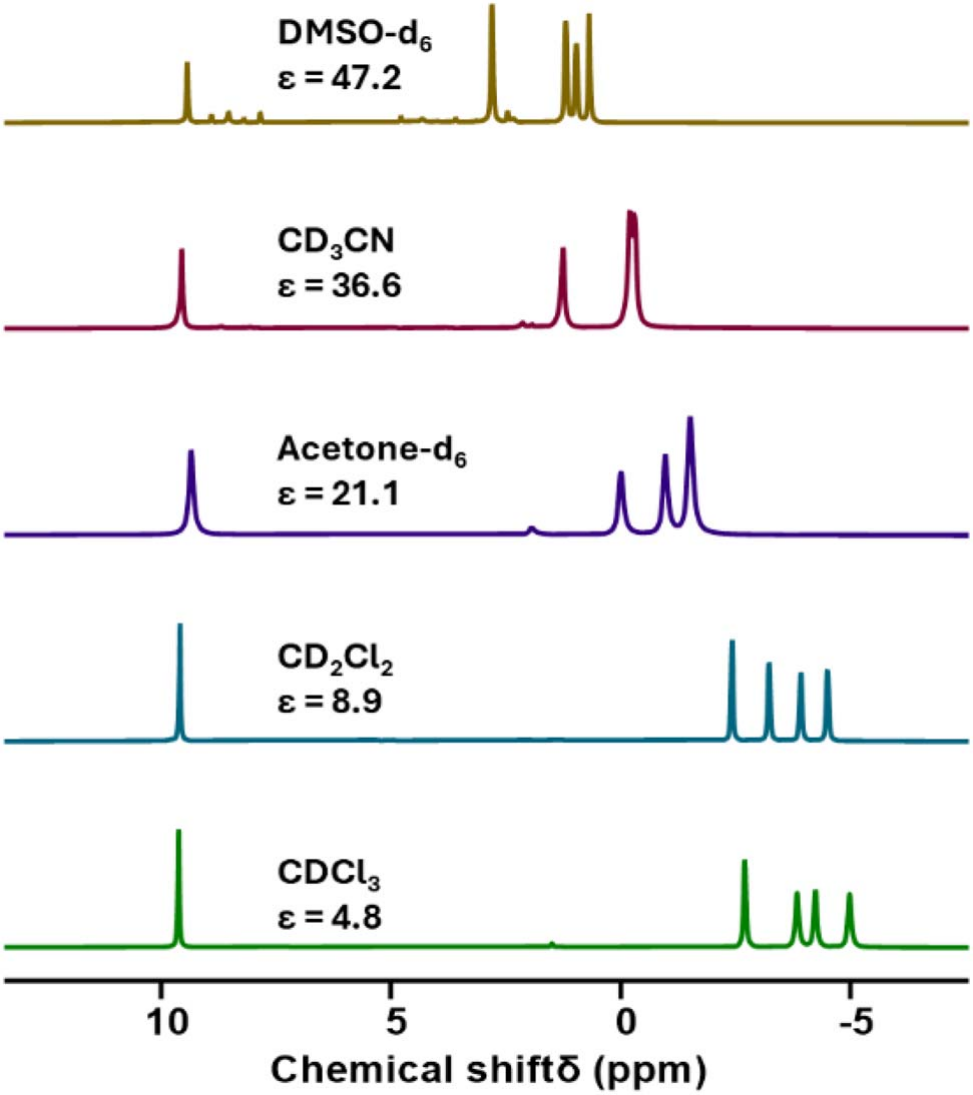
A series of ^1^H NMR spectra recorded on samples of 5-U(Mo_5_)_2_ dissolved in a range of deuterated solvents with varying dielectric constants. Values for dielectric constants were taken from ref. 37.

To investigate this, the ^1^H NMR spectrum was reacquired in a range of solvents with varying dielectric constants (Fig. 5).^43^ From this we observe that the chemical shifts of the peaks associated with the TBA cations varied with the dielectric constant of the solvent, with more significant deviations from the “standard” chemical shifts of TBA^+^ (*i.e.* 1–3.5 ppm) observed in solvents with lower dielectric constant. Conversely, the chemical shift of the peak assigned to the alkoxide groups remained essentially constant. These findings are consistent with the formation of (TBA)_2_[U{Mo_5_O_13_(OMe)_4_NO}_2_] and show that close association of the TBA cations to the paramagnetic metal cluster in low dielectric constant solvents causes the large deviations in the observed ^1^H NMR chemical shifts, with this effect lessening as solvent polarity is increased (presumably as the ion-pair are separated by solvation). This effect was further investigated using ^1^H DOSY NMR spectroscopy. When 5-U(Mo_5_)_2_ was dissolved in CD_3_CN (the most polar solvent 5-U(Mo_5_)_2_ was found to be stable in for extended periods), significantly different diffusion coefficients of 1.26 × 10^−9^ m^2^ s^−1^ (average value from the four peaks assigned to the TBA cations) and 7.91 × 10^−10^ m^2^ s^−1^ were obtained for the TBA cations and the anionic {U(Mo_5_)_2_} cluster respectively. This is consistent with the ions existing as separate, fully solvated, species in solution. Conversely, when 5-U(Mo_5_)_2_ was dissolved in CDCl_3_ (a lower polarity solvent), the obtained diffusion coefficients for the TBA cations and the anionic {U(Mo_5_)_2_} cluster were the same (within error) at *ca.* 9.1 × 10^−10^ m^2^ s^−1^ (exact values given in ESI Fig. S13†). This supports the hypothesis that these species are tightly ion paired in solution and helps to explain the large magnetic interaction between the paramagnetic cluster and the associated cations in solvents with low dielectric constants.

**Fig. 6:**
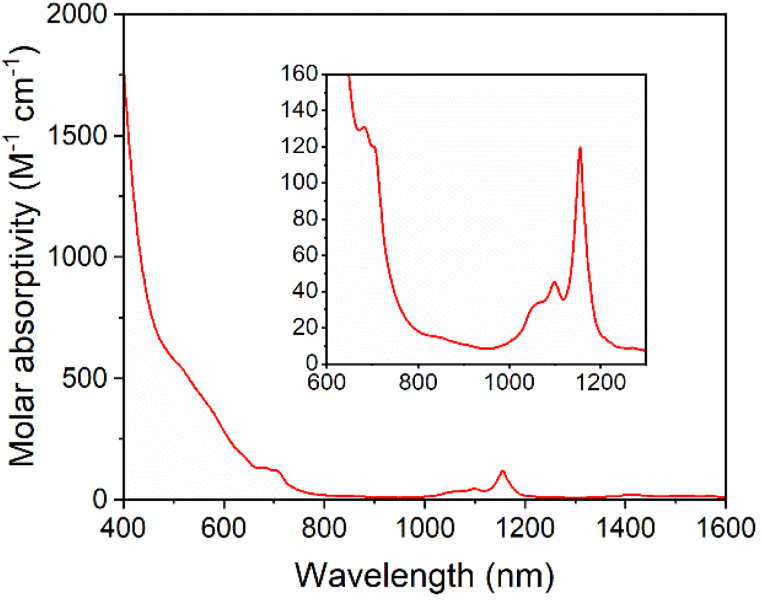
UV-vis/NIR spectrum of 5-U(Mo_5_)_2_ collected in MeCN at 21 °C, with the region between 600 and 1300 nm expanded to highlight the f–f transitions (inset).

Solutions of 5-U(Mo_5_)_2_ appeared brown and much darker than solutions of the other sandwich complexes synthesized. This phenomenon was investigated by electronic absorption spectroscopy. The UV-vis/NIR spectrum obtained for 5-U(Mo_5_)_2_ (Fig. 6) is very different, with very broad and intense absorption observed across most of the visible region (*i.e.* 400–700 nm). The few examples of “U-POM” complexes for which electronic absorption data is discussed also show very broad absorptions in the visible region, which is attributed to charge transfer between U^4+^ and W^6+^.^8,18,44^ It therefore is reasonable to suggest uranium–molybdenum charge transfer is responsible for the broad absorbance in the visible region for 5-U(Mo_5_)_2_. The shoulder observed at around 685 nm (*ε* = 197 mol^−1^ dm^3^ cm^−1^) is characteristic of a U^4+^ f–f transition.^8,44,45^ Analysis of the NIR region (Fig. 6) shows two sharp bands at 1100 nm and 1156 nm, with molar extinction coefficients of *ε* = 66 mol^−1^ dm^3^ cm^−1^ and *ε* = 172 mol^−1^ dm^3^ cm^−1^ respectively. These are also assigned to U^4+^ f–f transitions, and line up closely to previously reported values for [U(P_2_W_17_O_61_)_2_]^16−^.^8^

**Fig. 7:**
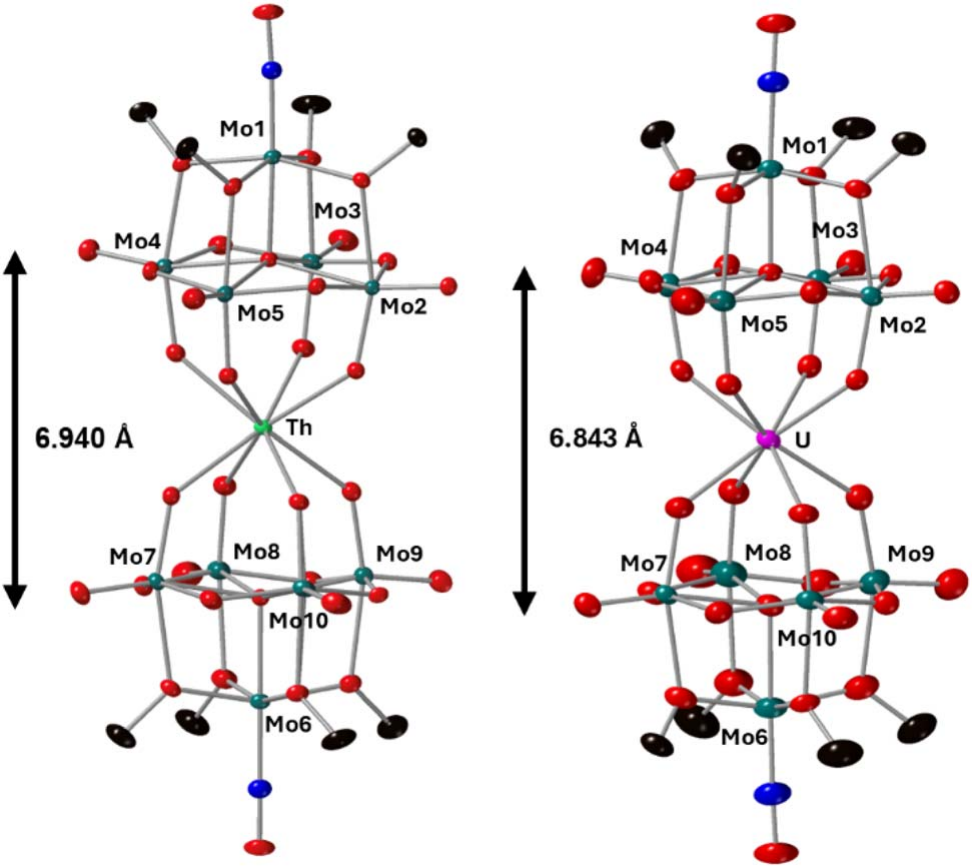
Single crystal X-ray diffraction structures of 4-Th(Mo_5_)_2_ and 5-U(Mo_5_)_2_ with probability ellipsoids set at 50%. The tetrabutylammonium cations and some disorder has been masked for clarity. Detailed bond length data is given in the ESI.†

Complexes 4-Th(Mo_5_)_2_ and 5-U(Mo_5_)_2_ were characterized by single crystal X-ray diffraction (Fig. 7). Crystals were grown by vapor diffusion of Et_2_O into saturated solutions of the product dissolved in MeCN. In comparison to their Zr- and Hf-congeners, 4-Th(Mo_5_)_2_ and 5-U(Mo_5_)_2_ feature much larger Th^4+^ and U^4+^ cations which have effective ionic radii of 1.05 Å and 1.00 Å respectively.^42^ This leads to increased M–O bond distances of *ca.* 2.41 Å for 4-Th(Mo_5_)_2_ and 2.36 Å for 5-U(Mo_5_)_2_. These distances, and the square-antiprismatic geometry, are in line with other examples of [An(POM)_2_]^*X*−^ sandwich complexes present in the literature, which feature a number of different polyoxometalate clusters acting as ligands (including [W_5_O_18_]^6−^, [GeW_11_O_39_]^7−^, [PMo_11_O_39_]^7−^, and [P_2_W_17_O_61_]^10−^) and were all synthesized in water.^7,16,19,46^ It is therefore apparent that the nature of the POM ligand, the overall charge of the sandwich complex, and solvent used during synthesis have no significant impact on local coordination environment of the actinide center. A consequence of the incorporation of Th^4+^/U^4+^ compared to Zr^4+^/Hf^4+^ is that the distance between the two [Mo_5_O_13_(OMe)_4_NO]^3−^ units of the sandwich complex increases. This can be conveniently quantified by examining the distance between the central μ_5_-oxo in each cluster (shown in Fig. 7). This distance is 6.940 Å for 4-Th(Mo_5_)_2_ and 6.843 Å for 5-U(Mo_5_)_2_, compared to 6.652 Å for 2-Zr(Mo_5_)_2_ and 6.643 Å for 3-Hf(Mo_5_)_2_. These observations show how differences in the charge and size of the central metal ion lead to changes in both the local co-ordination geometry and the extended structure.

4-Th(Mo_5_)_2_ and 5-U(Mo_5_)_2_ represent rare examples of An^4+^-poloyoxomolybdate complexes, with far fewer known than for their tungsten based relatives. There is only one other An^4+^(POM)_2_ sandwich complex which utilizes molybdenum as a framework metal. [Th(PMo_11_O_39_)]^10−^ was characterized by single crystal X-ray diffraction in 2008 by Copping and co-workers and features an identical average Th–O bond length (2.41 Å) as 4-Th(Mo_5_)_2_.^46^ A handful of other “non-sandwich complex” examples are known, including a U^4+^ containing phosphomolybdate ([NaU(Mo_6_P_4_O_31_H_7_)_2_]·5Na·(H_2_O)_*n*_) and some mixed-metal molybdenum blues (*e.g.* [Mo_72_U_8_O_216_(OH)_24_(H_2_O)_68_·24(H_2_O)]). The majority of polyoxomolybdate clusters which feature a An^4+^ center are based on An^4+^ centered Silverton-type clusters, with the general formula [AnMo_12_O_42_]^8−^.^23,47–50^ Both Th^4+^ and U^4+^ centered clusters are known, with the bulk of the work in this area focusing on the binding of metal cations (including Ni^2+^, Fe^3+^, VO^2+^, Er^3+^ and Th^4+^) to the surface of the polyoxomolybdate. Electrochemical studies on [ThMo_12_O_42_]^8−^ and [UMo_12_O_42_]^8−^ showed a lack of reversible cluster reduction, common for “type II” POMs in which the framework metal has a *cis*-dioxo environment. However, a reversible one-electron oxidation was observed for [UMo_12_O_42_]^8−^ at 0.9 V *vs.* SCE at pH = 0.^26,51^ Attempts were made to isolate the oxidized U^5+^ species by controlled potential electrolysis, with a color change from yellow to orange observed.^26^ The electronic absorption spectrum was recorded but isolation of the product was unsuccessful, with the color of both solutions and precipitated solid fading after a few hours.

**Fig. 8:**
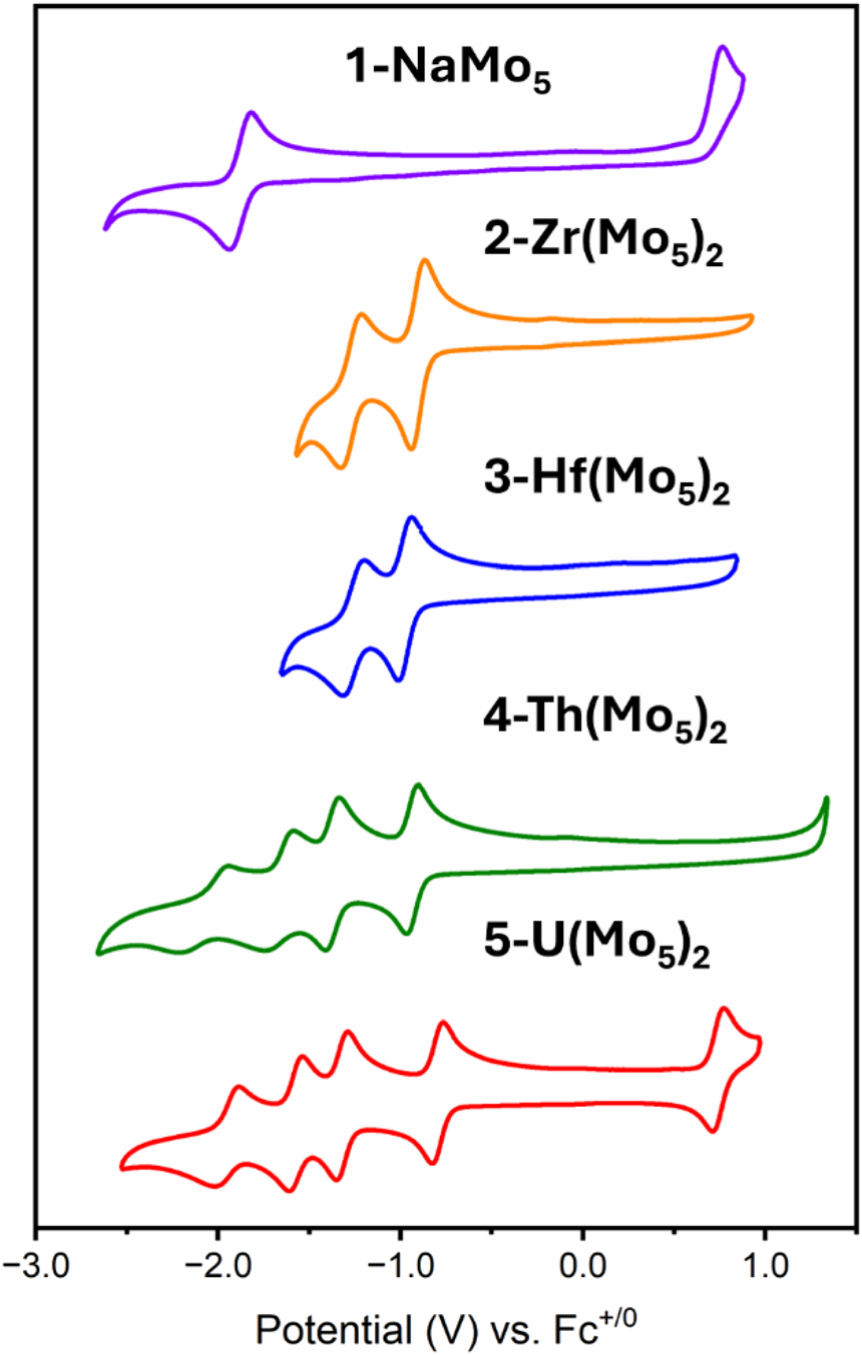
Cyclic voltammograms of 1-NaMo_5_, 2-Zr(Mo_5_)_2_, 3-Hf(Mo_5_)_2_, 4-Th(Mo_5_)_2_, and 5-U(Mo_5_)_2_. The data was acquired in MeCN with 0.1 M TBA(PF_6_) supporting electrolyte, 1 mM of cluster, and a scan rate of 200 mV s^−1^.

The electrochemical properties of the series of sandwich complexes synthesized in this work, and 1-NaMo_5_, were probed using cyclic voltammetry (CV). The results, presented in Fig. 8, show major differences between the compounds. The electrochemistry of 1-NaMo_5_ was previously discussed by Proust.^30^ No evidence of reduction before −1.5 V *vs.* SCE (in MeCN with a Pt working electrode) was observed, and an irreversible oxidation event at 1.09 V *vs.* SCE was assigned to the Mo(NO)^3+^ group. The oxidation of 1-NaMo_5_ is shown in Fig. 8 at 0.76 *vs.* Fc/Fc^+^ but it is also accompanied by a reversible reduction at −1.88 V. Given the difference in the position of the oxidation event in this study compared to Proust's original work (approx. 0.33 V), the reduction event of 1-NaMo_5_ would be expected to occur at roughly −1.55 V *vs.* SCE and therefore likely fell outside the scope of their original study. No discussion of the electrochemical properties of the sandwich complexes containing M^2+^/M^3+^ cations was given by Villaneau and co-workers in their work.^35^ To give context to the current study, both (TBA)_4_[Ba{Mo_5_O_13_(OMe)_4_NO}_2_] and (TBA)_3_[Bi{Mo_5_O_13_(OMe)_4_NO}_2_] were synthesized and the CV's were recorded (Fig. S32 and S33†). No evidence of any reversible redox reduction was found, indicating that sandwich complexes containing M^2+^ or M^3+^ cations are not stable in their reduced forms.

Contrary to this, CVs of 2-Zr(Mo_5_)_2_ and 3-Hf(Mo_5_)_2_ (Fig. 8) show that these M^4+^ containing sandwich complexes can reversibly accept up to two electrons. The enhanced stability of the reduced form of these sandwich complexes can be attributed to lower overall charge and the ability of the M^4+^ cation to screen charge repulsion between the two halves of the complex. This represents an improvement in the redox properties of these systems compared to the M^2+^/M^3+^ containing analogues; however, upon exposure to more reducing potentials (less than approx. −1.5 V *vs.* Fc/Fc^+^), broad irreversible peaks are observed (Fig. S34 and S35†). This indicates that further reduction of 2-Zr(Mo_5_)_2_ and 3-Hf(Mo_5_)_2_ leads to structural rearrangement or decomposition.

The limited reductive stability of the group(iv) sandwich complexes can be overcome through the incorporation of An^4+^ cations into the framework. The CVs of 4-Th(Mo_5_)_2_ and 5-U(Mo_5_)_2_ (Fig. 8) possess a series of four reversible reduction events between −0.5 and −2.5 V, presumably corresponding to the addition of up to two electrons to each {Mo_5_} unit. The ability to add up to four electrons to 4-Th(Mo_5_)_2_ and 5-U(Mo_5_)_2_ without any evidence of instability is a drastic enhancement in redox behavior compared to the previously obtained M^2+^/M^3+^ (and to a lesser extent Zr^4+^/Hf^4+^) containing complexes. This can be rationalized by considering that the An^4+^ cations can reduce the overall charge of the system, like Zr^4+^ and Hf^4+^, but the larger actinides also spatially separate the negatively charged {Mo_5_} units of the sandwich complex more than the smaller (harder) transition metal cations (Fig. 3 and 7). This serves to increase the stability of the reduced complex by minimizing intramolecular repulsion.

An additional redox event is observed for 5-U(Mo_5_)_2_ at 0.74 V *vs.* Fc/Fc^+^, which is attributed to the oxidation of U(iv) to U(v). Reported values for U(v)/U(iv) redox couples vary drastically, occurring anywhere from 1.09 to −2.70 V *vs.* Fc/Fc^+^.^24^ The extreme variation is undoubtedly a result of huge structural differences and the range of solvents used in electrochemical studies, many of which can directly coordinate to the uranium center.^24^ In order to assess the solvent dependence of the U(v)/U(iv) redox couple of 5-U(Mo_5_)_2_, the CV was also recorded in DCM and THF, with the oxidation event observed at 0.64 V and 0.65 V respectively. The fairly minor variation in the position of the redox couple is evidence of the steric protection provided by the {Mo_5_} ligands (limiting the access of solvent molecules to the U center) and the retention of the An(Mo_5_)_2_ structure in solution.

**Fig. 9:**
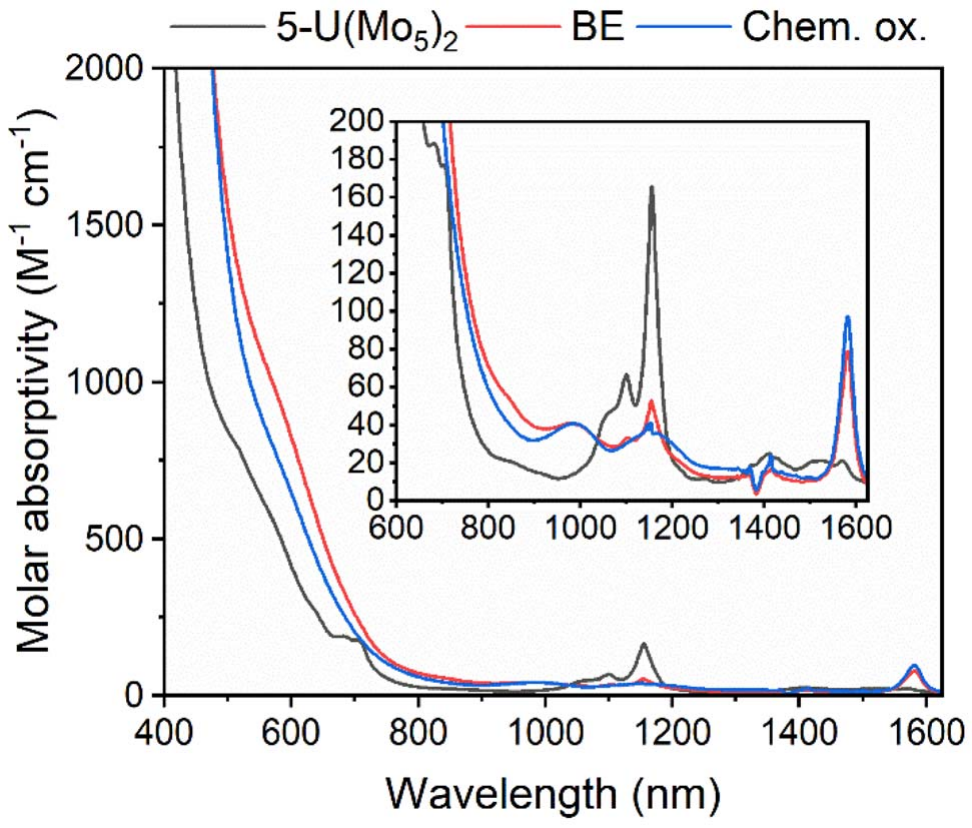
UV-vis/NIR spectra of pristine 5-U(Mo_5_)_2_ (black), after bulk electrolysis of 5-U(Mo_5_)_2_ at 0.78 V *vs.* Fc/Fc^+^ (red, BE), and after treatment of 5-U(Mo_5_)_2_ with an excess of [NO][PF_6_] (blue, Chem. ox.). The region between 600 and 1600 nm is expanded to highlight the f–f transitions. All spectra were recorded in DCM.

The observation of a reversible U(v)/U(iv) redox couple in the CV of 5-U(Mo_5_)_2_ encouraged us to further investigate the stability of the oxidized species by bulk electrolysis. Initially, chronoamperometry was performed in MeCN, however this quickly led to loss of color from the solution and precipitation of a dark brown solid. This made post-electrolysis characterization (*e.g.* by cyclic voltammetry) problematic and therefore alternative solvents were investigated. Controlled potential bulk electrolysis of a 1 mM solution of 5-U(Mo_5_)_2_ in DCM (0.1 M TBAPF_6_ supporting electrolyte) at 0.78 V *vs.* Fc/Fc^+^ appeared more promising, with a brown color persisting throughout the experiment. The amount of charge passed during the experiment was consistent with the loss of one electron per molecule of 5-U(Mo_5_)_2_. Formation of the oxidized U^5+^ species was interrogated by CV and UV-vis/NIR spectroscopy. The CV obtained after electrolysis was almost identical to that of pristine 5-U(Mo_5_)_2_ (showing the sandwich complex remaining intact during electrolysis), however the open circuit potential (OCP) changed from −0.87 V pre-electrolysis to 0.68 V post-electrolysis (both *vs.* Fc/Fc^+^) (Fig. S39†). The fact that the OCP is above the potential of the U(v)/U(iv) redox couple is indicative of successful formation of the U^5+^ species. UV-vis/NIR spectroscopy (Fig. 9, red line) revealed the presence of new absorption bands at approximately 980 nm and 1582 nm, along with a significant decrease in intensity of the bands around 1100 nm (the shoulder at 682 nm is also no longer resolved). The new feature at 1582 nm is caused by a characteristic U^5+^ f–f transition, with very similar absorption bands observed in the spectra of numerous isolated U^5+^ complexes and [U^5+^(PW_11_O_39_)_2_]^9−^, generated by bulk electrolysis in MeCN (though not isolated).^26,52,53^ The persistence of the peaks at 1100 nm and 1156 nm likely indicate less than quantitative conversion of U^4+^ → U^5+^.

To gain further evidence of the formation of the oxidized U^5+^ containing species, chemical oxidation was pursued. Given the low solubility of the oxidized product in MeCN observed during electrochemical studies, it was reasoned that chemical oxidation of 5-U(Mo_5_)_2_ in MeCN would lead to precipitation of the oxidized species and allow for simple isolation of the product. With this in mind, 5-U(Mo_5_)_2_ was treated with an excess of [NO][PF_6_] (oxidation potential of 0.87 V *vs.* Fc/Fc^+^) in MeCN. This led to the immediate precipitation of a black/brown solid, which was separated by filtration and extracted into DCM. This solution was characterized by UV-vis/NIR spectroscopy (Fig. 9, blue line) and showed the same absorption bands at 980 nm and 1582 nm that were observed after bulk electrolysis of 5-U(Mo_5_)_2_, as well as a broad absorbance around 1160 nm which is more readily resolved as the absorption bands for 5-U(Mo_5_)_2_, located between 1100–1150 nm are essentially gone. The spectrum is consistent with the results obtained from oxidation by bulk electrolysis and, together with the absence of any major absorption bands characteristic of 5-U(Mo_5_)_2_, supports the formation of (TBA)[U^5+^{Mo_5_O_13_(OMe)_4_NO}_2_] (6-U(Mo_5_)_2_). Crude 6-U(Mo_5_)_2_ obtained by chemical oxidation was further characterized by CV and ^1^H NMR spectroscopy (Fig. S14 and S40†). The obtained CV was very similar to that of 5-U(Mo_5_)_2_ with the only significant difference being the OCP, which was shifted to 0.66 V after reaction with [NO][PF_6_] compared to −0.87 V for 5-U(Mo_5_)_2_. This observation mirrors the results obtained from controlled potential electrolysis. The ^1^H NMR featured four peaks at 0.5–2.5 ppm, assigned to the TBA cation, and a large peak at 4.76 ppm assigned to the –OMe groups of 6-U(Mo_5_)_2_. The spectrum differs significantly from that of 5-U(Mo_5_)_2_, showing that the change in electronic configuration at the uranium center upon oxidation of U^4+^ → U^5+^ results in large changes in the local environment of the protons in the system. Integration of the peaks supports the presence of one TBA^+^ per cluster.

**Fig. 10:**
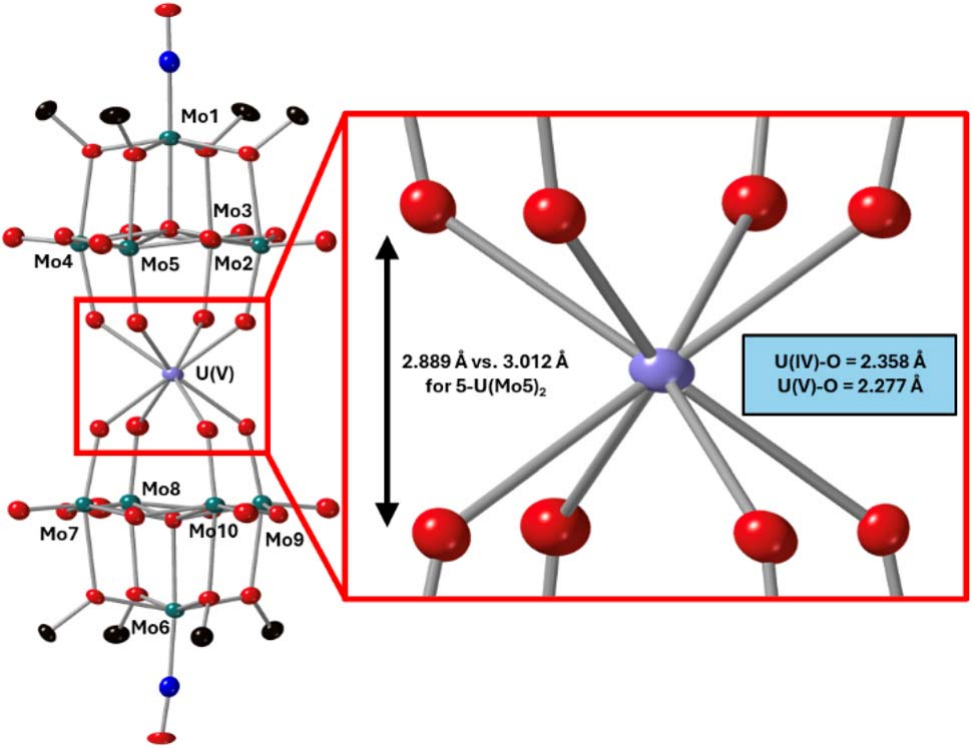
Single crystal X-ray diffraction structure of 6-U(Mo_5_)_2_ with probability ellipsoids set at 50%. The tetrabutylammonium cation and some disorder has been masked for clarity. Detailed bond length data is given in the ESI.†

After characterization of the crude material, single crystals of 6-U(Mo_5_)_2_ were grown. Vapor diffusion of pentane into a saturated solution of the cluster dissolved in DCM at −40 °C led to the formation of brown crystals which were analyzed by SCXRD. The structure confirms the successful isolation of (TBA)[U{Mo_5_O_13_(OMe)_4_NO}_2_] (6-U(Mo_5_)_2_), the first example of a U^5+^ containing “U(POM)_2_” sandwich complex characterized by X-ray diffraction. Bond length analysis shows only minor variations in bond distances within each of the [Mo_5_O_13_(OMe)_4_NO]^3−^ “ligands” compared to the series of M^4+^ centered sandwich complexes presented in this work. Comparison of the structure of 6-U(Mo_5_)_2_ to 5-U(Mo_5_)_2_ expectedly shows differences in the local co-ordination environment of uranium (shown in Fig. 10). The average U–O bond length is *ca.* 2.28 Å for 6-U(Mo_5_)_2_ compared to 2.36 Å for 5-U(Mo_5_)_2_. This is consistent with the expected decrease in the ionic radius of the uranium center upon oxidation, though there is no reported ionic radius for eight coordinate U^5+^. The diffraction data obtained in this study was used to calculate the effective ionic radius for the eight-co-ordinate U^5+^ present in 6-U(Mo_5_)_2_. An average value of 0.93 Å was obtained from two methods of calculation (details given in the ESI†). This lies in between with the reported values of eight coordinate U^4+^ (1.00 Å) and eight coordinate U^6+^ (0.86 Å). It is also expectedly larger than the reported value for seven co-ordinate U^5+^ (0.84 Å). The decrease in ionic radius and U–O bond length compared to 5-U(Mo_5_)_2_ propagates changes to the overall structure, with the O–O distance (Fig. 10) decreasing by *ca.* 0.12 Å and the distance between the central μ_6_-oxo of each {Mo_5_} unit dropping by *ca.* 0.09 Å. These distances place the structure at an intermediate position between the Tm^4+^ structures (2-Zr(Mo_5_)_2_ and 3-Hf(Mo_5_)_2_) and the An^4+^ (4-Th(Mo_5_)_2_ and 5-U(Mo_5_)_2_), exemplifying the unique ability of actinide cations to maintain longer M–O bonds than transition metals even when placed in a higher oxidation state.

The stability of 6-U(Mo_5_)_2_ was probed by electronic absorption spectroscopy. The UV-vis/NIR spectrum of a solution of 6-U(Mo_5_)_2_ dissolved in DCM (Fig. S32†) was monitored over a period of five days. The formation of two new peaks, at 680 nm and 830 nm respectively, was observed, which gradually grew larger over the course of the experiment. These peaks may be caused by the formation of decomposition products, however, there was no notable decrease in the intensity of the peaks at 980 nm, 1160 nm, and 1582 nm, assigned to 6-U(Mo_5_)_2_. The lack of any major loss of intensity from these bands suggests minimal decomposition of 6-U(Mo_5_)_2_ over this period and represents a drastic increase in stability compared to any of the U(v)-POM complexes previously observed during electrochemical oxidation studies.^25,26^ This can be attributed to the steric protection offered by the [Mo_5_O_13_(OMe)_4_NO]^3−^, as well as the use of dry organic solvents during synthesis and characterization, with the absence of water likely being a key factor in preventing decomposition *via* the formation of the stable [UO_2_]^+^ fragment, followed by disproportion and demetallation.

## Conclusion

In conclusion, we report the synthesis and characterization of a new series of polyoxomolybdate-alkoxide sandwich complexes. The series includes both thorium and uranium containing derivatives, which represent the first examples of actinide-polyoxomolybdate sandwich complexes to be reported outside of water. A thorough examination of the electrochemical properties of the series has shown how incorporation of M^4+^ cations into the molybdenum framework can enhance the redox properties of the “ligand” compared to the previously isolated M^2+^/M^3+^ containing derivatives, with this effect maximized for the An^4+^ containing compounds. This observation supports the possibility of actinide doping as a method of enhancing the redox properties of polyoxometalates, offering new utility for these systems beyond being a robust system for inspection of the An coordination environment. Observation of a reversible U(v)/U(iv) redox couple in the CV of 5-U(Mo_5_)_2_ led us to probe the oxidative stability of this cluster. Both electrochemical and chemical oxidation studies were carried out, with results showing the ability to access the oxidized U(v) containing cluster (6-U(Mo_5_)_2_), which was characterized by ^1^H NMR spectroscopy, UV-vis/NIR spectroscopy, CV and SCXRD. This represents the first example of a “U(v)-POM” complex which has been isolated and fully characterized (with previous studies only able to generate U(v) species in solution and characterize by UV-vis/NIR). 6-U(Mo_5_)_2_ was found to be relatively stable, with solutions of the cluster dissolved in DCM only undergoing slow decomposition over several days at room temperature. This is likely a result of the ability to exclude water from the preparation and characterization of this cluster, which prohibits decomposition *via* the formation of the stable [UO_2_]^*n*+^ unit. These results unlock exciting opportunities for detailed characterization of this U(v) containing cluster, with magnetic measurements and electron paramagnetic resonance spectroscopy studies currently underway.

## Data availability

The data supporting this article have been included as part of the ESI.† Crystallographic data for 2-Zr(Mo_5_)_2_, 3-Hf(Mo_5_)_2_, 4-Th(Mo_5_)_2_, 5-U(Mo_5_)_2_, and 6-U(Mo_5_)_2_ has been deposited at the CCDC under 2346524–2346527, and 2349690.

## Author contributions

D. S. conceived of the project and synthesized and characterized all compounds. W. W. B. determined the crystal structures of 2-Zr(Mo_5_)_2_, 3-Hf(Mo_5_)_2_, and 6-U(Mo_5_)_2_; M. R. C. determined the crystal structures of 4-Th(Mo_5_)_2_ and 5-U(Mo_5_)_2_. E. M. M. directed the project. The manuscript was written through contributions of all authors. All authors have given approval to the final version of the manuscript.

## Conflicts of interest

The authors declare no competing financial interest.

## Supplementary Material

SC-015-D4SC02644F-s001

SC-015-D4SC02644F-s002
