# Supplementary material for: Leveraging a reduced polyoxomolybdate-alkoxide cluster for the formation of a stable U(v) sandwich complex
Source: Chem Sci. 2024 Jun 17;15(28):11072–83. doi: 10.1039/d4sc02644f (PMC11253122; doi:10.1039/d4sc02644f)
Supplement: SC-015-D4SC02644F-s001 [file SC-015-D4SC02644F-s001.pdf]

Electronic Supplementary Material (ESI) for Chemical Science.
This journal is © The Royal Society of Chemistry 2024

Electronic Supporting Information

Leveraging a reduced polyoxomolybdate-alkoxide cluster for the formation of a stable U(V) sandwich complex

Dominic Shiels[1]*, William W. Brennessel[1], Matthew R. Crawley[2], and Ellen M. Matson[1]*

[1] *Department of Chemistry, University of Rochester, Rochester NY, 14627 USA*

[2] *Department of Chemistry, University at Buffalo, The State University of New York, Buffalo NY 14620, USA.*

Corresponding Author Contact Information:

Dominic Shiels: dshiels@ur.rochester.edu

Ellen M. Matson: matson@chem.rochester.edu

Contents

1. ^{1}H NMR spectra **S4**

Figure S1: ^{1}H NMR spectrum (400 MHz) of $(TBA)_2[Mo_5O_{13}(OMe)_4NO][Na(MeOH)]$ (**1-NaMo$_5$**) in CD_3CN. The peak marked with an asterisk corresponds to MeOH. S4

Figure S2: ^{1}H NMR spectrum (500 MHz) of $(TBA)_4[Ba\{Mo_5O_{13}(OMe)_4NO\}_2]$ in CD_3CN. Peaks marked with an asterisk correspond to MeOH. S5

Figure S3: ^{1}H NMR spectrum (500 MHz) of $(TBA)_3[Bi\{Mo_5O_{13}(OMe)_4NO\}_2]$ in CD_3CN....... S6

Figure S4: ^{1}H NMR spectrum (500 MHz) of $(TBA)_2[Zr\{Mo_5O_{13}(OMe)_4NO\}_2]$ (**2-Zr(Mo$_5$)$_2$**) in CD_2Cl_2........................ S7

Figure S5: ^{1}H NMR spectrum (500 MHz) of $(TBA)_2[Hf\{Mo_5O_{13}(OMe)_4NO\}_2]$ (**3-Hf(Mo$_5$)$_2$**) in CD_2Cl_2........................ S8

Figure S6: ^{1}H NMR spectrum (500 MHz) of $(TBA)_2[Th\{Mo_5O_{13}(OMe)_4NO\}_2]$ (**4-Th(Mo$_5$)$_2$**) in CD_2Cl_2........................ S9

Figure S7: ^{1}H NMR spectrum (500 MHz) of $(TBA)_2[U\{Mo_5O_{13}(OMe)_4NO\}_2]$ (**5-U(Mo$_5$)$_2$**) in CD_2Cl_2. Peaks marked with asterisks correspond to toluene impurity. S10

Figure S8: ^{1}H NMR spectrum (500 MHz) of $(TBA)_2[U\{Mo_5O_{13}(OMe)_4NO\}_2]$ (**5-U(Mo$_5$)$_2$**) in $CDCl_3$........................ S11

Figure S9: ^{1}H NMR spectrum (500 MHz) of $(TBA)_2[U\{Mo_5O_{13}(OMe)_4NO\}_2]$ (**5-U(Mo$_5$)$_2$**) in acetone-d_6. .... S12

Figure S10: ^{1}H NMR spectrum (500 MHz) of $(TBA)_2[U\{Mo_5O_{13}(OMe)_4NO\}_2]$ (**5-U(Mo$_5$)$_2$**) in CD_3CN. S13

Figure S11: ^{1}H NMR spectrum (500 MHz) of $(TBA)_2[U\{Mo_5O_{13}(OMe)_4NO\}_2]$ (**5-U(Mo$_5$)$_2$**) in DMSO-d_6........................ S14

Figure S12: ^{1}H DOSY NMR spectrum (500 MHz) of a 2mM solution $(TBA)_2[U\{Mo_5O_{13}(OMe)_4NO\}_2]$ (**5-U(Mo$_5$)$_2$**) in $CDCl_3$. The obtained diffusion coefficient (D in m^2/s) for each peak in the ^{1}H NMR spectrum is given with the standard deviation (σ-D). The peaks at −2.723, −3.915, and −4.188 ppm can be assigned to the TBA cation, while the peak at 9.663 ppm is assigned to $\{U(Mo_5)_2\}$ cluster. S15

Figure S13: ^{1}H DOSY NMR spectrum (500 MHz) of a 2 mM solution of $(TBA)_2[U\{Mo_5O_{13}(OMe)_4NO\}_2]$ (**5-U(Mo$_5$)$_2$**) in CD_3CN. The obtained diffusion coefficient (D in m^2/s) for each peak in the ^{1}H NMR spectrum is given with the standard deviation (σ-D). The peaks at 0.093, 0.195, 1.251 and 1.696 ppm can be assigned to the TBA cation, while the peak at 9.519 ppm is assigned to $\{U(Mo_5)_2\}$ cluster. S16

Figure S14: ^{1}H NMR spectrum (500 MHz) of crude $(TBA)[U\{Mo_5O_{13}(OMe)_4NO\}_2]$ (**6-U(Mo$_5$)$_2$**), obtained from oxidation of $(TBA)_2[U\{Mo_5O_{13}(OMe)_4NO\}_2]$ (**5-U(Mo$_5$)$_2$**) with an excess of

[NO][PF_6]. Spectrum recorded in CD_2Cl_2. The peak marked with an asterisk corresponds to the -OMe groups of **5-U(Mo_5)$_2$**. S17

2. Electronic absorption spectra **S18**

Figure S15: UV-Vis spectra of 1 mM, 2 mM, 3 mM, and 4 mM solutions **1-NaMo$_5$** in MeCN. S18

Figure S16: Conc. vs abs. plot at the λ_{max} (546 nm) for **1-NaMo$_5$** in MeCN. S18

Figure S17: UV-Vis spectra of 0.5 mM, 0.75 mM, 1 mM, and 2 mM solutions of $(TBA)_4[Ba\{Mo_5O_{13}(OMe)_4NO\}_2]$ in MeCN. S19

Figure S18: Conc. vs abs. plot at the λ_{max} (550 nm) for $(TBA)_4[Ba\{Mo_5O_{13}(OMe)_4NO\}_2]$ in MeCN. S19

Figure S19: UV-Vis spectra of 0.5 mM, 1 mM, 1.5 mM, and 2 mM solutions of $(TBA)_3[Bi\{Mo_5O_{13}(OMe)_4NO\}_2]$ in MeCN. S20

Figure S20: Conc. vs abs. plot at the λ_{max} (560 nm) for $(TBA)_3[Bi\{Mo_5O_{13}(OMe)_4NO\}_2]$ in MeCN. S20

Figure S21: UV-Vis spectra of 0.5 mM, 1 mM, 1.5 mM, and 2 mM solutions of **2-Zr(Mo_5)$_2$** in MeCN. S21

Figure S22: Conc. vs abs. plot at the λ_{max} (588 nm) for **2-Zr(Mo_5)$_2$** in MeCN. S21

Figure S23: UV-Vis spectra of 0.75 mM, 1 mM, 1.5 mM, and 2 mM solutions of **3-Hf(Mo_5)$_2$** in MeCN. S22

Figure S24: Conc. vs abs. plot at the λ_{max} (588 nm) for **3-Hf(Mo_5)$_2$** in MeCN. S22

Figure S25: UV-Vis spectra of 0.75 mM, 1 mM, 1.5 mM, and 2 mM solutions of **4-Th(Mo_5)$_2$** in MeCN. S23

Figure S26: Conc. vs abs. plot at the λ_{max} (572 nm) for **4-Th(Mo_5)$_2$** in MeCN. S23

Figure S27: UV-Vis spectra of 0.5 mM, 1 mM, 1.5 mM, and 2 mM solutions of **5-U(Mo_5)$_2$** in MeCN. S24

Figure S28: Conc. vs abs. plot for the *f-f* transition at 682 nm of **5-U(Mo_5)$_2$** in MeCN. .. S24

Figure S29: Conc. vs abs. plot for the *f-f* transition at 1100 nm of **5-U(Mo_5)$_2$** in MeCN. S25

Figure S30: Conc. vs abs. plot for the *f-f* transition at 1156 nm of **5-U(Mo_5)$_2$** in MeCN. S25

Figure S31: UV-Vis/NIR spectra of **6-U(Mo_5)$_2$** in DCM at room temperature (21 °C) recorded every day for 5 days. The solution was sealed in a screw-top quartz cuvette and was assumed to protected from air and moisture. S26

3. Single crystal X-ray diffraction information **S27**

Table S1: Crystallographic parameters for **2-Zr(Mo_5)$_2$** and **3-Hf(Mo_5)$_2$** S27

Table S2: Crystallographic parameters for **4-Th(Mo_5)$_2$** and **5-U(Mo_5)$_2$** S28

Table S3: Crystallographic parameters for **6-U(Mo_5)$_2$** S29

Table S4: Average bond length data for the structures discussed. All values in Å. A schematic is given below to highlight bond assignments.. S30

Calculation of U^{5+} ionic radius: S31

4. Electrochemistry **S33**

Figure S32: CV of $(TBA)_4[Ba\{Mo_5O_{13}(OMe)_4NO\}_2]$ (1 mM) in MeCN (0.1 M TBA(PF_6)). Scan rate = 200 mv/s. S33

Figure S33: CV of $(TBA)_3[Bi\{Mo_5O_{13}(OMe)_4NO\}_2]$ (1 mM) in MeCN (0.1 M TBA(PF_6)). Scan rate = 200 mv/s. S33

Figure S34: CV of $(TBA)_2[Zr\{Mo_5O_{13}(OMe)_4NO\}_2]$ (1 mM) in MeCN (0.1 M TBA(PF_6)) when scanned to more negative potentials. Scan rate = 200 mv/s...................................... S34

Figure S35: CV of $(TBA)_2[Zr\{Mo_5O_{13}(OMe)_4NO\}_2]$ (1 mM) in MeCN (0.1 M TBA(PF_6)) when scanned to more negative potentials. Scan rate = 200 mv/s...................................... S34

Figure S36: CV of $(TBA)_2[U\{Mo_5O_{13}(OMe)_4NO\}_2]$ (1 mM) in MeCN, DCM, and THF (0.1 M TBA(PF_6)). Scan rate = 200 mv/s. S35

Figure S37: Pre bulk electrolysis CV of $(TBA)_2[U\{Mo_5O_{13}(OMe)_4NO\}_2]$ (1 mM) in MeCN (0.1 M TBA(PF_6)). Scan rate = 200 mv/s. S35

Figure S38: Bulk oxidation of a 1 mM solution of $(TBA)_2[U\{Mo_5O_{13}(OMe)_4NO\}_2]$ in DCM (0.1 M TBA(PF_6). Chronoamperometry was performed at +0.78 V vs Fc/Fc^+. S36

Figure S39: Post bulk electrolysis CV of $(TBA)_2[U\{Mo_5O_{13}(OMe)_4NO\}_2]$ (1 mM) in MeCN (0.1 M TBA(PF_6)). Scan rate = 200 mv/s. S36

Figure S40: CV of crude $(TBA)[U\{Mo_5O_{13}(OMe)_4NO\}_2]$ (1 mM) in DCM (0.1 M TBA(PF_6)) obtained by oxidation of $(TBA)_2[U\{Mo_5O_{13}(OMe)_4NO\}_2]$ with an excess of [NO][PF_6]. Scan rate = 200 mv/s. S37

1. ^{1}H NMR spectra

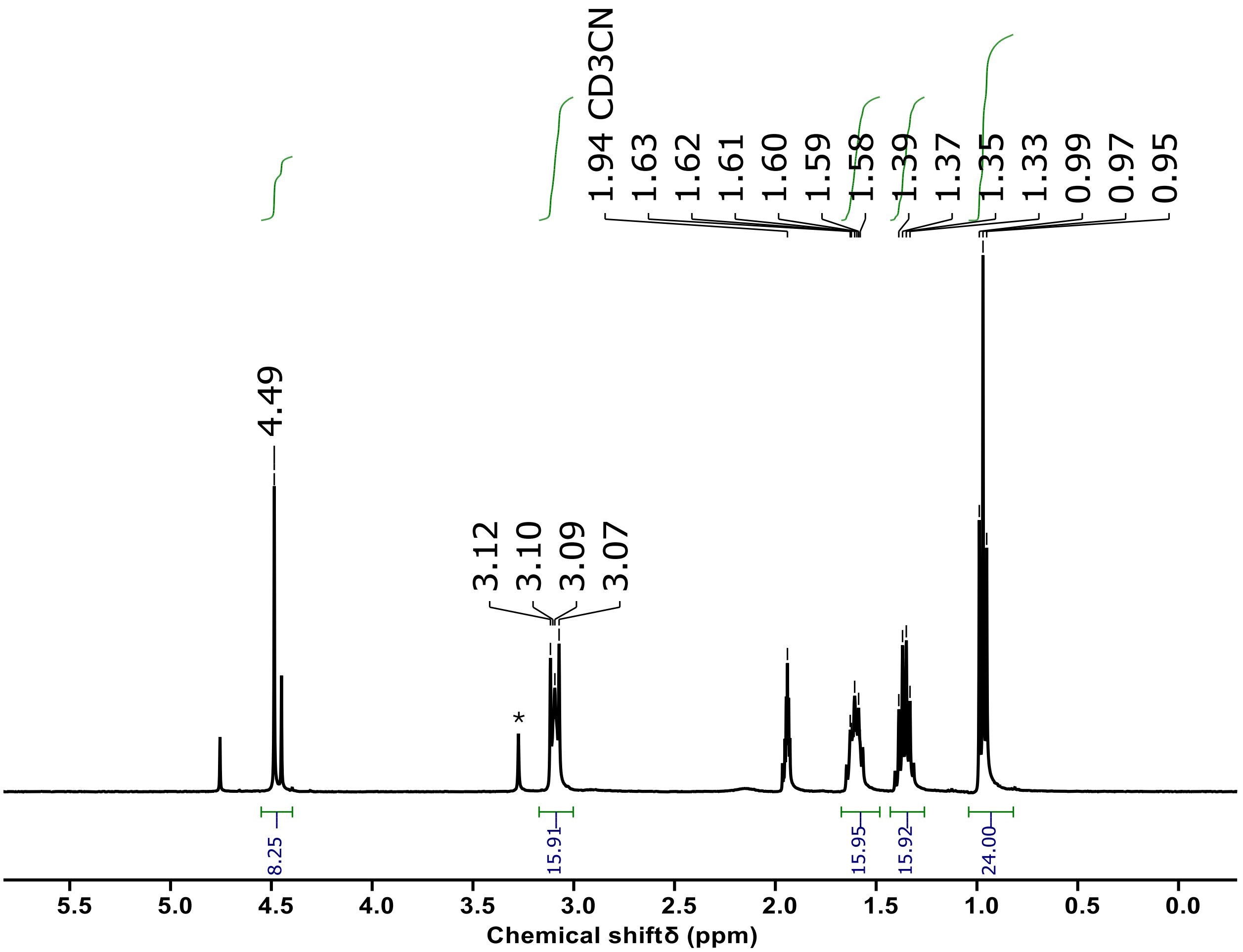

Figure S1: ^{1}H NMR spectrum (400 MHz) of $(TBA)_2[Mo_5O_{13}(OMe)_4NO][Na(MeOH)]$ (**1-NaMo$_5$**) in CD_3CN. The peak marked with an asterisk corresponds to MeOH.

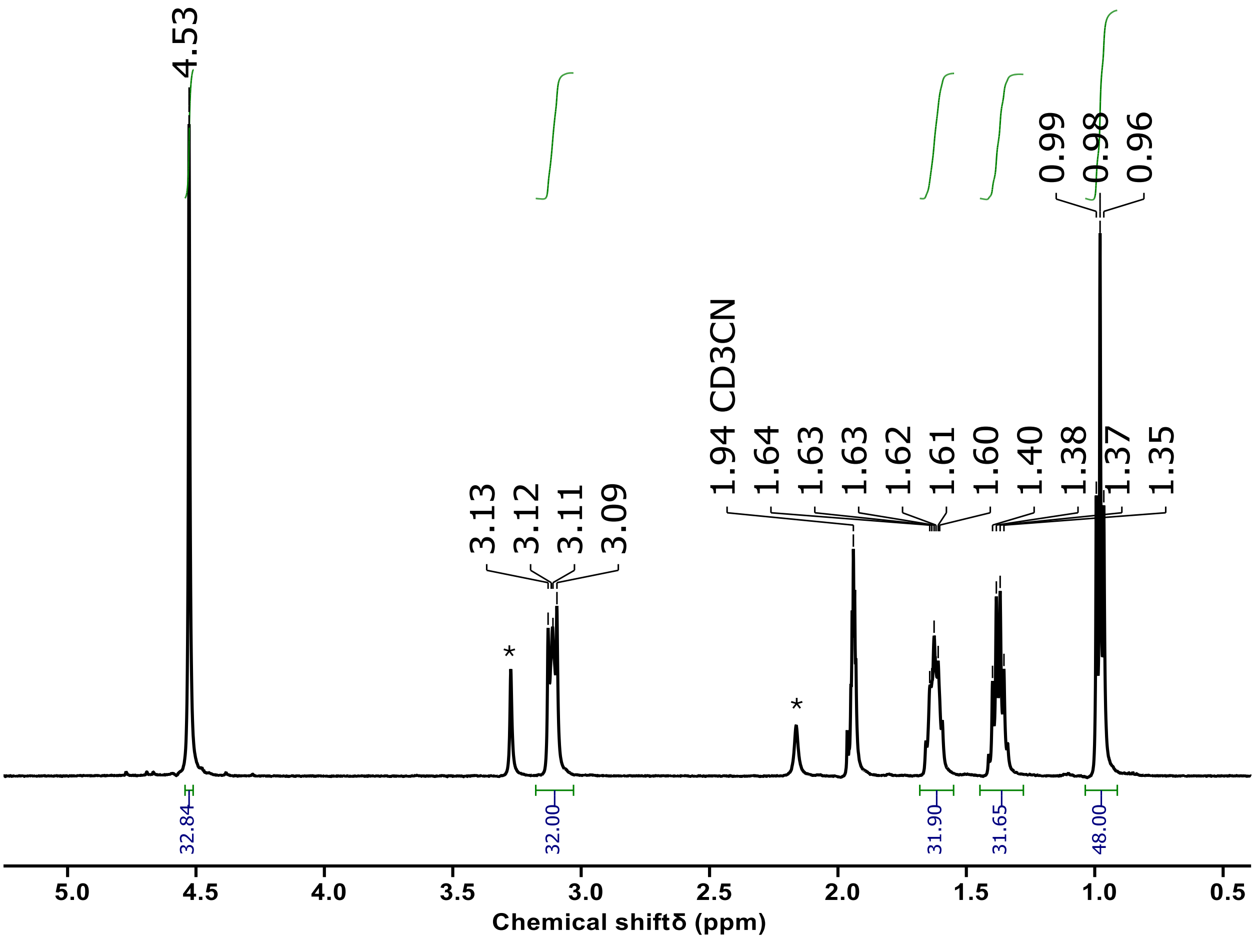

Figure S2: ^{1}H NMR spectrum (500 MHz) of $(TBA)_4[Ba\{Mo_5O_{13}(OMe)_4NO\}_2]$ in CD_3CN. Peaks marked with an asterisk correspond to MeOH.

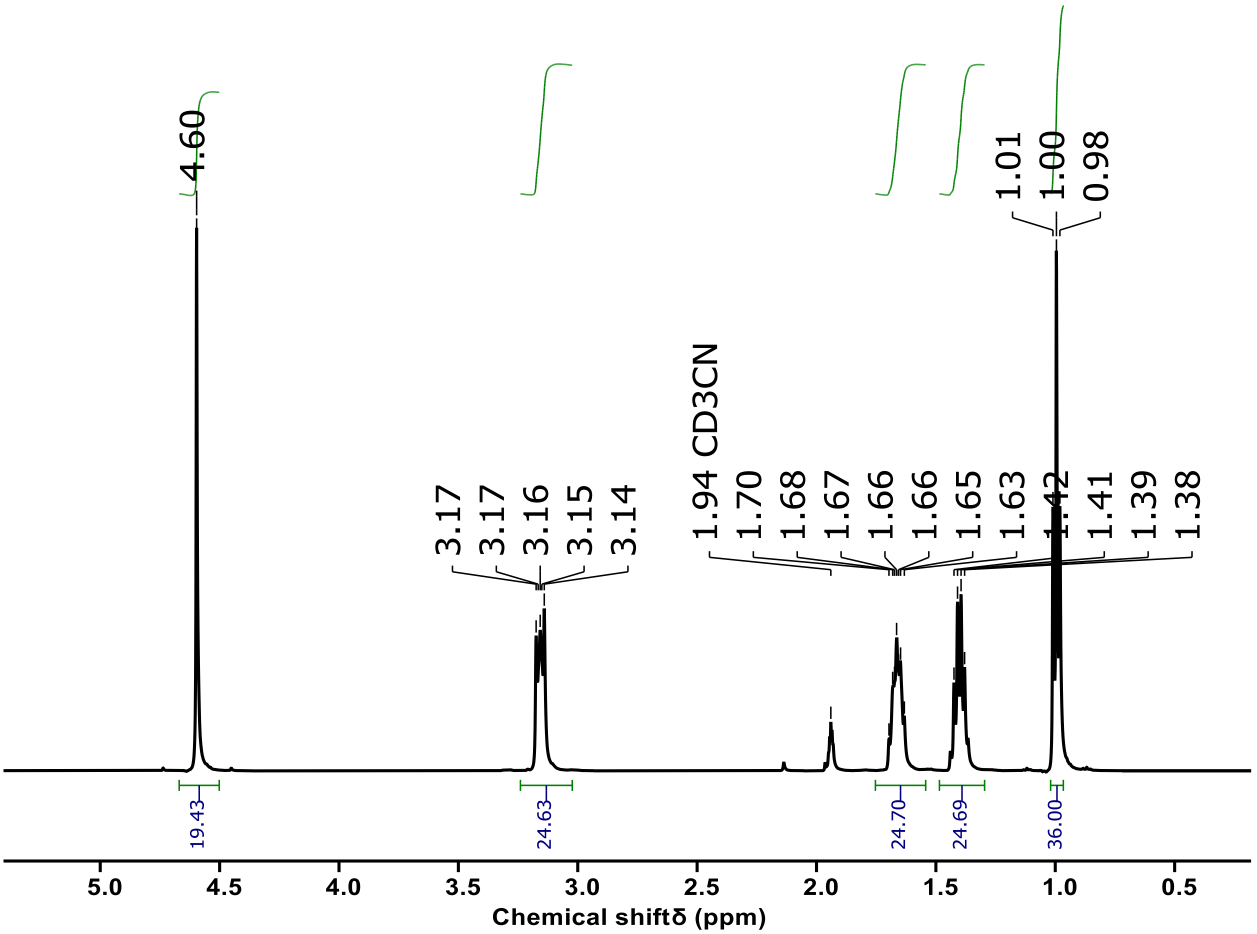

Figure S3: ^1H NMR spectrum (500 MHz) of $(TBA)_3[Bi\{Mo_5O_{13}(OMe)_4NO\}_2]$ in CD_3CN.

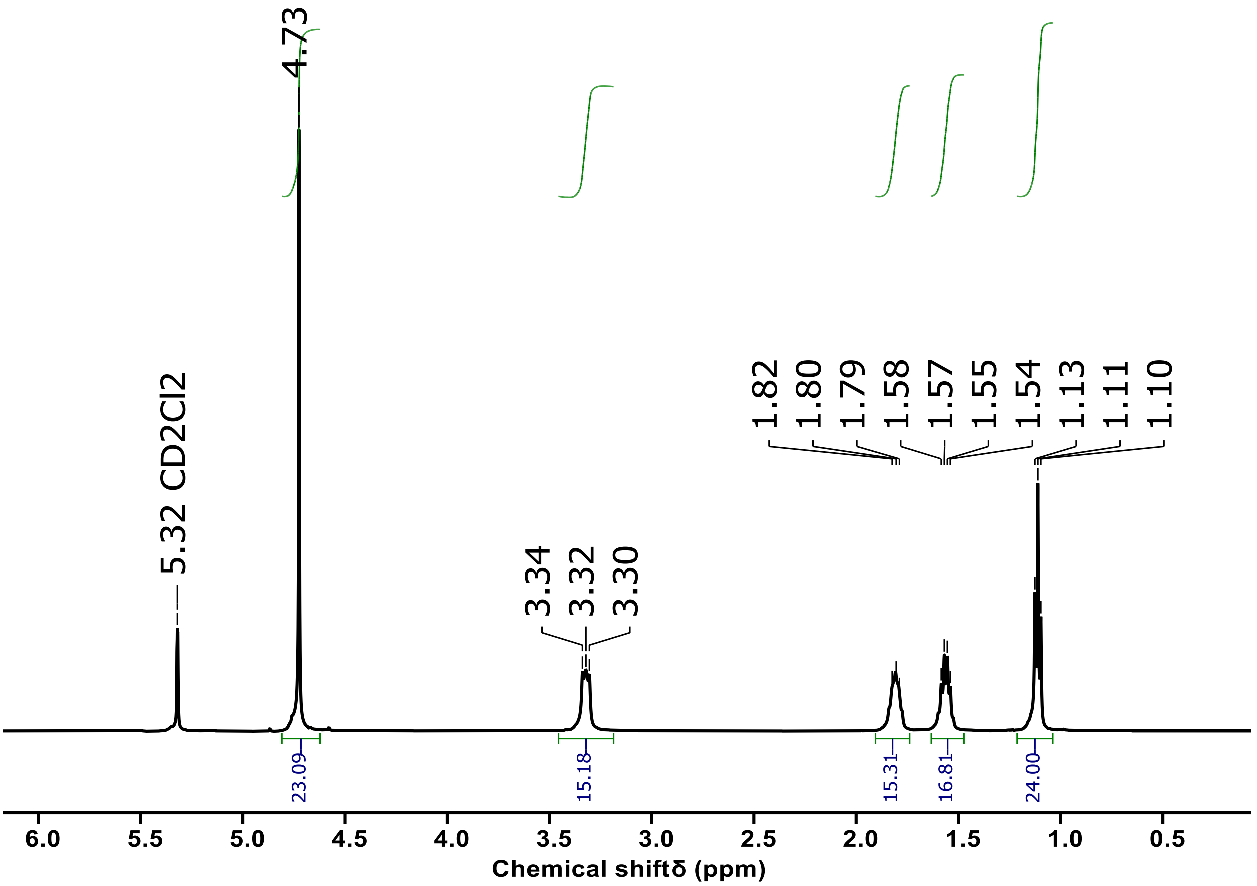

Figure S4: ^{1}H NMR spectrum (500 MHz) of $(TBA)_2[Zr\{Mo_5O_{13}(OMe)_4NO\}_2]$ (**2-Zr(Mo$_5$)$_2$**) in CD_2Cl_2.

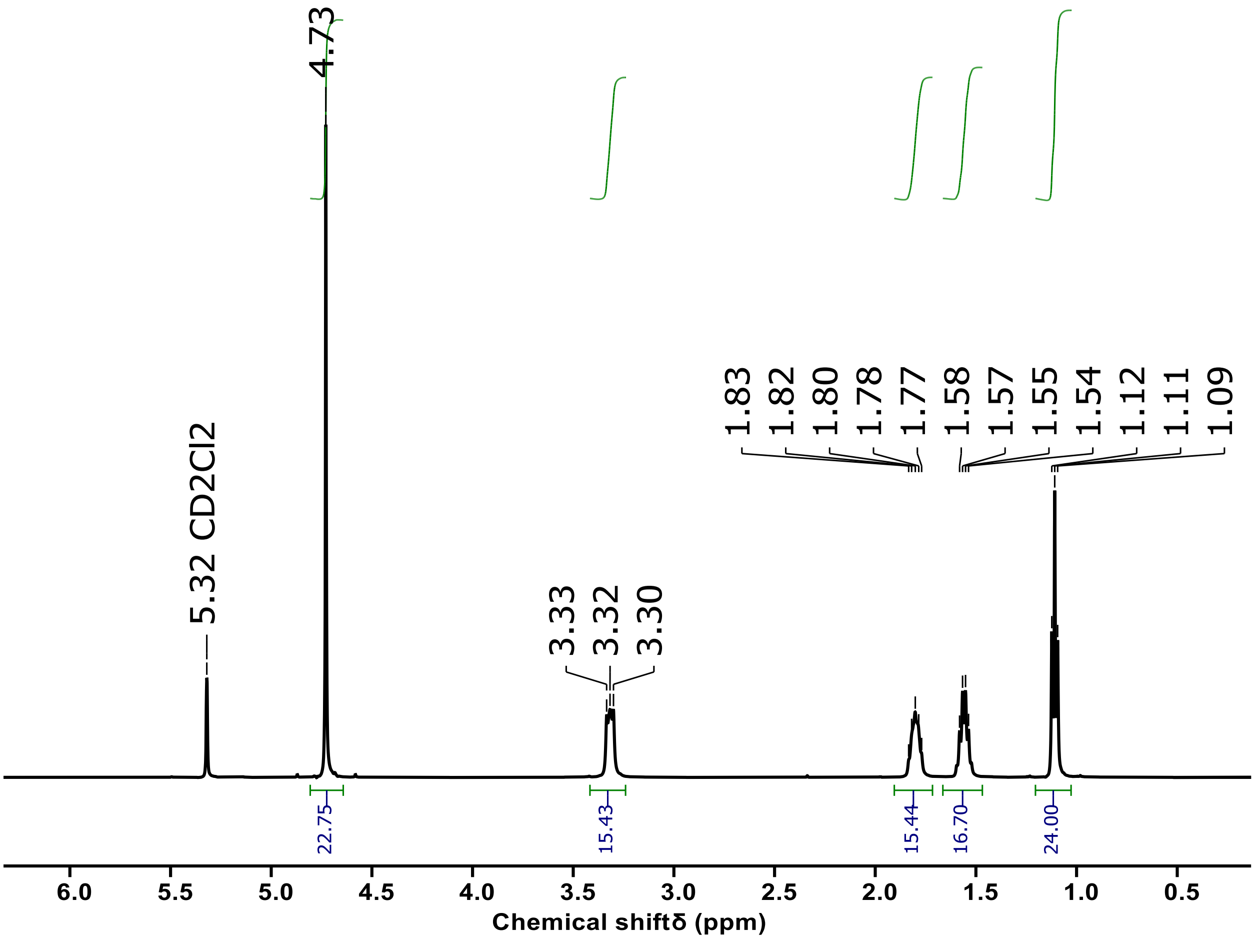

Figure S5: ^{1}H NMR spectrum (500 MHz) of $(TBA)_2[Hf\{Mo_5O_{13}(OMe)_4NO\}_2]$ (**3-Hf(Mo$_5$)$_2$**) in CD_2Cl_2.

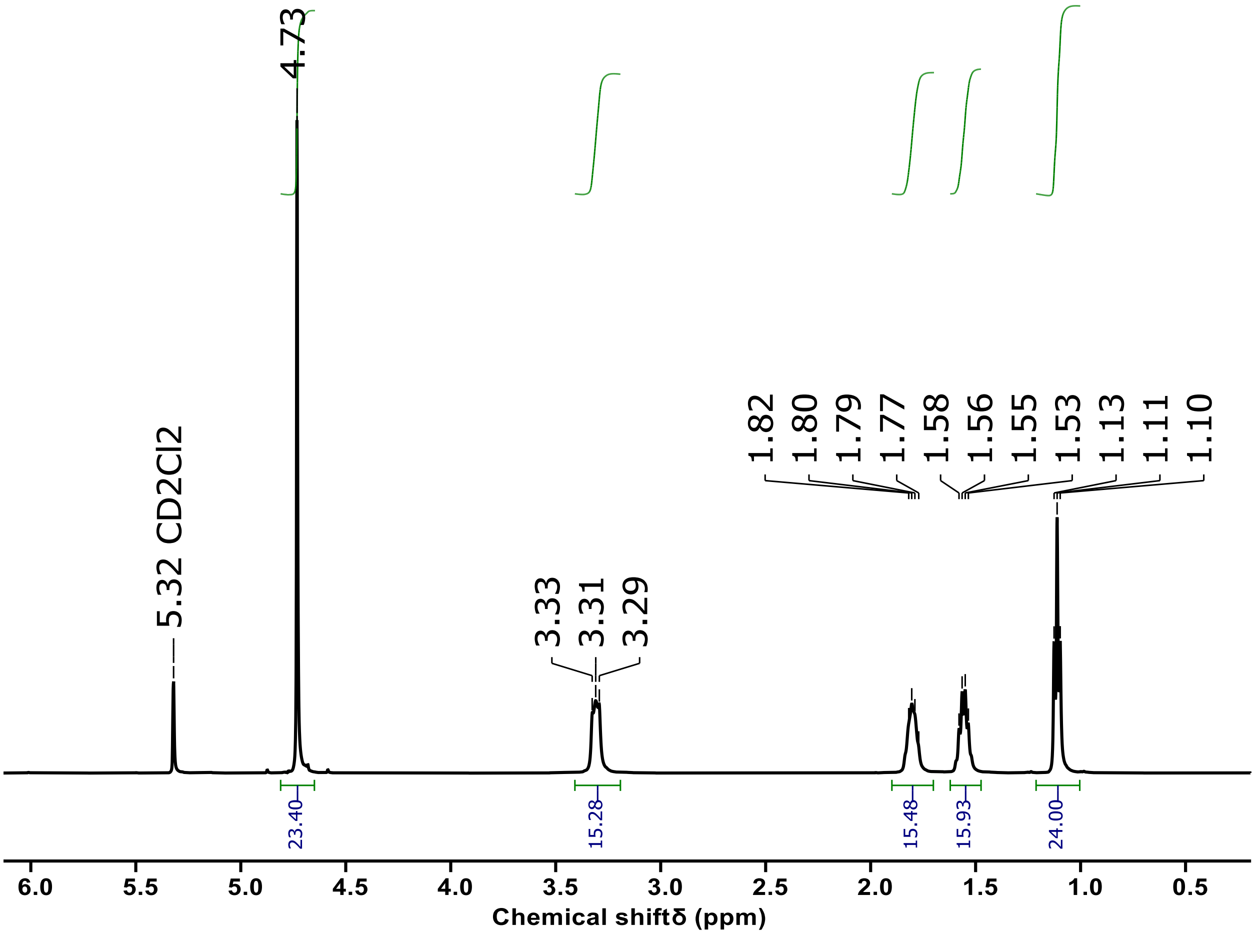

Figure S6: ^{1}H NMR spectrum (500 MHz) of $(TBA)_2[Th\{Mo_5O_{13}(OMe)_4NO\}_2]$ (**4-Th(Mo$_5$)$_2$**) in CD_2Cl_2.

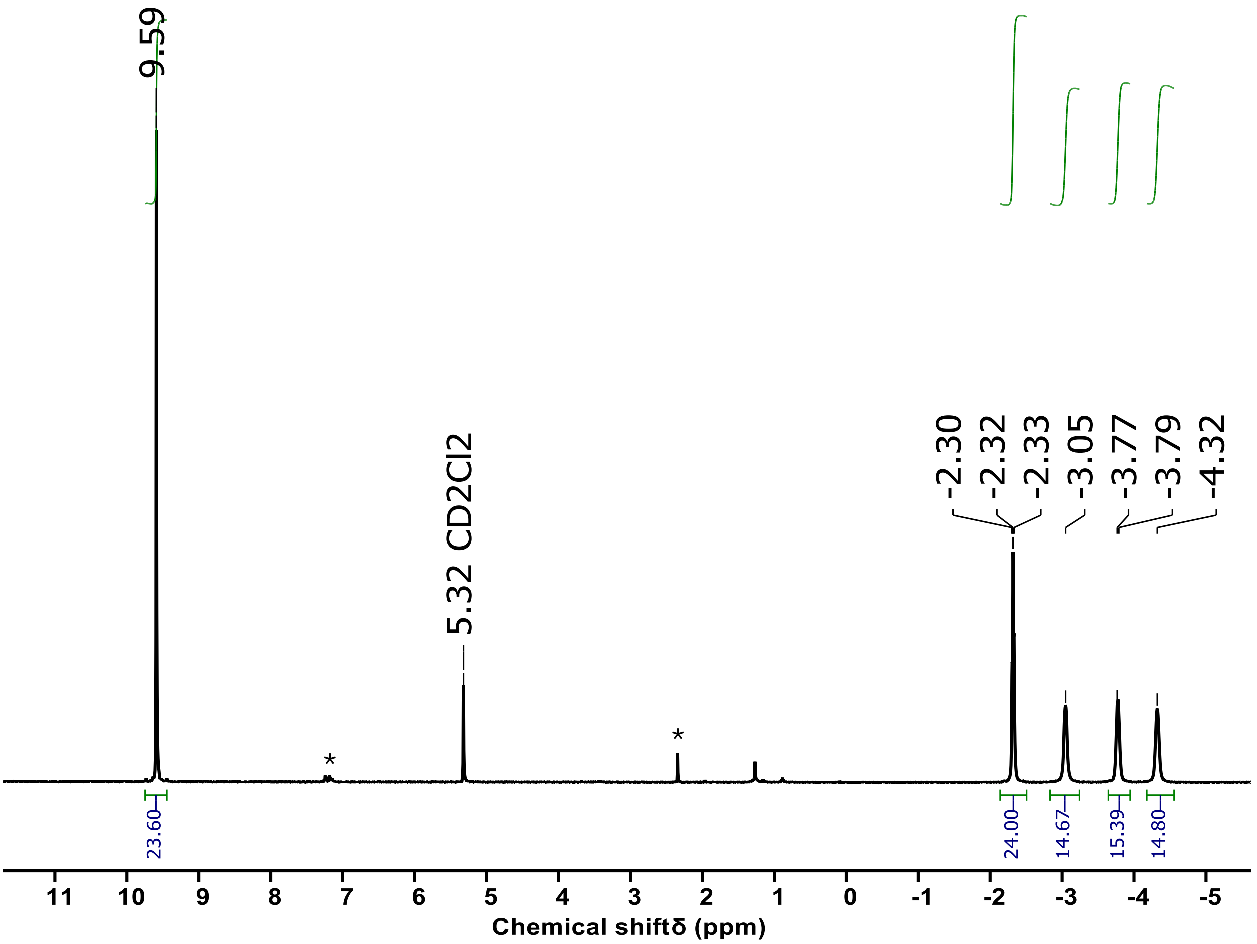

Figure S7: ^{1}H NMR spectrum (500 MHz) of $(TBA)_2[U\{Mo_5O_{13}(OMe)_4NO\}_2]$ (**5-U(Mo$_5$)$_2$**) in CD_2Cl_2. Peaks marked with asterisks correspond to toluene impurity.

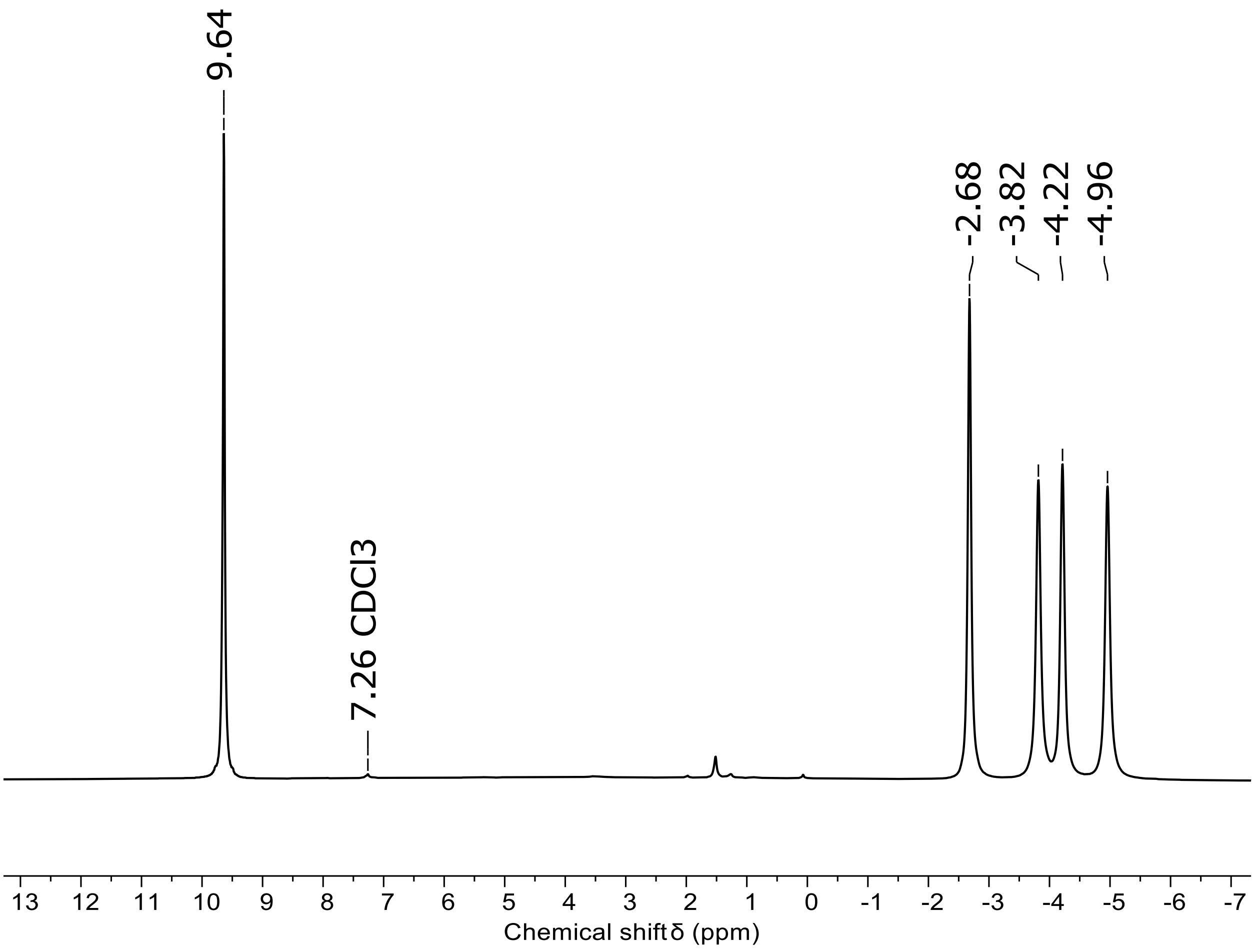

Figure S8: ^{1}H NMR spectrum (500 MHz) of $(TBA)_2[U\{Mo_5O_{13}(OMe)_4NO\}_2]$ (**5-U(Mo$_5$)$_2$**) in $CDCl_3$.

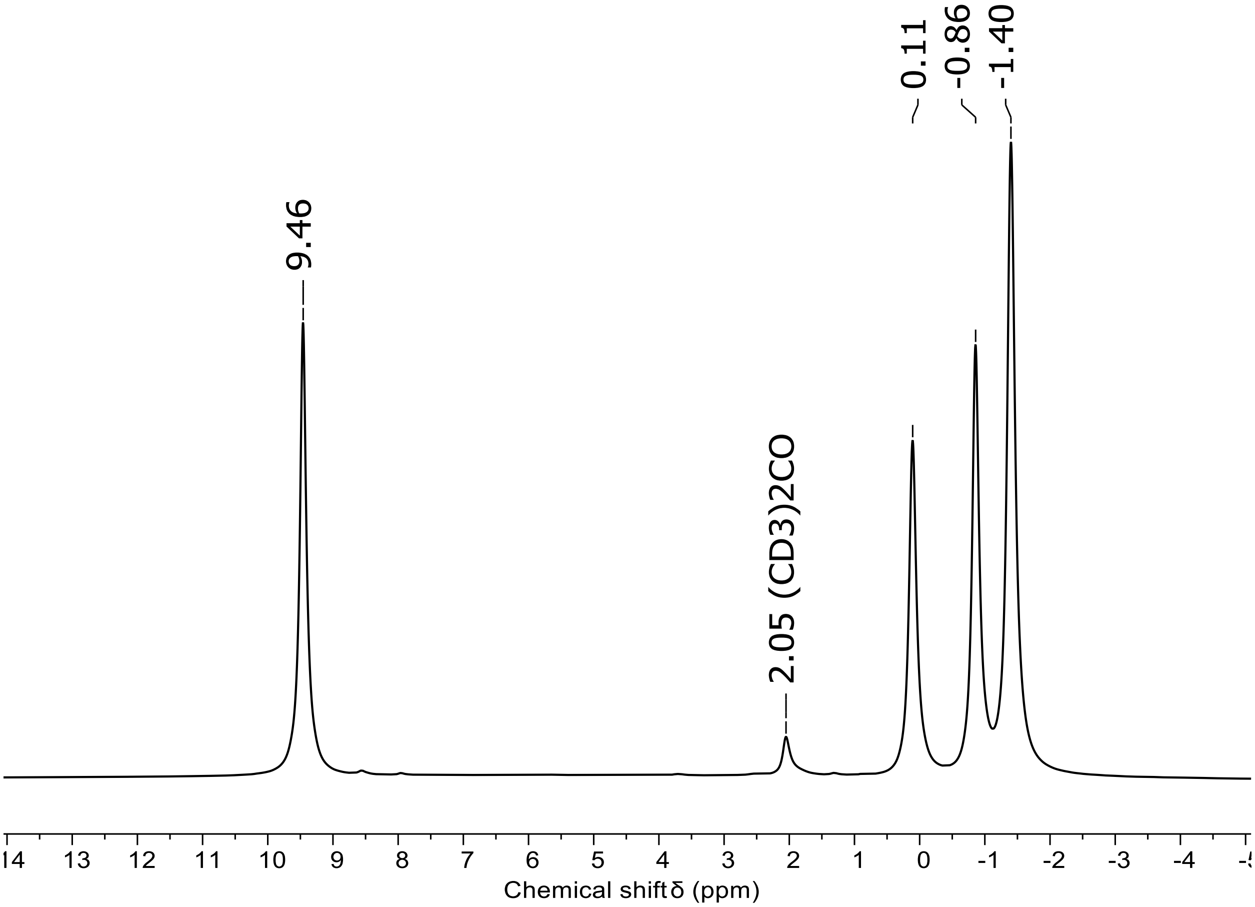

Figure S9: ^{1}H NMR spectrum (500 MHz) of $(TBA)_2[U\{Mo_5O_{13}(OMe)_4NO\}_2]$ (**5-U(Mo$_5$)$_2$**) in acetone-d_6.

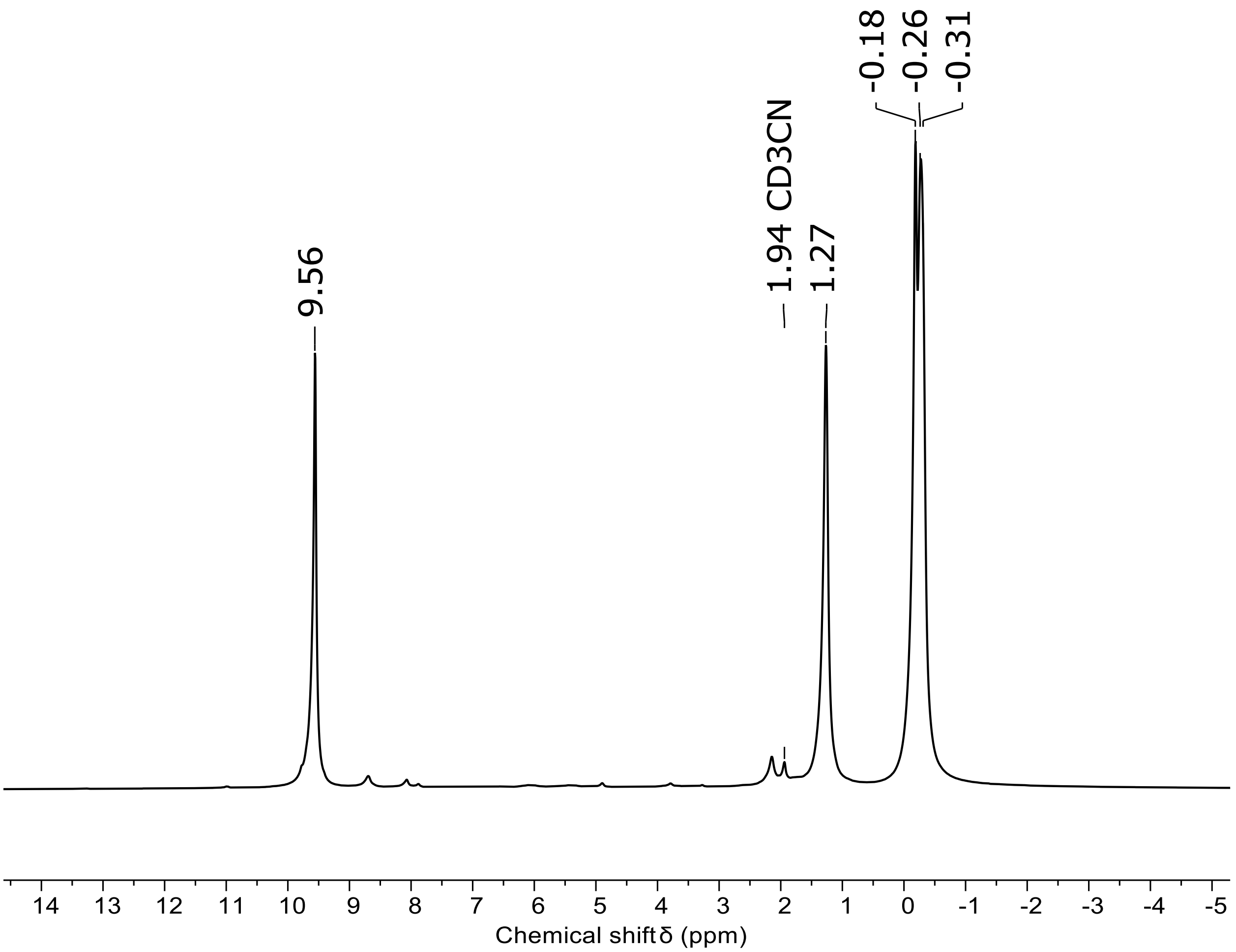

Figure S10: ^{1}H NMR spectrum (500 MHz) of $(TBA)_2[U\{Mo_5O_{13}(OMe)_4NO\}_2]$ (**5-U(Mo$_5$)$_2$**) in CD_3CN.

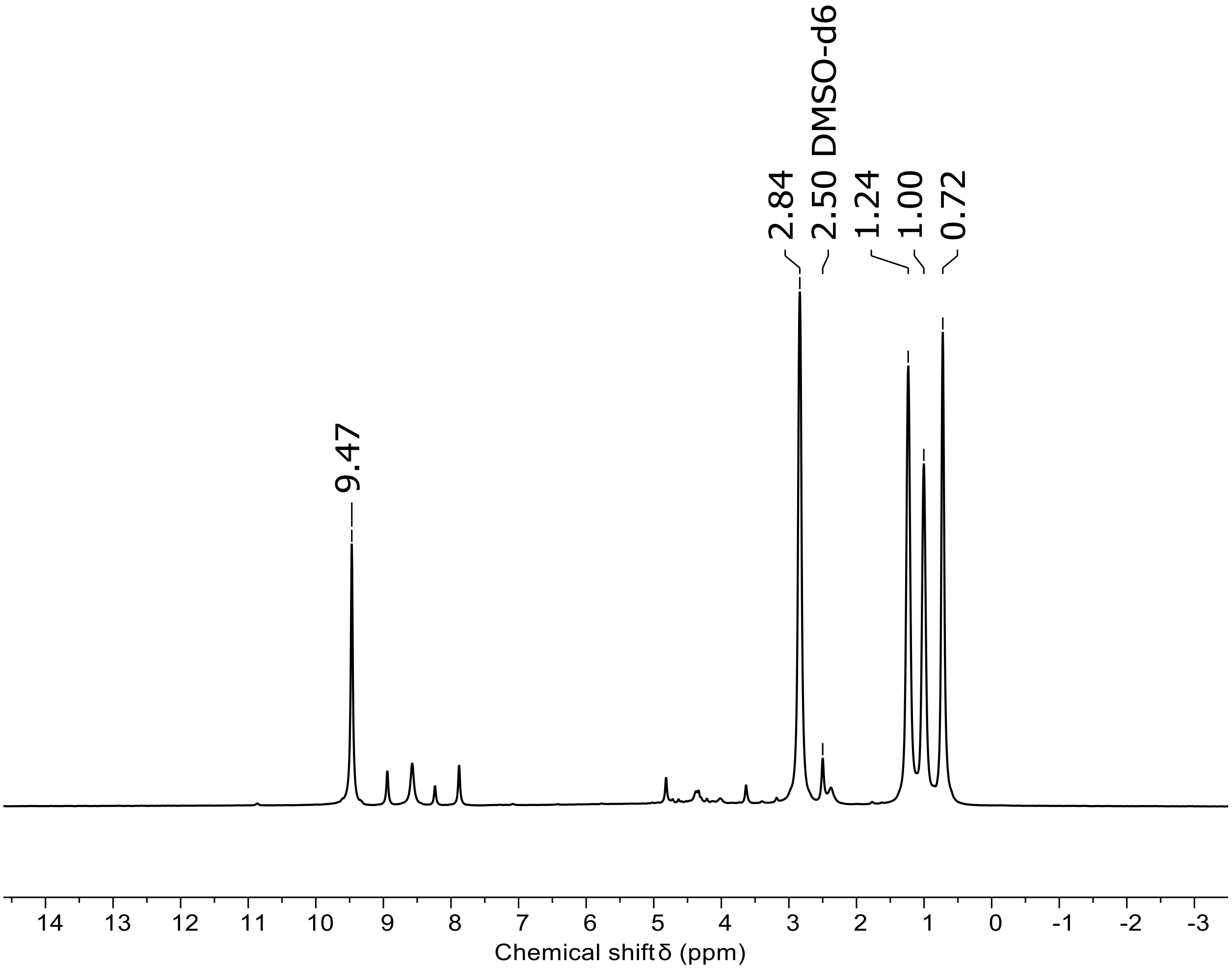

Figure S11: ^{1}H NMR spectrum (500 MHz) of $(TBA)_2[U\{Mo_5O_{13}(OMe)_4NO\}_2]$ (**5-U(Mo₅)₂**) in DMSO-d_6.

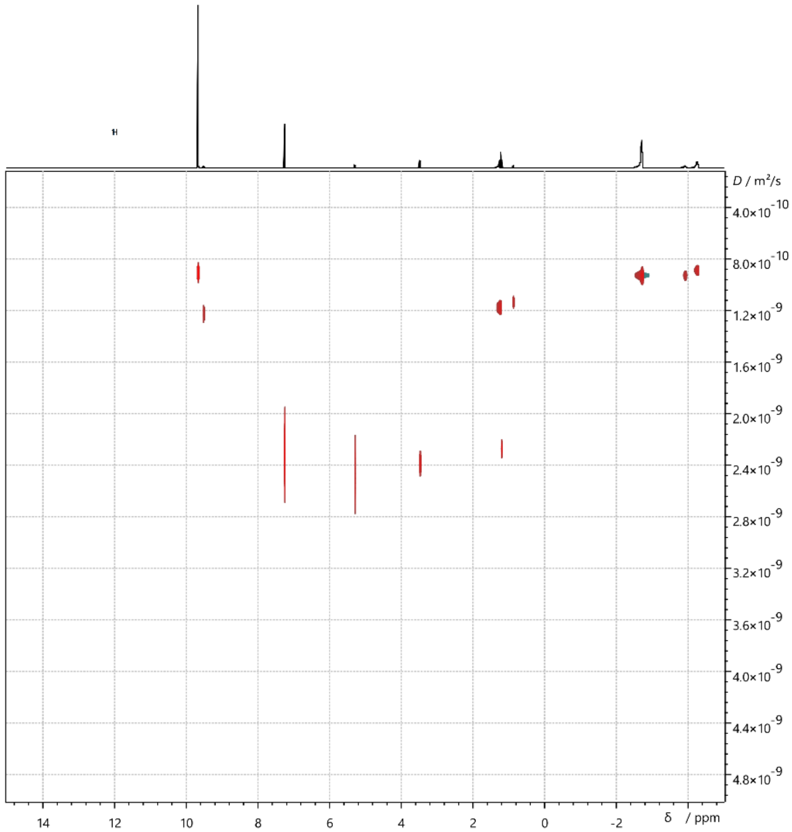

Pos (ppm)	σ-Pos	D (m²/s)	σ-D
9.663	0.000	9.06e-10	4.16e-11
9.516	0.000	1.22e-09	1.06e-10
7.249	0.000	2.32e-09	3.03e-10
5.285	0.000	2.47e-09	4.18e-10
3.469	0.000	2.39e-09	9.65e-11
1.342	0.000	1.18e-09	4.23e-11
1.191	0.000	2.27e-09	5.17e-11
0.887	0.000	1.13e-09	5.96e-11
-2.723	0.000	9.29e-10	4.42e-11
-3.915	0.000	9.29e-10	5.63e-11
-4.188	0.000	8.89e-10	4.10e-11

Figure S12: ^{1}H DOSY NMR spectrum (500 MHz) of a 2mM solution $(TBA)_2[U\{Mo_5O_{13}(OMe)_4NO\}_2]$ (**5-U(Mo$_5$)$_2$**) in $CDCl_3$. The obtained diffusion coefficient (D in m^2/s) for each peak in the ^{1}H NMR spectrum is given with the standard deviation (σ-D). The peaks at −2.723, −3.915, and −4.188 ppm can be assigned to the TBA cation, while the peak at 9.663 ppm is assigned to $\{U(Mo_5)_2\}$ cluster.

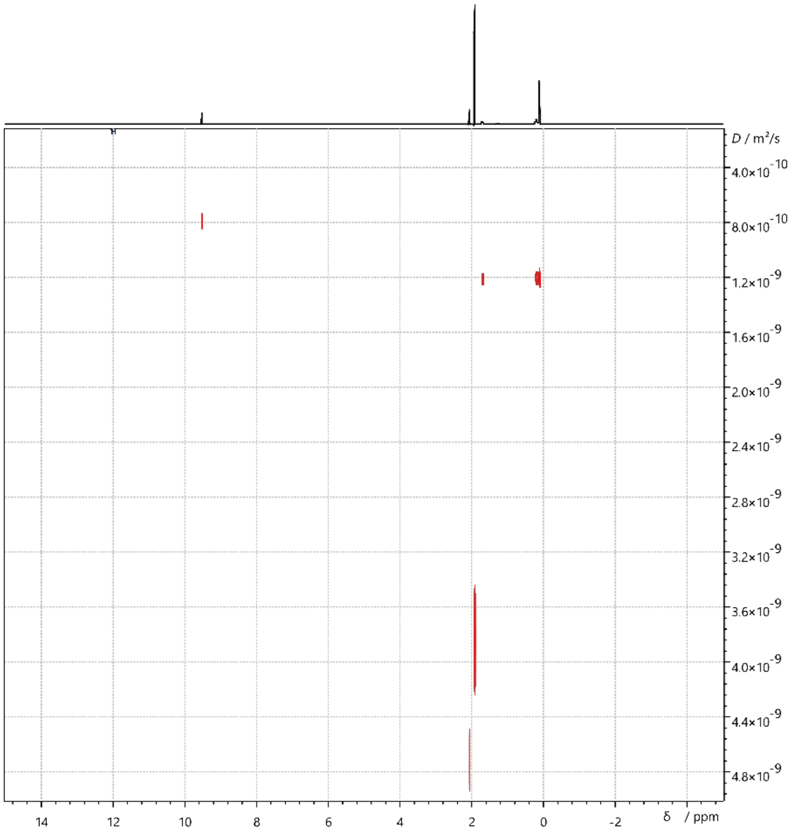

Pos (ppm)	σ-Pos	D (m²/s)	σ-D
9.519	0.000	7.91e-10	2.17e-11
2.037	0.000	4.71e-09	4.43e-10
1.913	0.000	3.84e-09	3.85e-10
1.696	0.000	1.21e-09	2.78e-11
1.251	0.000	1.39e-09	7.27e-11
0.195	0.000	1.20e-09	5.16e-11
0.093	0.000	1.22e-09	3.24e-11

Figure S13: ^1H DOSY NMR spectrum (500 MHz) of a 2 mM solution of $(TBA)_2[U\{Mo_5O_{13}(OMe)_4NO\}_2]$ (**5-U(Mo$_5$)$_2$**) in CD_3CN. The obtained diffusion coefficient (D in m^2/s) for each peak in the ^1H NMR spectrum is given with the standard deviation (σ-D). The peaks at 0.093, 0.195, 1.251 and 1.696 ppm can be assigned to the TBA cation, while the peak at 9.519 ppm is assigned to $\{U(Mo_5)_2\}$ cluster.

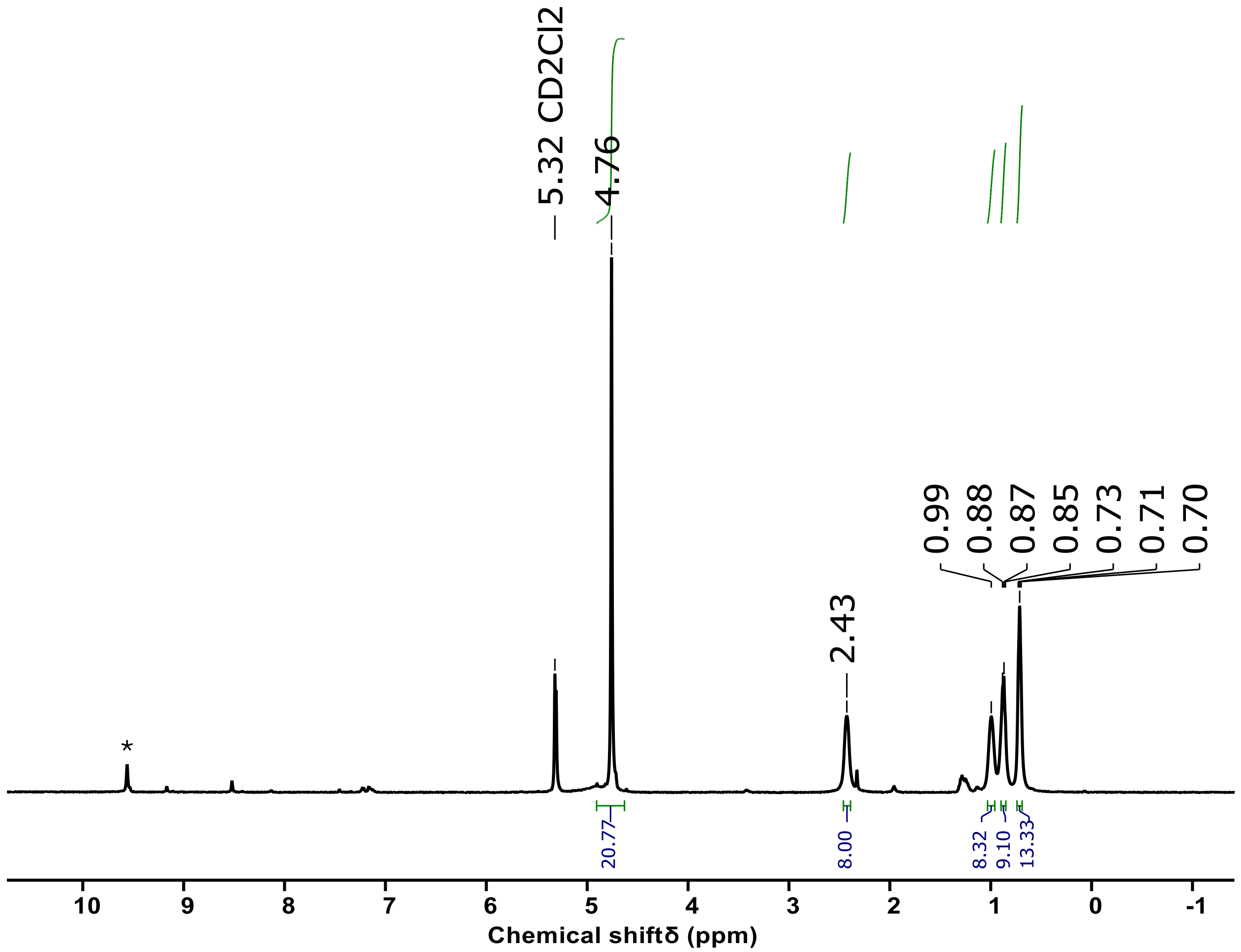

Figure S14: ^{1}H NMR spectrum (500 MHz) of crude (TBA)[U{$Mo_5O_{13}(OMe)_4NO$}$_2$] (**6-U(Mo$_5$)$_2$**), obtained from oxidation of $(TBA)_2$[U{$Mo_5O_{13}(OMe)_4NO$}$_2$] (**5-U(Mo$_5$)$_2$**) with an excess of [NO][PF_6]. Spectrum recorded in CD_2Cl_2. The peak marked with an asterisk corresponds to the -OMe groups of **5-U(Mo$_5$)$_2$**.

2. Electronic absorption spectra

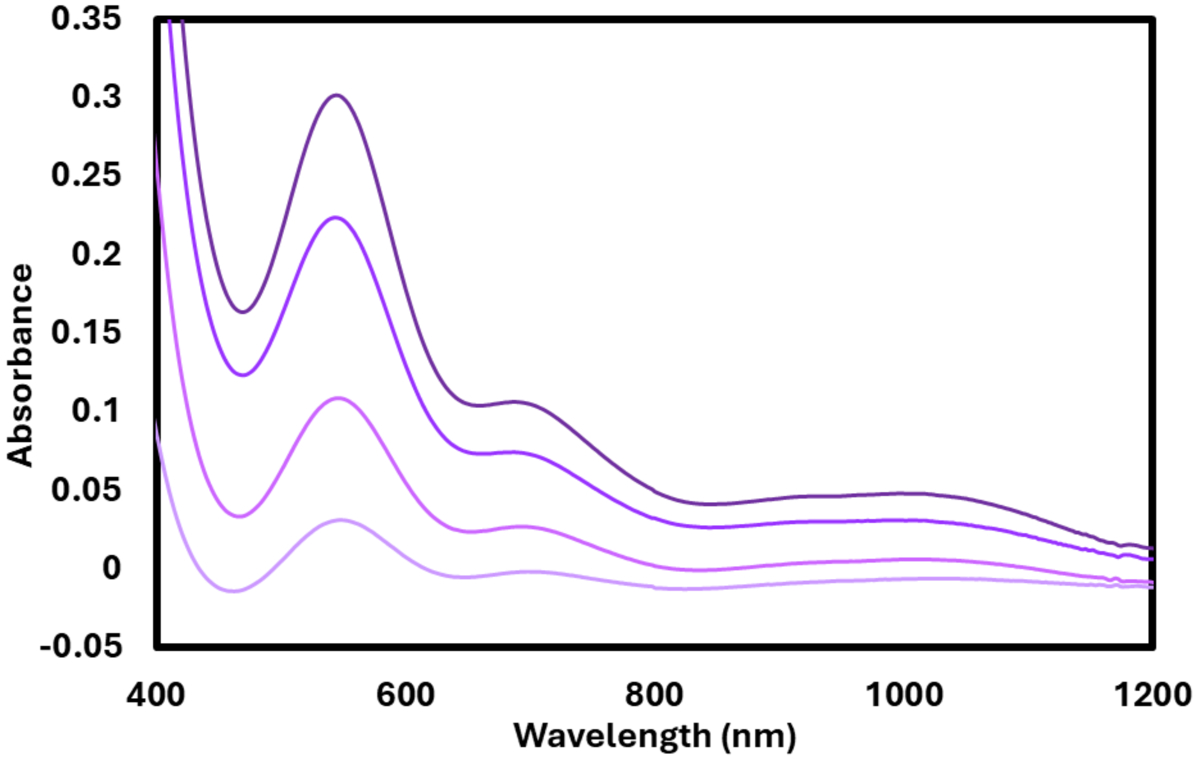

Figure S15: UV-Vis spectra of 1 mM, 2 mM, 3 mM, and 4 mM solutions **1-NaMo$_5$** in MeCN.

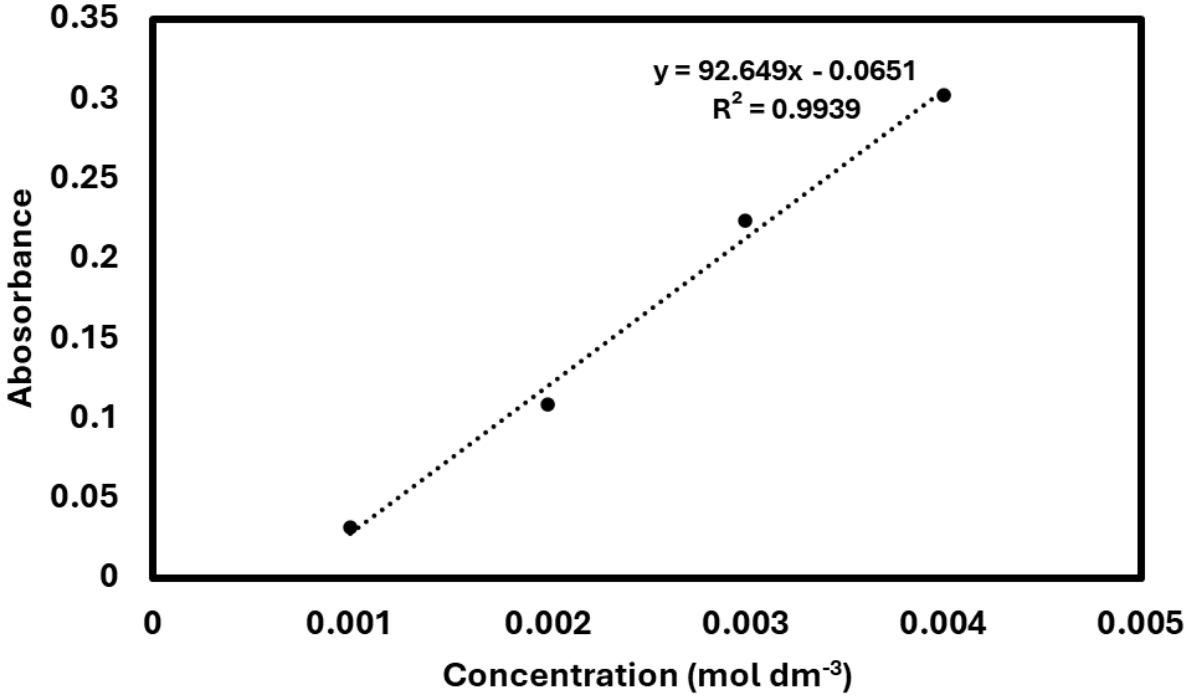

Figure S16: Conc. vs abs. plot at the λ_{max} (546 nm) for **1-NaMo$_5$** in MeCN.

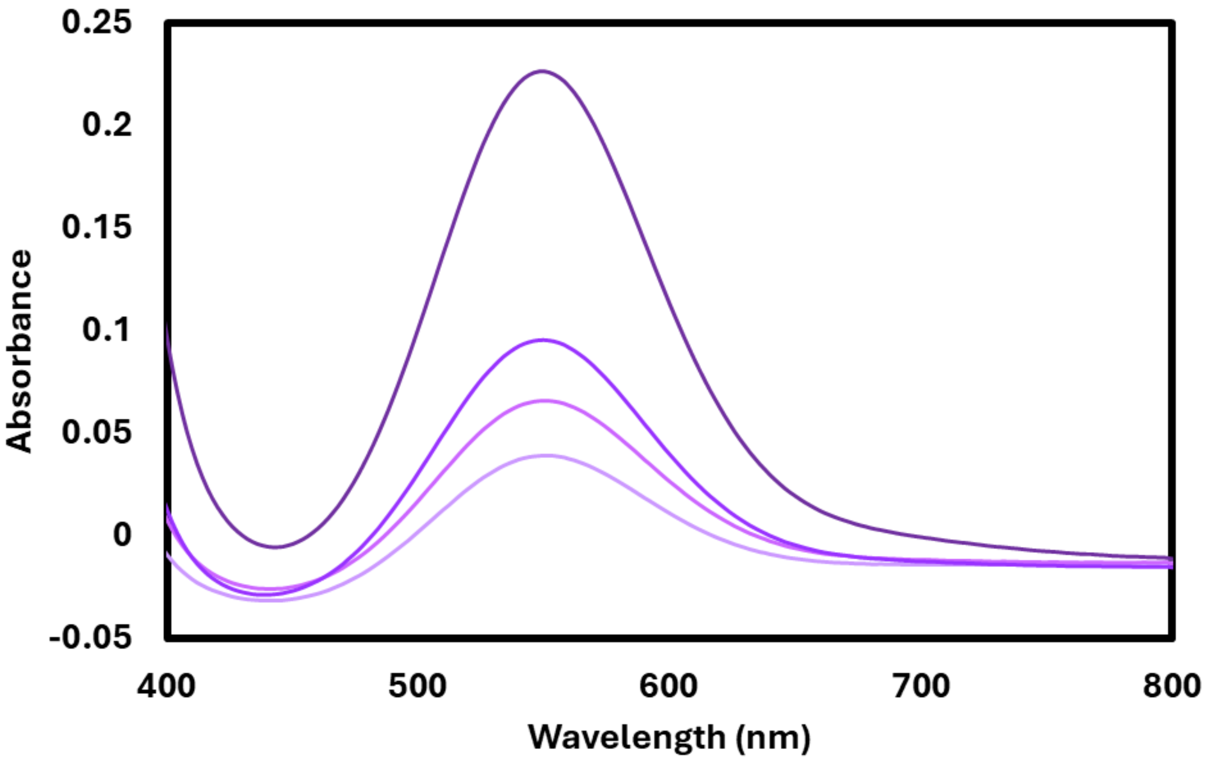

Figure S17: UV-Vis spectra of 0.5 mM, 0.75 mM, 1 mM, and 2 mM solutions of $(TBA)_4[Ba\{Mo_5O_{13}(OMe)_4NO\}_2]$ in MeCN.

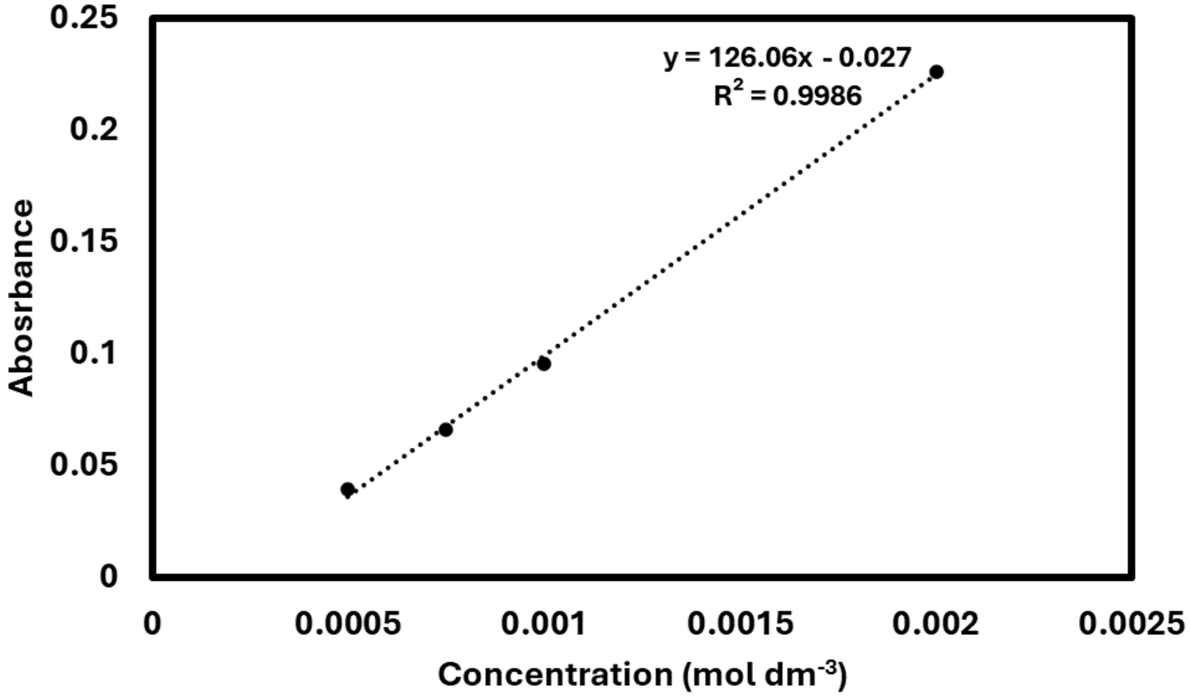

Figure S18: Conc. vs abs. plot at the λ_{max} (550 nm) for $(TBA)_4[Ba\{Mo_5O_{13}(OMe)_4NO\}_2]$ in MeCN.

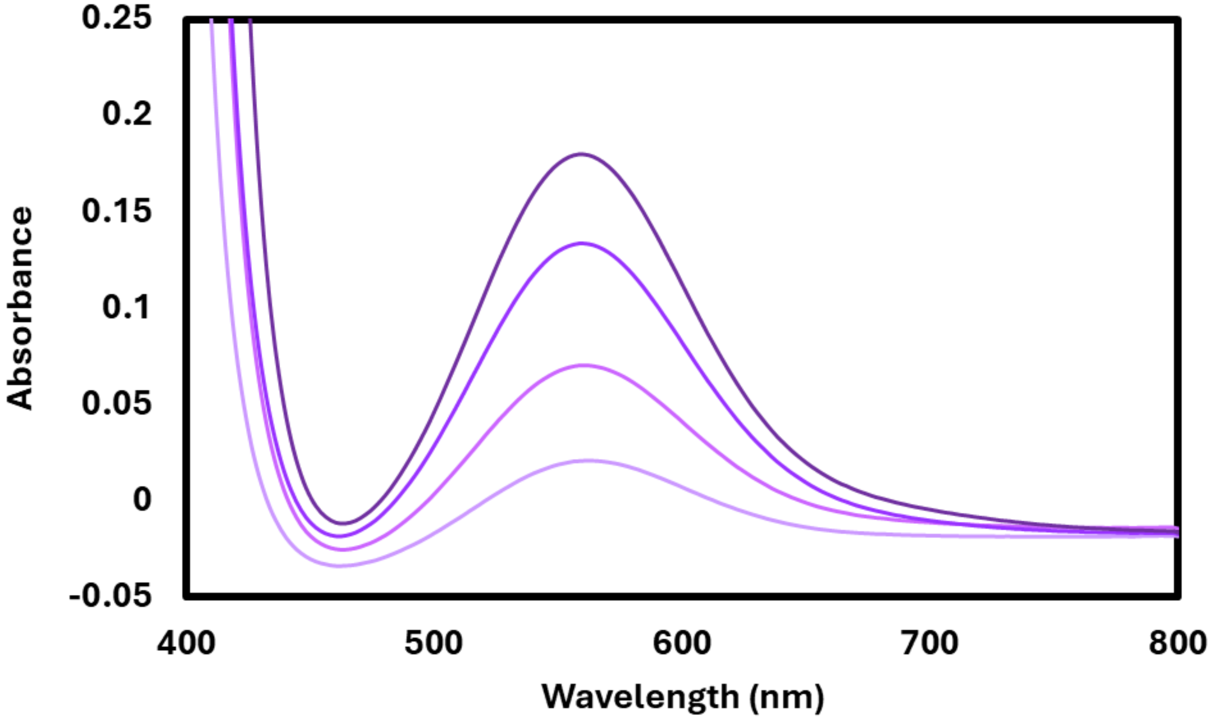

Figure S19: UV-Vis spectra of 0.5 mM, 1 mM, 1.5 mM, and 2 mM solutions of $(TBA)_3[Bi\{Mo_5O_{13}(OMe)_4NO\}_2]$ in MeCN.

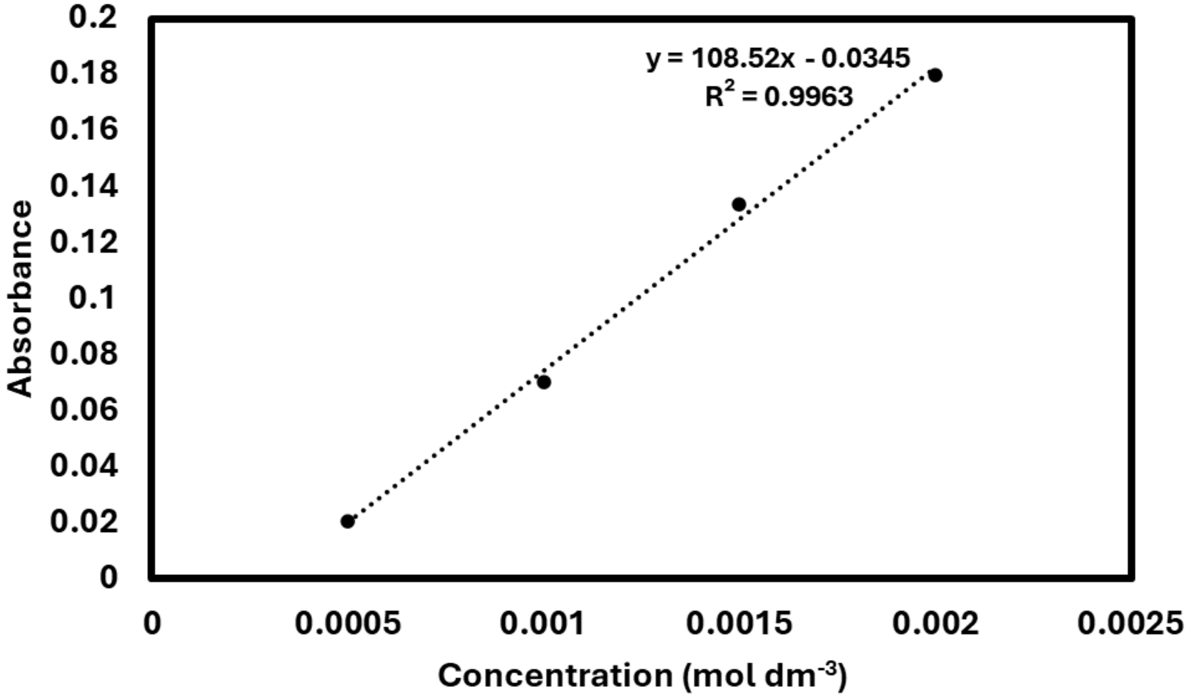

Figure S20: Conc. vs abs. plot at the λ_{max} (560 nm) for $(TBA)_3[Bi\{Mo_5O_{13}(OMe)_4NO\}_2]$ in MeCN.

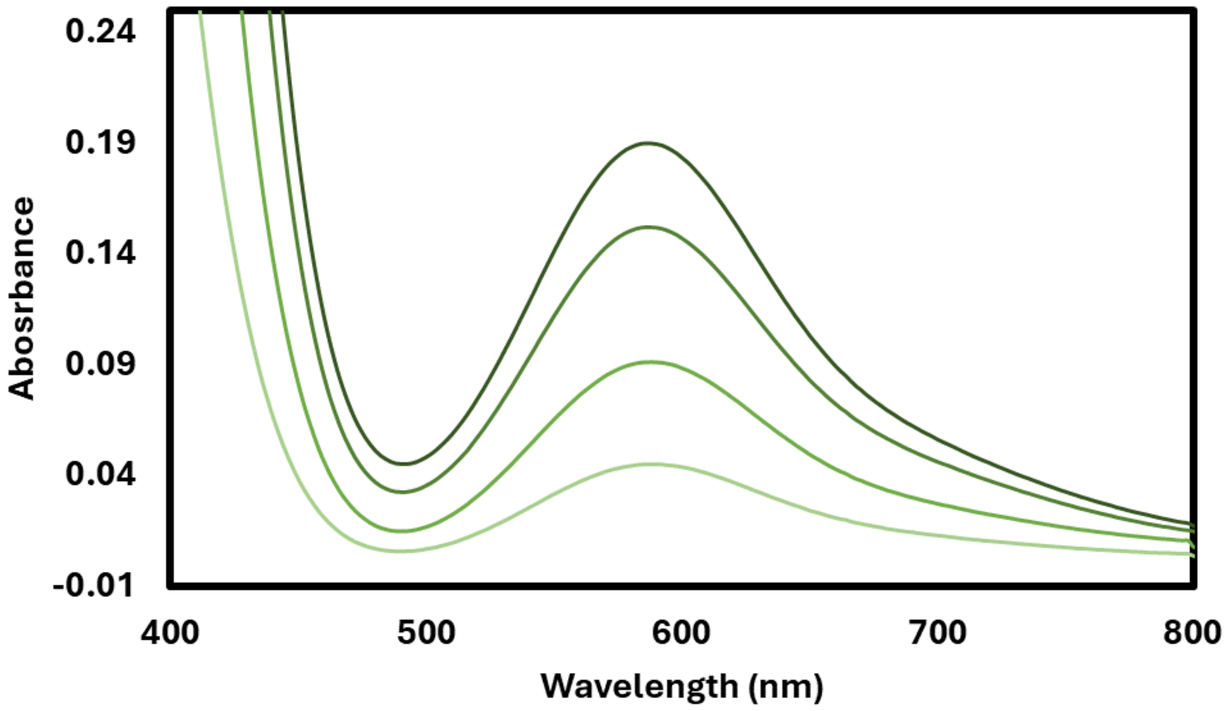

Figure S21: UV-Vis spectra of 0.5 mM, 1 mM, 1.5 mM, and 2 mM solutions of **2-Zr(Mo$_5$)$_2$** in MeCN.

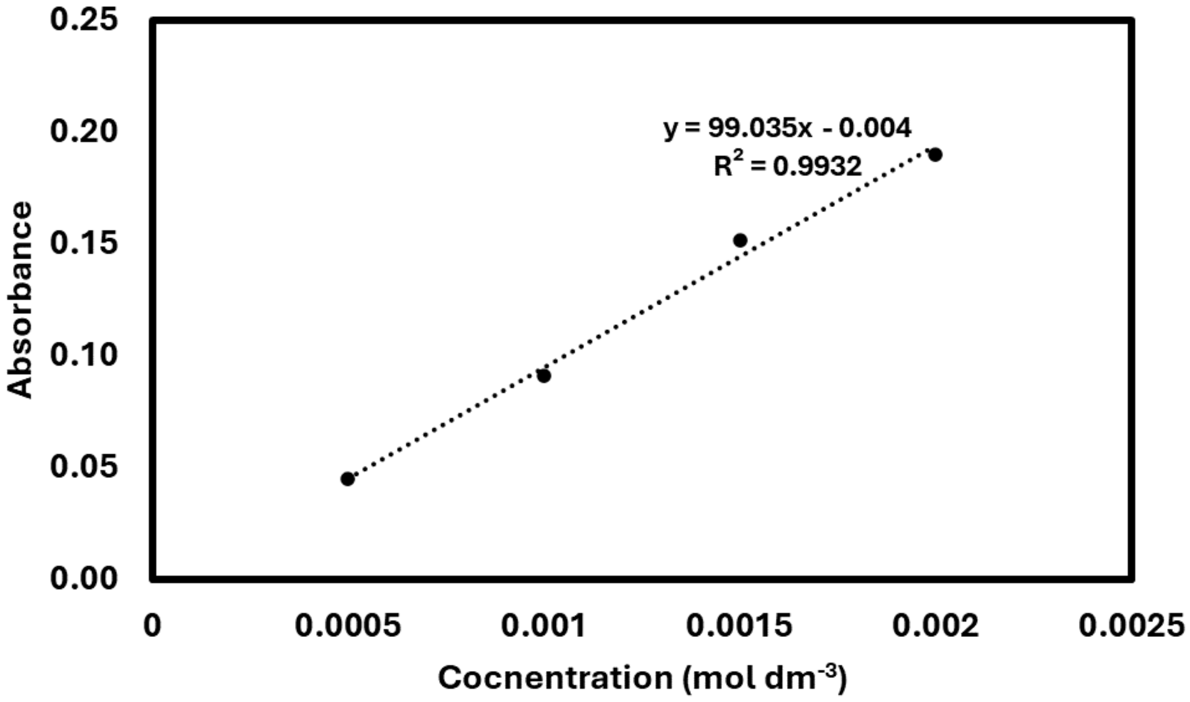

Figure S22: Conc. vs abs. plot at the λ_{max} (588 nm) for **2-Zr(Mo$_5$)$_2$** in MeCN.

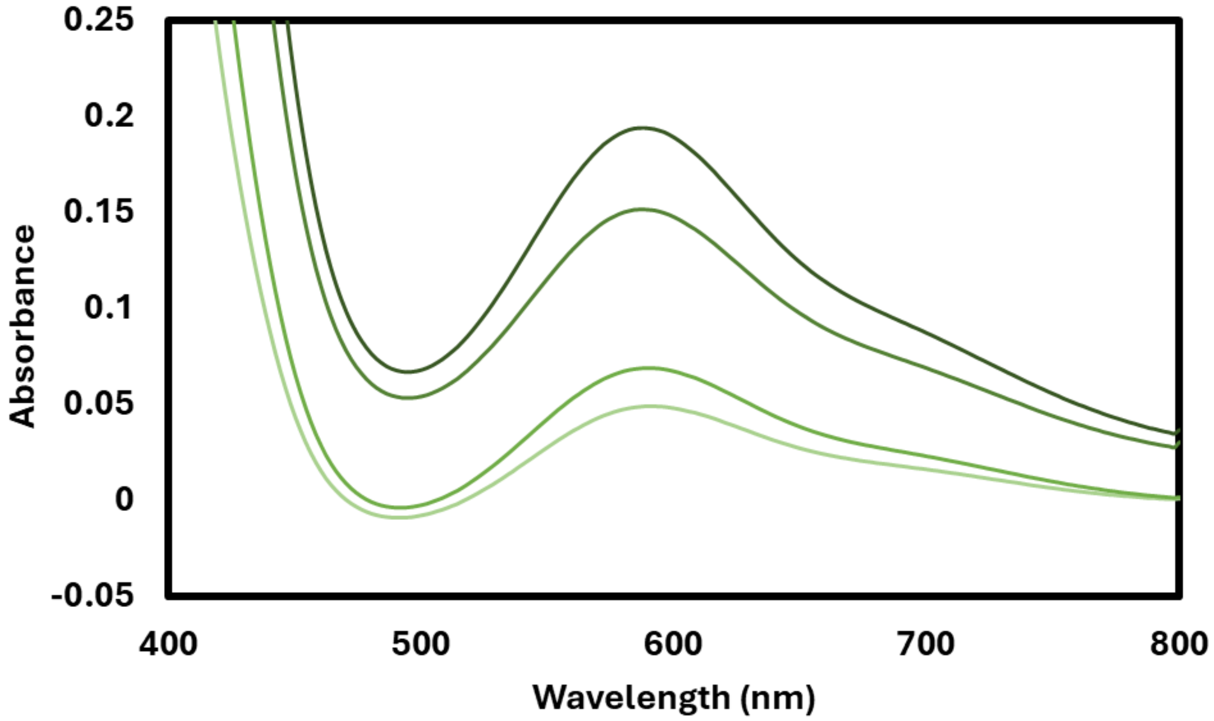

Figure S23: UV-Vis spectra of 0.75 mM, 1 mM, 1.5 mM, and 2 mM solutions of **3-Hf(Mo$_5$)$_2$** in MeCN.

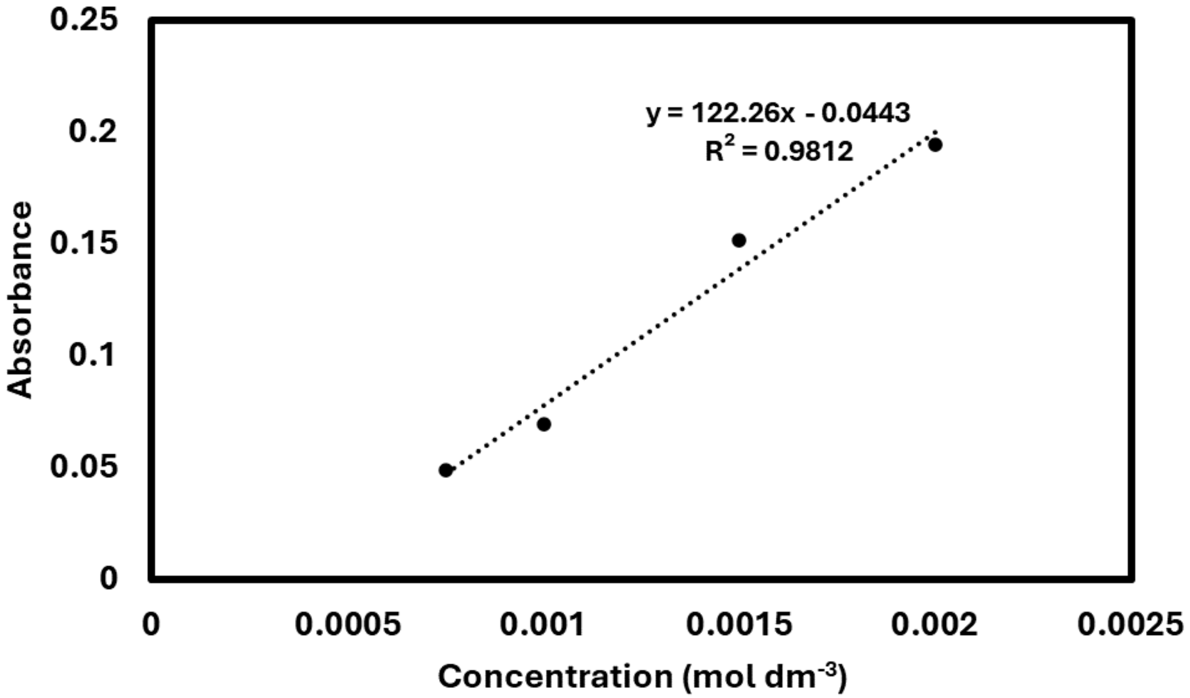

Figure S24: Conc. vs abs. plot at the λ_{max} (588 nm) for **3-Hf(Mo$_5$)$_2$** in MeCN.

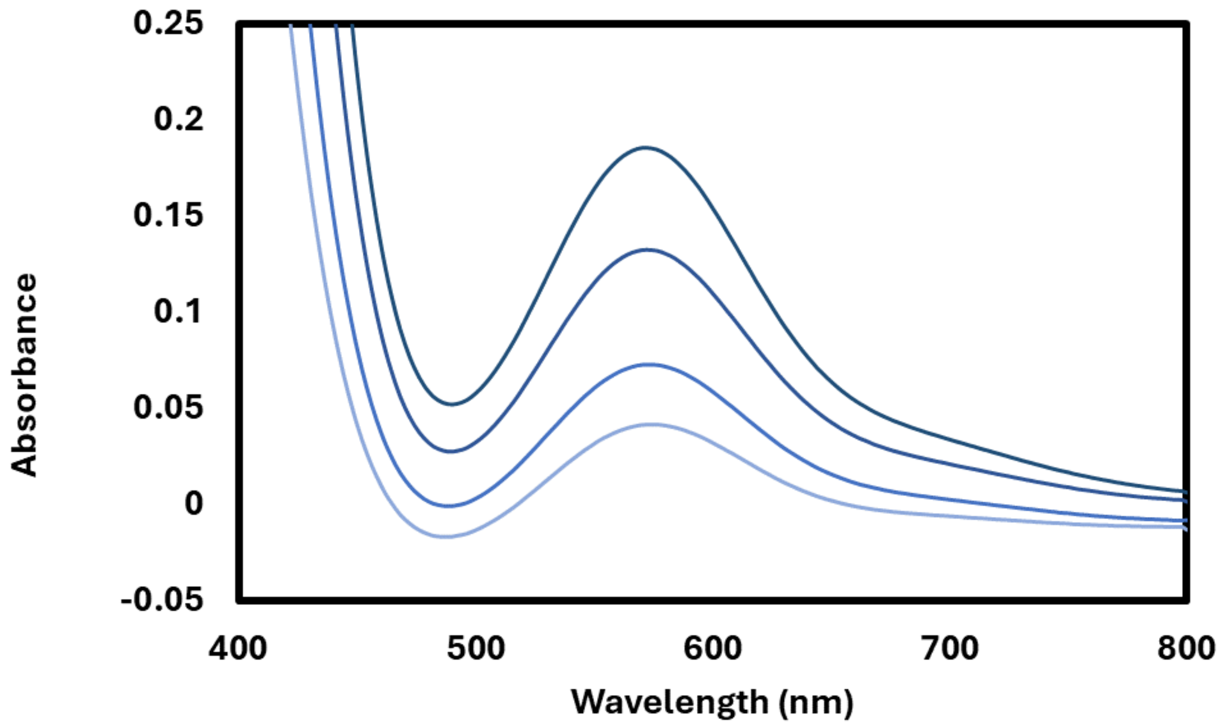

Figure S25: UV-Vis spectra of 0.75 mM, 1 mM, 1.5 mM, and 2 mM solutions of **4-Th(Mo$_5$)$_2$** in MeCN.

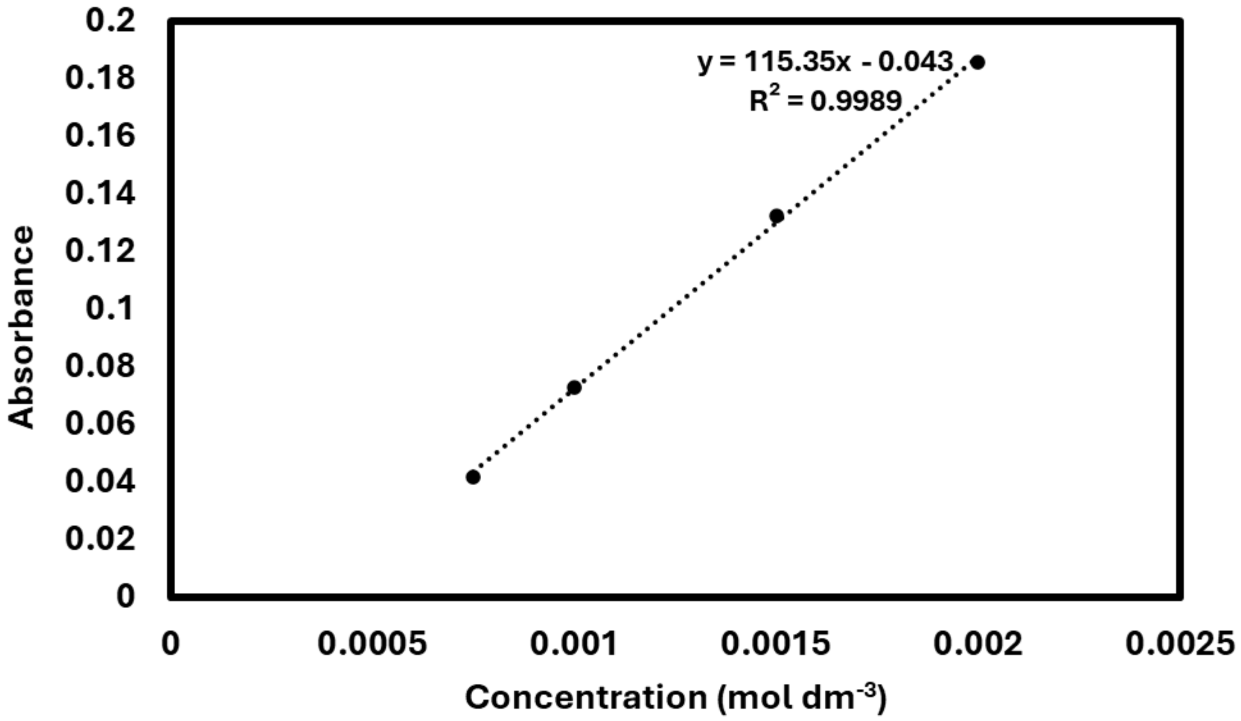

Figure S26: Conc. vs abs. plot at the λ_{max} (572 nm) for **4-Th(Mo$_5$)$_2$** in MeCN.

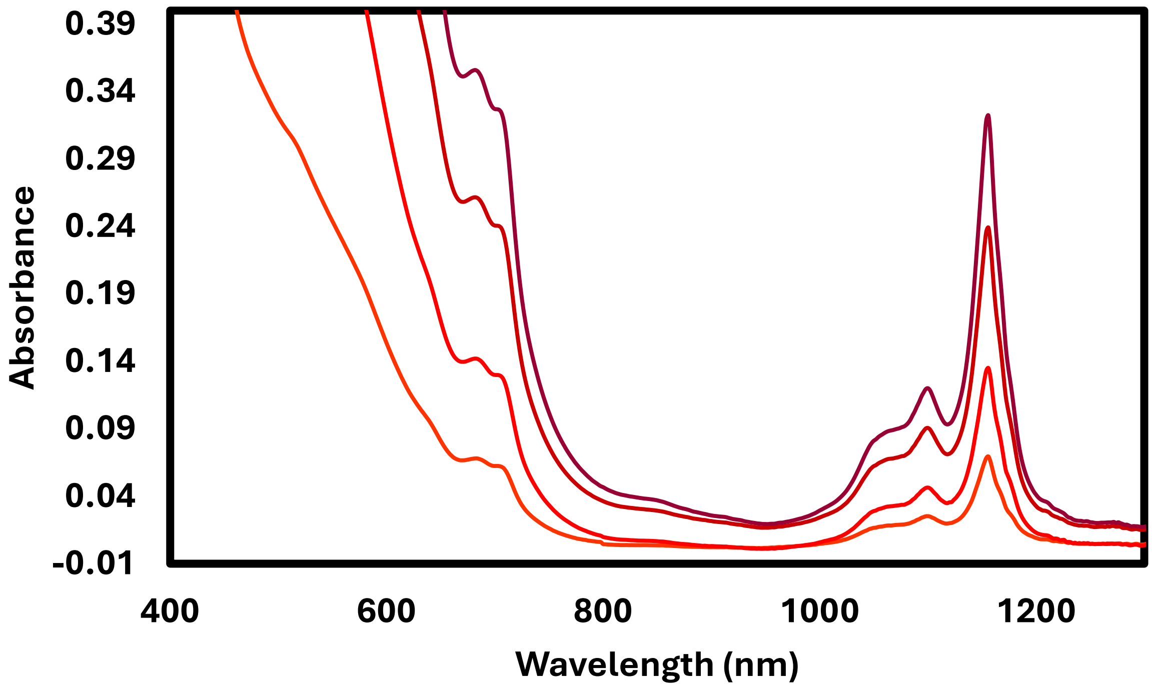

Figure S27: UV-Vis spectra of 0.5 mM, 1 mM, 1.5 mM, and 2 mM solutions of **5-U(Mo$_5$)$_2$** in MeCN.

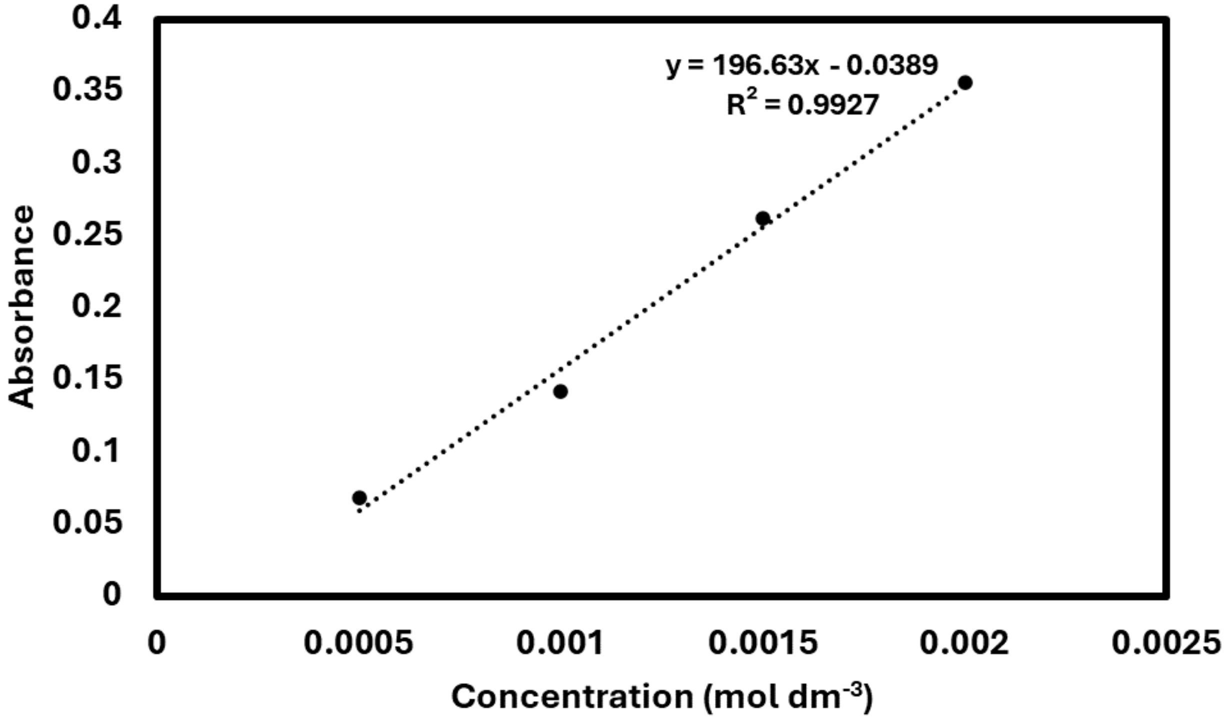

Figure S28: Conc. vs abs. plot for the *f-f* transition at 682 nm of **5-U(Mo$_5$)$_2$** in MeCN.

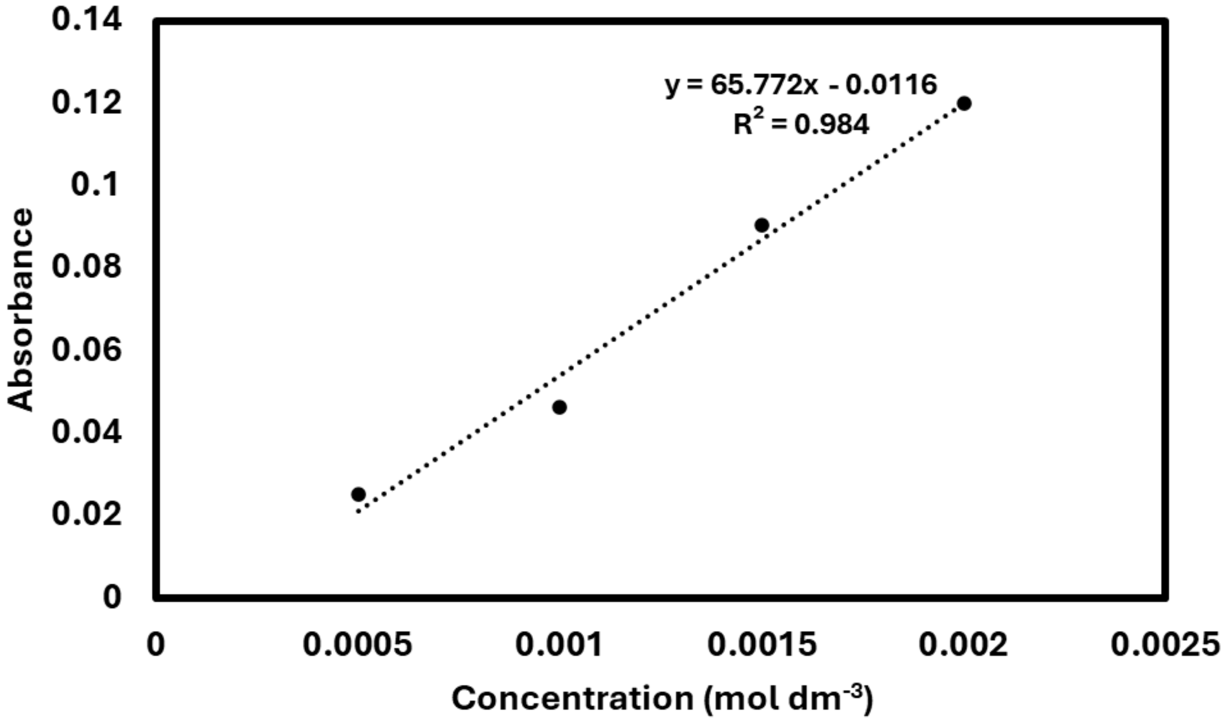

Figure S29: Conc. vs abs. plot for the *f-f* transition at 1100 nm of **5-U(Mo$_5$)$_2$** in MeCN.

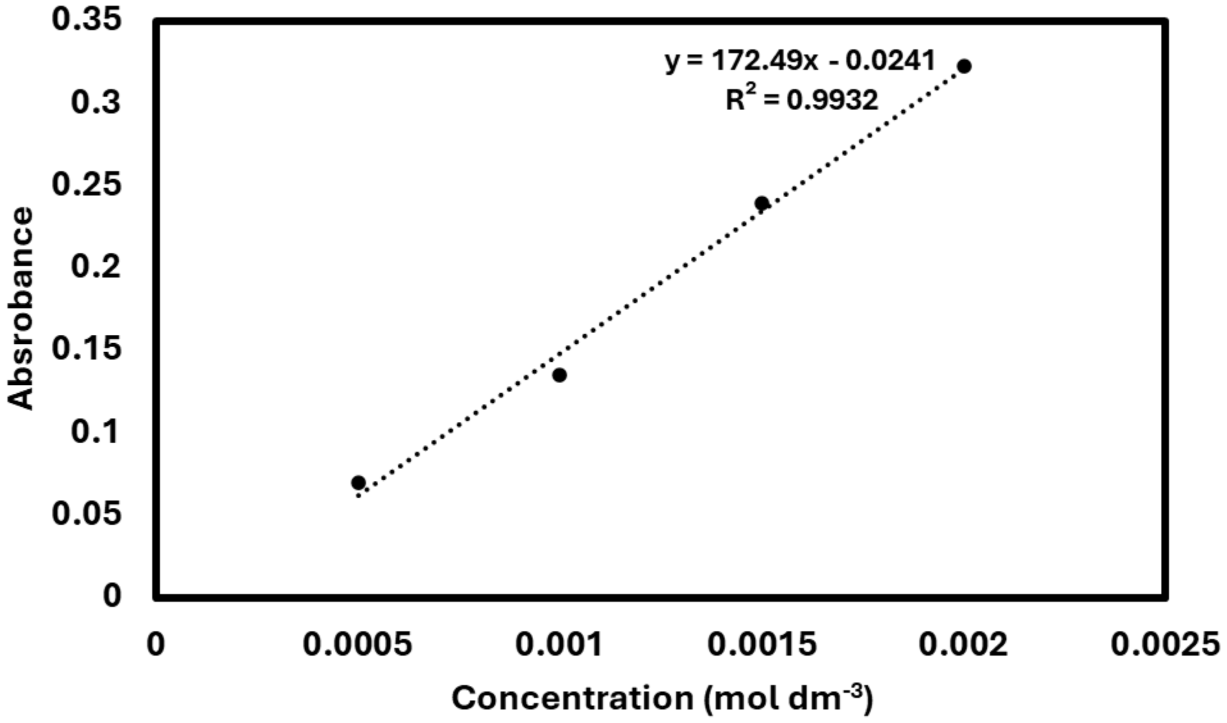

Figure S30: Conc. vs abs. plot for the *f-f* transition at 1156 nm of **5-U(Mo$_5$)$_2$** in MeCN.

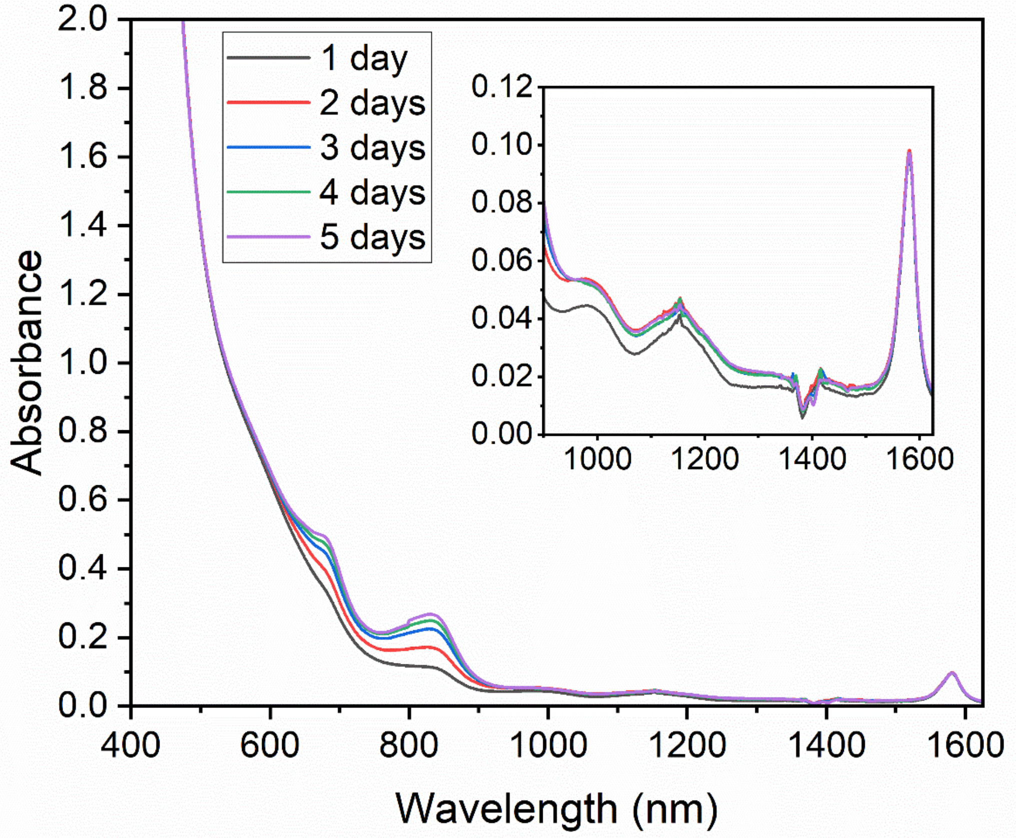

Figure S31: UV-Vis/NIR spectra of **6-U(Mo_5)$_2$** in DCM at room temperature (21 °C) recorded every day for 5 days. The solution was sealed in a screw-top quartz cuvette and was assumed to protected from air and moisture.

3. Single crystal X-ray diffraction information

Table S1: Crystallographic parameters for **2-Zr(Mo$_5$)$_2$** and **3-Hf(Mo$_5$)$_2$**

	**2-Zr(Mo$_5$)$_2$**	**3-Hf(Mo$_5$)$_2$**
Empirical formula	$C_{45.10}H_{106.75}Mo_{10}N_5O_{36.78}Zr$	$C_{45.13}H_{106.82}HfMo_{10}N_5O_{36.78}$
Formula weight	2358.35	2446.14
Temperature	100.00(10) K	100.00(10) K
Wavelength	0.71073 Å	0.71073 Å
Crystal system	Monoclinic	Monoclinic
Space group	$P2_{1/c}$	$P2_{1/c}$
Unit cell dimensions	a = 21.4037(2) Å<br>b = 15.0940(2) Å<br>c = 24.2093 Å<br>α = 90°<br>β = 92.6550(10)°<br>γ = 90°	a = 21.3965(2) Å<br>b = 15.0939(2) Å<br>c = 24.2325(3) Å<br>α = 90°<br>β = 92.6250(10)°<br>γ = 90°
Volume	7812.84(16) Å^3	7817.83(16) Å^3
Z	4	4
Reflections collected	147354	148392
Independent reflections	26637	26606
Goodness-of-fit on F^2	1.046	1.025
Final R indices [I>2sigma(I)]	$R1$ = 0.0445<br>$wR2$ = 0.0891	$R1$ = 0.0394<br>$wR2$ = 0.0779

Table S2: Crystallographic parameters for **4-Th(Mo$_5$)$_2$** and **5-U(Mo$_5$)$_2$**

	**4-Th(Mo$_5$)$_2$**	**5-U(Mo$_5$)$_2$**
Empirical formula	$C_{42}H_{99}Mo_{10}N_5O_{36}Th$	$C_{45.75}H_{108}Mo_{10}N_5O_{36}U$
Formula weight	2441.70	2501.80
Temperature	100K	100 K
Wavelength	0.56087 Å	0.56087 Å
Crystal system	Triclinic	Monoclinic
Space group	*P*-1	$P2_{1/c}$
Unit cell dimensions	a = 12.8161(2) Å<br>b = 15.4649(2) Å<br>c = 19.9194(3) Å<br>α = 87.176(1)°<br>β = 84.075(1)°<br>γ = 73.714(1)°	a = 21.7821(5) Å<br>b = 15.0682(4) Å<br>c = 24.1223(6) Å<br>α = 90°<br>β = 92.925(2)°<br>γ = 90°
Volume	3768.50(10) Å^3	7907.0(3) Å^3
Z	2	4
Reflections collected	27268	17419
Independent reflections	22524	12760
Goodness-of-fit on F^2	1.040	1.061
Final R indices [I>2sigma(I)]	*R*1 = 0.0252<br>*wR*2 = 0.0530	*R*1 = 0.0450<br>*wR*2 = 0.1141

Table S3: Crystallographic parameters for **6-U(Mo$_5$)$_2$**

	**6-U(Mo$_5$)$_2$**
Empirical formula	$C_{26.61}H_{65.22}Cl_{5.22}Mo_{10}N_3O_{36}U$
Formula weight	2385.74
Temperature	100.00(10) K
Wavelength	1.54184 Å
Crystal system	Monoclinic
Space group	$P2_{1/n}$
Unit cell dimensions	a = 16.31000(10) Å<br>b = 21.22060(10) Å<br>c = 18.90170(10) Å<br>α = 90°<br>β = 103.3130(10)°<br>γ = 90°
Volume	6366.22(6) Å^3
Z	4
Reflections collected	109066
Independent reflections	13749
Goodness-of-fit on F^2	1.093
Final R indices [I>2sigma(I)]	$R1$ = 0.0389<br>$wR2$ = 0.1028

Table S4: Average bond length data for the structures discussed. All values in Å. A schematic is given below to highlight bond assignments.

	2-Zr(Mo_5)$_2$	3-Hf(Mo_5)$_2$	4-Th(Mo_5)$_2$	5-U(Mo_5)$_2$	6-U(Mo_5)$_2$
**Mo-O-Mo (eq)**	1.911	1.911	1.904	1.905	1.914
**Mo-O-Mo (ax)**	2.200	2.202	2.204	2.194	2.172
**Mo(II)-O**	2.009	2.009	2.002	2.002	2.015
**Mo-O-M**	1.774	1.774	1.773	1.778	1.803
**M-O**	2.201	2.191	2.410	2.358	2.277
**Mo=O**	1.690	1.690	1.693	1.690	1.681
**Mo-O(μ_5) (eq)**	2.312	2.311	2.323	2.323	2.321
**Mo-O(μ_5) (ax)**	2.093	2.091	2.124	2.117	2.111
**M_5O- μ_5O**	6.652	6.643	6.940	6.843	6.749
**O-O**	2.751	2.751	3.105	3.012	2.889
**Mo-NO**	1.773	1.773	1.776	1.768	1.768

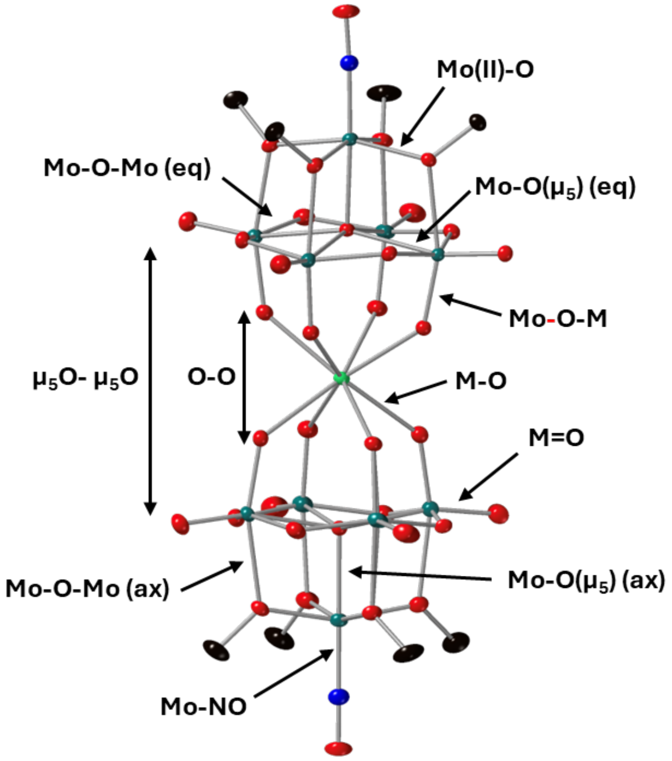

Calculation of U^{5+} ionic radius:

Method 1: Average value obtained from subtraction of O^{2-} ionic radius (1.35 Å)[1] from U-O bond lengths.

U-O bond lengths	O^{2-} ionic radius	Calc. U ionic radius
2.232	1.35	0.882
2.234		0.884
2.36		1.01
2.285		0.935
2.341		0.991
2.307		0.957
2.205		0.855
2.254		0.904
		**0.92725**

Method 2: BVS analysis was performed on **6-U(Mo$_5$)$_2$**. The bond valence sum for the U center was calculated according to previously reported methods[2], using the equations shown below, where V_j is the valence of the jth atom or ion, v_{ij} is the bond valence contribution from the "bond" between the ith and jth atom/ion, R_{ij} is a constant (here it is taken as 2.0935, the average of the U^{IV} and U^{VI} bond valance parameters given in ref. 2) that is dependent on the ij pair, d_{ij} is the observed bond length and b is 0.37.[3]

$$V_i = \sum_j v_{ij} \tag{1}$$

$$v_{ij} = \exp\left[\frac{(R_{ij} - d_{ij})}{b}\right] \tag{2}$$

U-O	e[(R_{ij} - d_{ij})/B]	BVS (V_i)
2.232	0.68775382	4.916087
2.234	0.68404626	
2.36	0.48662072	
2.285	0.59596844	
2.341	0.51226208	
2.307	0.56156541	
2.205	0.73981779	
2.254	0.64805236	

A single average bond length (d_{ij}) was then back calculated by re-arrangement of equation 3 to give equation 4.

$$R_{ij} = b \ln[\frac{V_i}{\sum_j \exp\left(-\frac{d_{ij}}{b}\right)}] \quad (3)$$

$$\sum_j \exp\left(-\frac{d_{ij}}{b}\right) = \frac{V_i}{\exp\left(\frac{R_{ij}}{b}\right)} \quad (4)$$

Plugging in values of R_{ij} = 2.0945, b = 0.37 and V_i = 4.916087 (from BVS calculations) gives:

$$\sum_j \exp\left(-\frac{d_{ij}}{b}\right) = 0.017153 \quad (5)$$

The co-ordination number is 8, so the summation is eight instances:

$$\exp\left(-\frac{d_{ij}}{b}\right) = \frac{0.017153}{8} = 0.002144 \quad (6)$$

And therefore d_{ij} = 2.273664. Subtracting the ionic radius of O^{2-} (1.35 Å)[1] gives an effective ionic radius for the eight co-ordinate U^{5+} center of 0.9236664 Å

Averaging the values obtained by method 1 and 2 gives 0.925457 Å, rounded to 0.93 Å.

References:

1. R. D. Shannon, *Acta Crystallogr. Sect. A: Found. Crystallogr.*, 1976, **32**, 751-767.
2. N. E. Brese and M. O'Keeffe, *Acta Crystallogr. Sect. B*, 1991, **47**, 192-197.
3. I. D. Brown and D. Altermatt, *Acta Crystallogr. Sect. B*, 1985, **41**, 244-247.

4. Electrochemistry

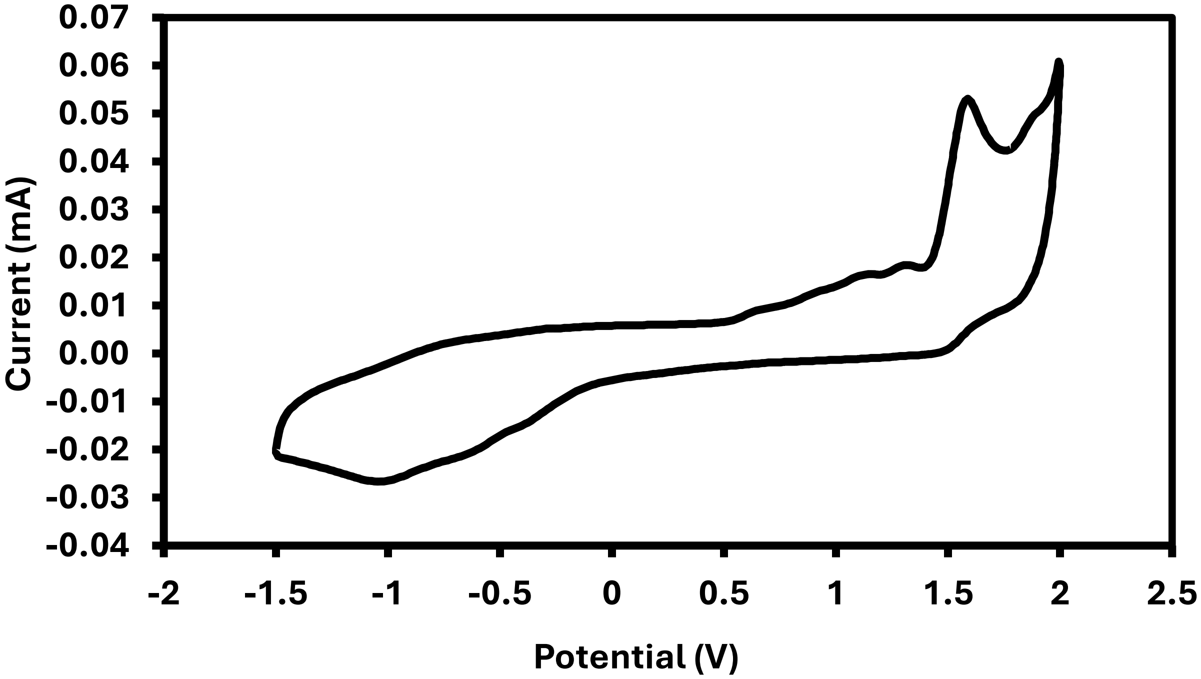

Figure S32: CV of $(TBA)_4[Ba\{Mo_5O_{13}(OMe)_4NO\}_2]$ (1 mM) in MeCN (0.1 M $TBA(PF_6]$). Scan rate = 200 mv/s.

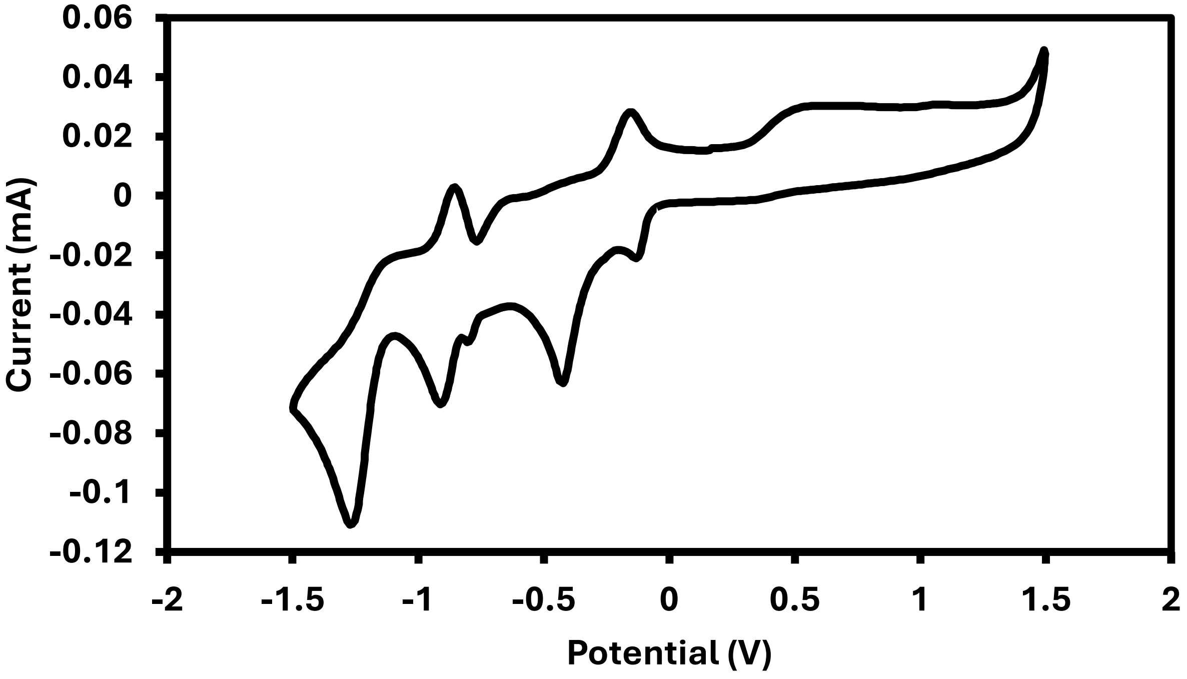

Figure S33: CV of $(TBA)_3[Bi\{Mo_5O_{13}(OMe)_4NO\}_2]$ (1 mM) in MeCN (0.1 M $TBA(PF_6]$). Scan rate = 200 mv/s.

.

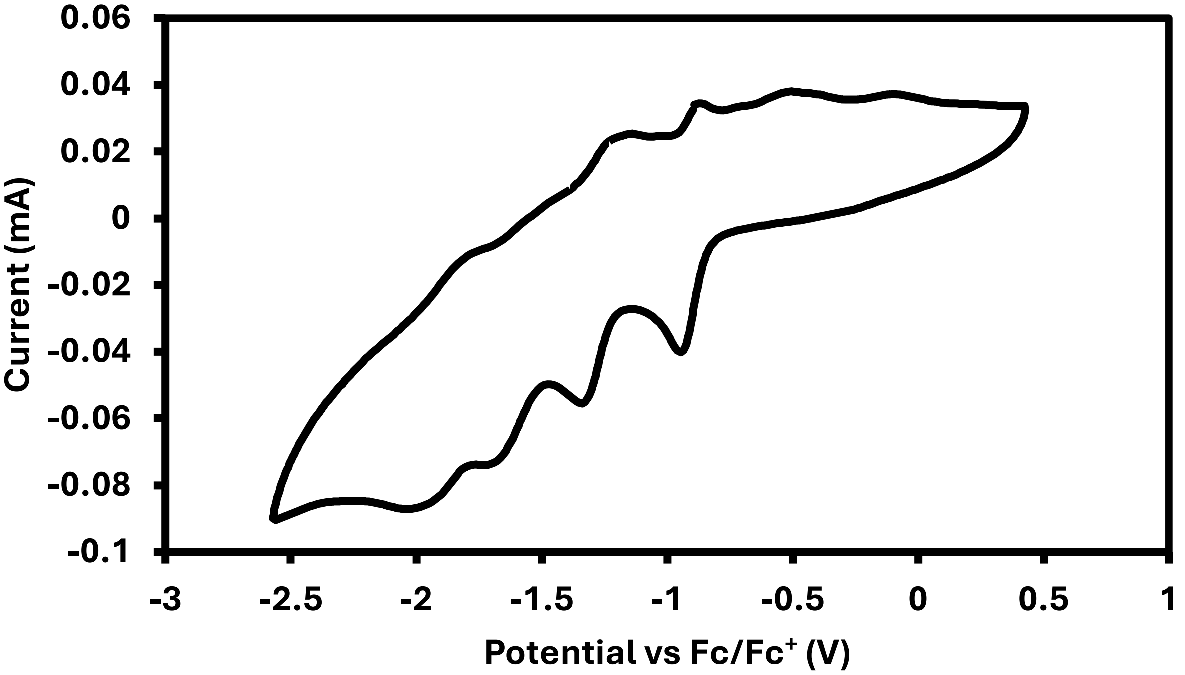

Figure S34: CV of $(TBA)_2[Zr\{Mo_5O_{13}(OMe)_4NO\}_2]$ (1 mM) in MeCN (0.1 M $TBA(PF_6)$) when scanned to more negative potentials. Scan rate = 200 mv/s.

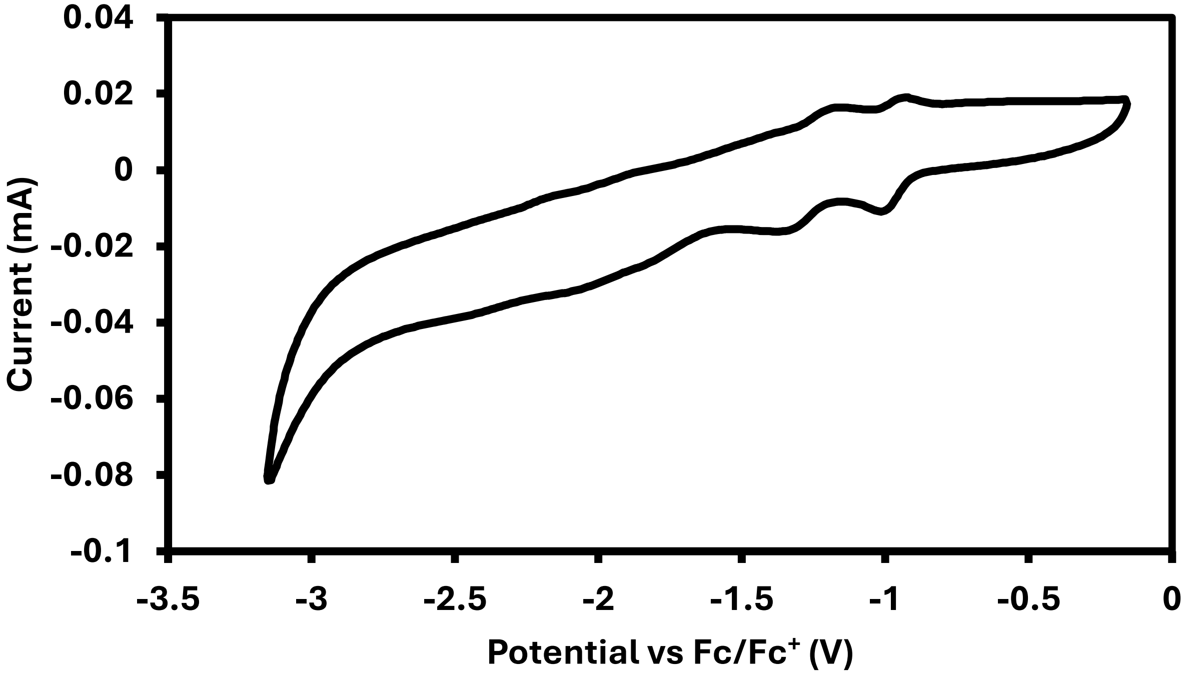

Figure S35: CV of $(TBA)_2[Zr\{Mo_5O_{13}(OMe)_4NO\}_2]$ (1 mM) in MeCN (0.1 M $TBA(PF_6)$) when scanned to more negative potentials. Scan rate = 200 mv/s.

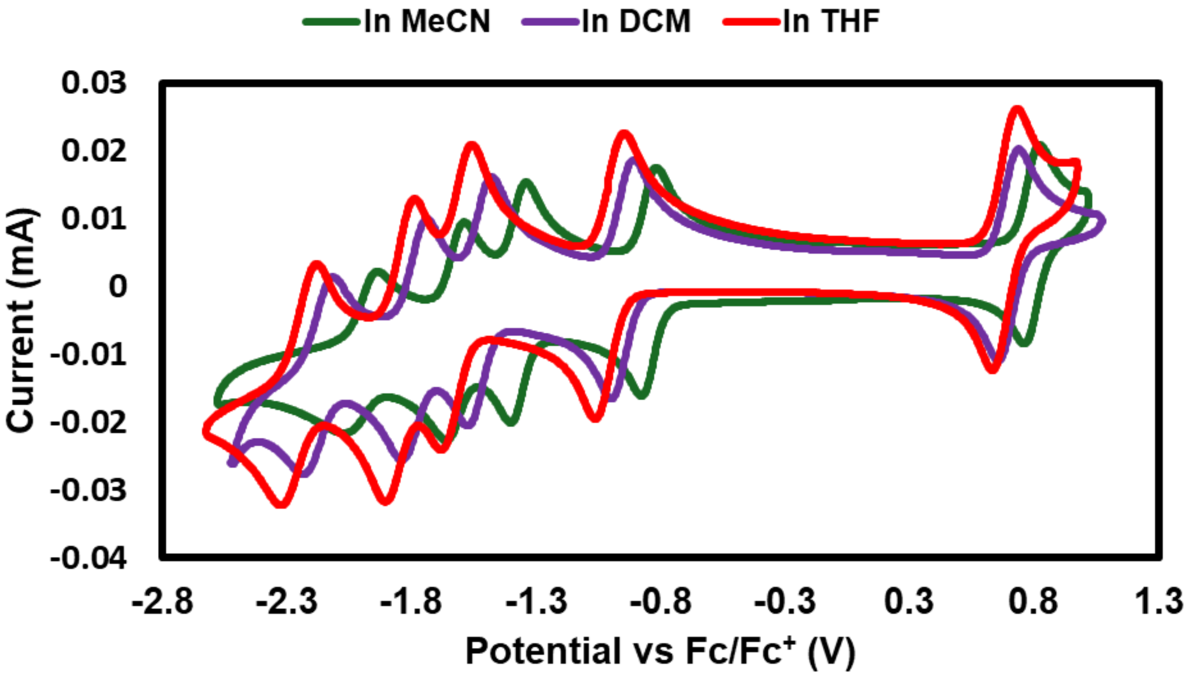

Figure S36: CV of $(TBA)_2[U\{Mo_5O_{13}(OMe)_4NO\}_2]$ (1 mM) in MeCN, DCM, and THF (0.1 M $TBA(PF_6]$). Scan rate = 200 mv/s.

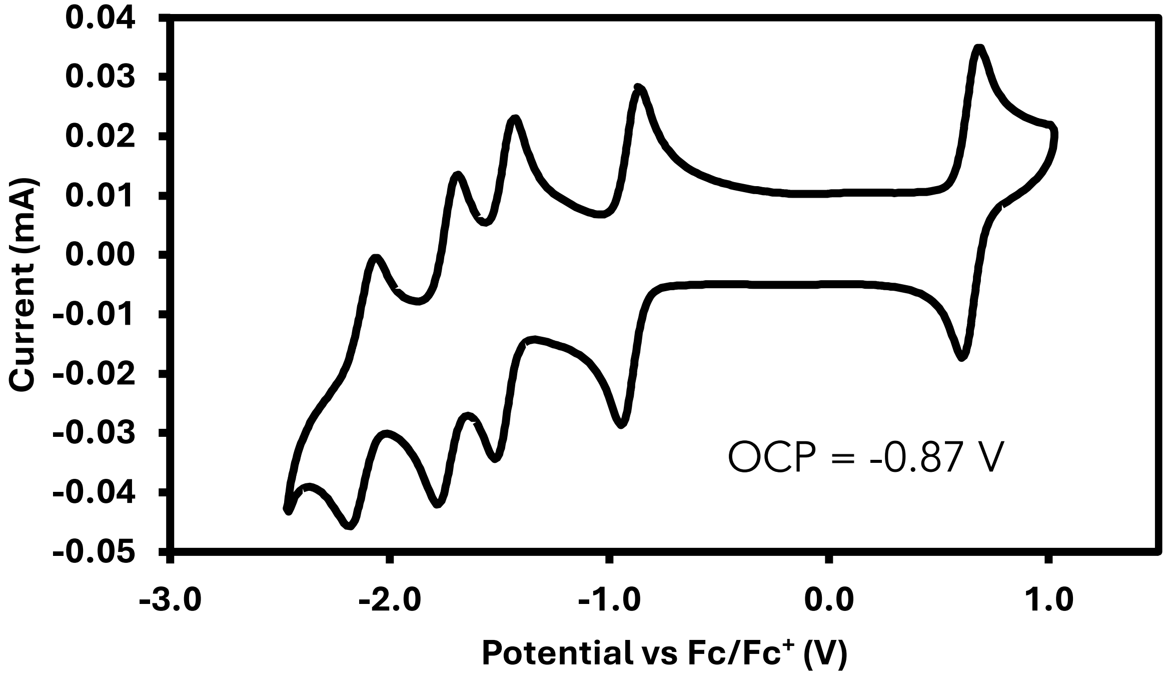

Figure S37: Pre bulk electrolysis CV of $(TBA)_2[U\{Mo_5O_{13}(OMe)_4NO\}_2]$ (1 mM) in MeCN (0.1 M $TBA(PF_6]$). Scan rate = 200 mv/s.

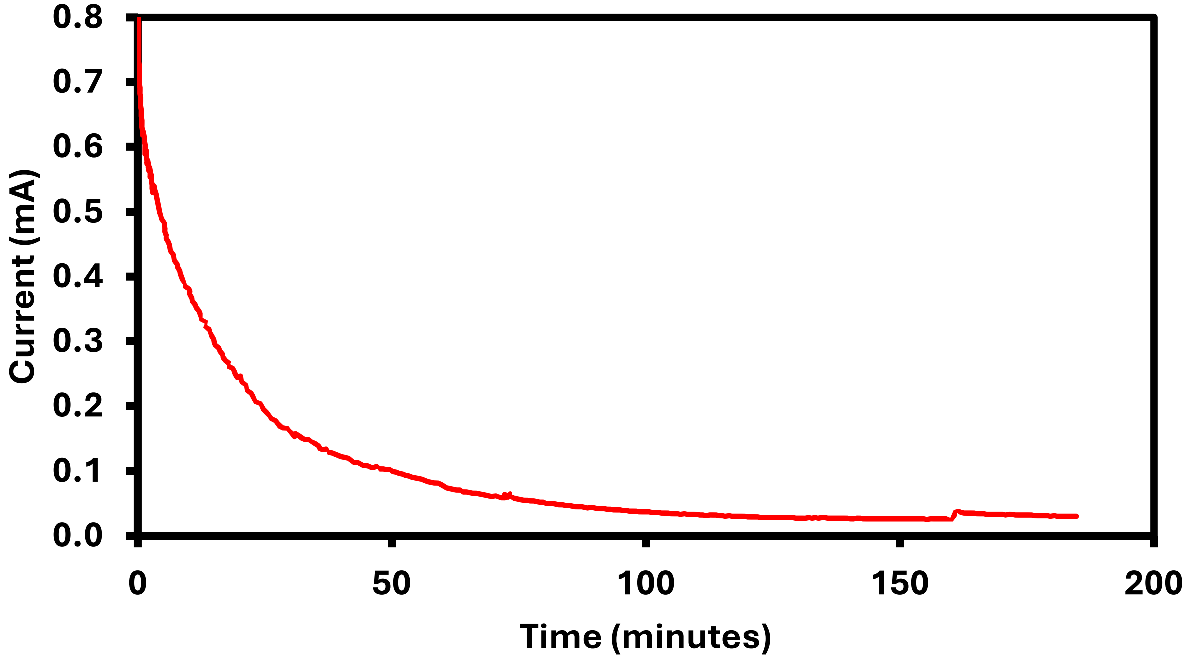

Figure S38: Bulk oxidation of a 1 mM solution of $(TBA)_2[U\{Mo_5O_{13}(OMe)_4NO\}_2]$ in DCM (0.1 M $TBA(PF_6)$. Chronoamperometry was performed at +0.78 V vs Fc/Fc^+.

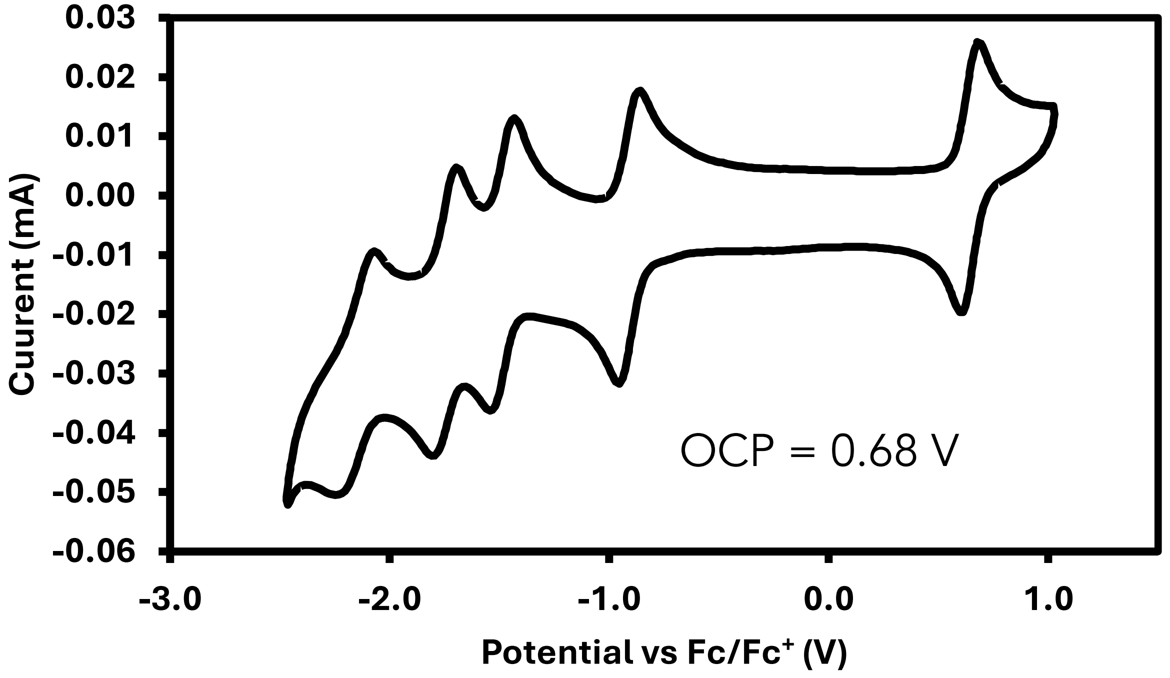

Figure S39: Post bulk electrolysis CV of $(TBA)_2[U\{Mo_5O_{13}(OMe)_4NO\}_2]$ (1 mM) in MeCN (0.1 M $TBA(PF_6])$. Scan rate = 200 mv/s.

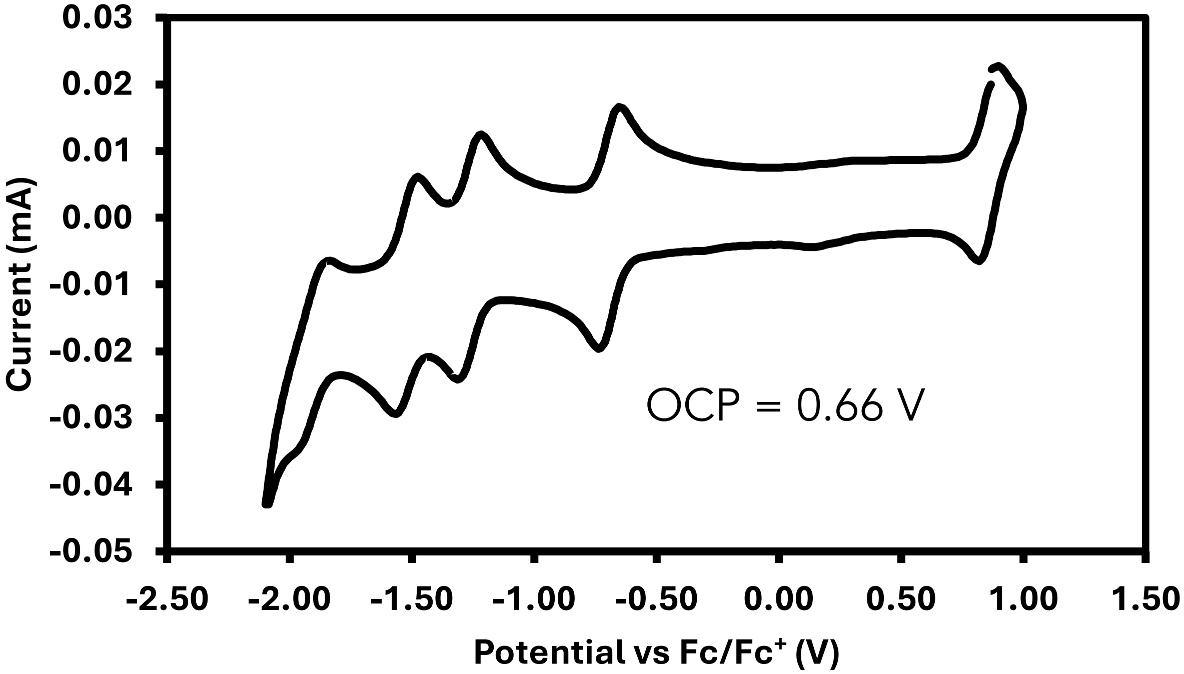

Figure S40: CV of crude (TBA)[U{$Mo_5O_{13}(OMe)_4NO$}$_2$] (1 mM) in DCM (0.1 M TBA(PF_6)) obtained by oxidation of $(TBA)_2$[U{$Mo_5O_{13}(OMe)_4NO$}$_2$] with an excess of [NO][PF_6]. Scan rate = 200 mv/s.
